# Trust and Acceptance Challenges in the Adoption of AI Applications in Health Care: Quantitative Survey Analysis

**DOI:** 10.2196/65567

**Published:** 2025-03-21

**Authors:** Janne Kauttonen, Rebekah Rousi, Ari Alamäki

**Affiliations:** 1 Digital Transition and AI Haaga-Helia University of Applied Sciences Helsinki Finland; 2 Communication Studies School of Marketing and Communication University of Vaasa Vaasa Finland; 3 Faculty of Information Technology University of Jyväskylä Jyväskylä Finland

**Keywords:** artificial intelligence, AI, health care technology, technology adoption, predictive modeling, user trust, user acceptance

## Abstract

**Background:**

Artificial intelligence (AI) has potential to transform health care, but its successful implementation depends on the trust and acceptance of consumers and patients. Understanding the factors that influence attitudes toward AI is crucial for effective adoption. Despite AI’s growing integration into health care, consumer and patient acceptance remains a critical challenge. Research has largely focused on applications or attitudes, lacking a comprehensive analysis of how factors, such as demographics, personality traits, technology attitudes, and AI knowledge, affect and interact across different health care AI contexts.

**Objective:**

We aimed to investigate people’s trust in and acceptance of AI across health care use cases and determine how context and perceived risk affect individuals’ propensity to trust and accept AI in specific health care scenarios.

**Methods:**

We collected and analyzed web-based survey data from 1100 Finnish participants, presenting them with 8 AI use cases in health care: 5 (62%) noninvasive applications (eg, activity monitoring and mental health support) and 3 (38%) physical interventions (eg, AI-controlled robotic surgery). Respondents evaluated intention to use, trust, and willingness to trade off personal data for these use cases. Gradient boosted tree regression models were trained to predict responses based on 33 demographic-, personality-, and technology-related variables. To interpret the results of our predictive models, we used the Shapley additive explanations method, a game theory–based approach for explaining the output of machine learning models. It quantifies the contribution of each feature to individual predictions, allowing us to determine the relative importance of various demographic-, personality-, and technology-related factors and their interactions in shaping participants’ trust in and acceptance of AI in health care.

**Results:**

Consumer attitudes toward technology, technology use, and personality traits were the primary drivers of trust and intention to use AI in health care. Use cases were ranked by acceptance, with noninvasive monitors being the most preferred. However, the specific use case had less impact in general than expected. Nonlinear dependencies were observed, including an inverted *U*-shaped pattern in positivity toward AI based on self-reported AI knowledge. Certain personality traits, such as being more disorganized and careless, were associated with more positive attitudes toward AI in health care. Women seemed more cautious about AI applications in health care than men.

**Conclusions:**

The findings highlight the complex interplay of factors influencing trust and acceptance of AI in health care. Consumer trust and intention to use AI in health care are driven by technology attitudes and use rather than specific use cases. AI service providers should consider demographic factors, personality traits, and technology attitudes when designing and implementing AI systems in health care. The study demonstrates the potential of using predictive AI models as decision-making tools for implementing and interacting with clients in health care AI applications.

## Introduction

### Background

Artificial intelligence (AI) has shown significant potential in health care and well-being. AI applications play a crucial role in the digital transformation of health care [1]. This includes transformations in areas such as medical data analysis, treatment planning, robotic surgeries, clinical operations support, diagnostics, web-based nursing, and connected health care devices [2]. AI enables new methods for how health diagnoses and treatment recommendations are delivered to patients [3]. In addition, technology adds positive effects to health care such as improving availability, accessibility, and efficiency as well as reducing the cost of health care service delivery [4].

### Challenges and the Importance of Trust in AI Adoption

While there are numerous benefits to integrating technology into health care, there are also challenges. Owing to the data-driven and autonomous nature of AI, matters that already posed a concern for health care such as confidentiality (ie, information security and privacy), decision-making, accountability, and responsibility have become more pronounced from a technological perspective. Thus, it is important to examine the ethical and acceptance determinants (including challenges) of AI applications as they are crucial for patient safety and accountability and can significantly boost health consequences [5]. Relating to this, human trust is an *enabler* and a state in which individuals believe that engagement in actions or confidence in others (including objects) will result in a positive outcome [6]. Without assurance that the interests and concerns of humans (patients and professionals) are at the heart of adoption and implementation, human trust—the belief that engagement with a transaction or interaction with the technology will result in positive outcomes [7]—fails to manifest. The current landscape of mass digitalization is rife with scenarios in which individuals are dependent on digitally connected technology without trust (ie, forced digitalization). Trust can be seen as a state of certainty in which the truster believes that the trustee (human, artifact, or service) will act according to promises and expectations [6,8]. Despite the popularity of health care AI scholarship, studies that combine trust with experience of concrete application scenarios are scarce.

Trust can vary dramatically depending on the type of AI system an individual is dealing with, the context, and the level of engagement (ie, queuing for an appointment in a nonemergency situation vs undergoing open-heart surgery). In this domain, human lives and well-being are in question, meaning that the stakes are high for critical AI systems as encountered in medical AI [9]. Trust is closely related to acceptance and intention to use different types of technology, as demonstrated by various studies [10-13]. It is often mediated by perceived usefulness, ease of use, and satisfaction. Applied sociological and economic theories proposed by Pavlou and Gefen [14] revealed that the perceived effectiveness of AI at an institutional level impacted trust at the individual level, both toward the institution and the technology. This perceived effectiveness was based on 2 types of mechanisms—weak (market) and strong (legal). In addition, consideration for behavioral intention based on this perceived effectiveness was incorporated into this study: predicted preference of the technology in the future, the likelihood of using the technology soon, and the intention to use the technology in the future given increased opportunities. Ye et al [15] found that participants with high trust in AI might have high expectations for AI in health care, requiring greater perceived usefulness before engaging with an AI device.

### Factors Influencing Trust and Acceptance

Recent studies highlight the influence of various background variables on attitudes toward AI in health care. Khanijahani et al [16] found that the common determinants for the acceptance of AI technologies are *perceived ease of use*, *usefulness*, *performance*, and *expectancy* among health care professionals and patients. However, as we see from the following examples of scholarship, results vary between studies. For instance, Omrani et al [17] found that men and individuals aged >55 years exhibited higher trust in AI. Conversely, Fritsch et al [18] reported that older patients, women, and those with lower education were more cautious regarding AI in health care. Riedl [9] concludes that older people are increasingly concerned by AI systems. The study by Nadarzynski et al [19] on the acceptability of AI-enabled chatbots in health care revealed several emergent themes—understanding: awareness and experience; AI hesitancy: perceived accuracy, premature technology, nonhuman interaction, and cybersecurity; and motivations: anonymity, convenience, and signposting (using words to guide processes). Individuals’ opinions toward AI were measured through constructs of *perceived accuracy*, *perceptions of premature technology*, *nonhuman interaction*, *cyber security*, and *accessibility*. The findings highlight hesitancy toward chatbots corresponding with the IT skill levels of participants, as well as dislike for talking to computers. Researchers included multiple demographic factors in their analysis but found no significant association with AI acceptability.

Esmaeilzadeh [20] identified positive effects of gender (man), income, education, employment, technical knowledge, and familiarity with AI on the intention to use AI-based health care tools, while age, race, and general computer skills were not significant. On the other hand, studies such as that by Lambert et al [21] noted no gender effects on AI acceptance among health care professionals. Araujo et al [22] observed that age negatively affected perceived usefulness and positively affected the perceived risk of automated decision-making (ADM) by AI. In this instance, gender was seen to influence perceived usefulness but not risk. A study by Ho et al [23], positioned in a Japanese cultural setting, indicated negative perceptions of emotional AI technology among older adult and male patients. Factors behind these results are attributed to the fear of losing control to AI, while due to its cultural context (Japan), privacy was not a concern as there are high levels of trust regarding the standard of care in exchange for disclosing medical details. In another cultural setting (Saudi Arabia), Alanzi et al [24] found that individuals in the age group of 43 to 58 years (generation X) were less accepting of AI, mental health, web-based assistants than younger generations, while Park and Woo [25] reported positive associations between age and both emotional and social reactions toward AI. These findings underscore the complexity of factors (cultural background and otherwise) influencing attitudes toward AI in health care, suggesting that demographic variables play significant roles in shaping perceptions and acceptance of AI technologies.

Personality and technical prowess are also relevant factors for attitudes toward AI [9,18,25-27]. Consensus appears that both agreeableness and openness positively affect trust with weaker support for extraversion. High personal technical affinity has a strong positive impact on AI acceptance in health care [18]. In addition, it appears that less-neurotic people exhibit more trust in AI systems [9]. Park and Woo [25] used Big Five personality traits and personal innovativeness in IT (PIIT) trait, which refers to “the willingness of an individual to try out any new information technology.” Findings indicated that the Big Five traits and PIIT trait contribute significantly to explaining individuals’ attitudes toward AI. For example, those with high extraversion showed rather negative attitudes toward AI, and individuals with high levels of neuroticism displayed more negative emotions toward AI. Those with high agreeableness displayed positive emotions and positive attitudes in the social and functionality dimensions of AI. The PIIT trait had a consistent (positive) effect in predicting all types of attitudes toward AI. For example, those who rated themselves as having poor or moderate IT skills showed lower acceptance of the technology [19]. For patients, higher personal technical affinity increases their perception of AI use in health care [18].

### Strategies for AI Adoption in Health Care

Al Badi et al [28] identified 5 main dimensions and 25 subfactors for the main challenges in adopting AI in health care. Their study revealed that accuracy and privacy as well as security criteria are the most important factors toward improving AI adoption in the health care sector. Safety and security were found to be important factors for the acceptance of AI in health care in several other studies [16,21]. The results showed that general knowledge (education) had a positive association with perceptions of ADM usefulness, while domain-specific knowledge (eg, programming and algorithms) had a positive association with perceptions of ADM usefulness and fairness [22]. Characteristics of individuals and medical characteristics of patients also affect AI acceptance and adoption [16]. The use of AI has had negative consequences on trust in data privacy; patient safety; technological maturity (implementing technologies too early in development); and full automation, which has been the result of a rushed transformation from human-human service to human-technology (digital) service [4]. Patients may distrust AI-based data collection methods as part of diagnoses and treatment information [3], which indicates that there might be a difference between AI applications. The lack of available and easily accessible information may operate as a negative factor, slowing down the acceptance of data-driven services in predictive medicine [29].

Previous research has listed several means to increase user acceptance and adoption of AI in health care [4,30,31]. Kwak et al [31] state that to increase nursing students’ intent to use AI, promoting performance expectancy, effort expectancy, and self-efficacy are essential interventions. These aid in fostering a positive attitude toward AI. In supporting the adoption of AI in health care, research indicates that focus should be placed more on facilitating early adoption and sustainable implementation in the health system. This particularly applies from the user’s perspective [30]. For AI to be successfully adopted in health care, patients and other users must consent to use AI applications [3]. Parvinen et al [29] found that informing potential patients or customers regarding the personal benefits of AI improved their willingness to provide consent to use their genomic data. This shows that a trade-off for benefits would boost the acceptance of using data-driven AI technologies. Positive attitudes toward AI have a substantial impact on the intent to use AI in health care [31]. Positive attitude is decreased by the feeling of losing control to AI systems [23].

Chew and Achananuparp [4] suggest the following improvements to the adoption of AI in health care: enhancing user experience and design, enhancing personalization and customizability, enhancing empathy, and personification of AI-enabled chatbots and education of AI capabilities to the public. Effective governance of AI in health care is a prerequisite to precisely address regulatory, ethical, and trust issues while advancing the acceptance and implementation of AI among patients [5]. Ho et al [23] list the following suggestions that might improve emotional AI perception in patients: explaining how AI tools function (increasing transparency), explaining current legal and ethical safeguards, and describing the role of human professionals in the decision-making process might improve positive attitude. Furthermore, other crucial factors including robust data security, anonymization, and privacy measures as a part of secured data management solutions are crucial for establishing users’ acceptance of AI health care applications [32]. In addition, using multimodality and several sensors for health data collection improves the accuracy and trustworthiness of AI applications [33], thus boosting the user acceptance of AI applications in health care.

### Frameworks, Ethical AI Models, and Survey Design

Efforts have been made to establish frameworks to create actionable principles that relate to *data management, model development*, and *deployment* and *monitoring* [34]. These should exist under the banners of governance (organizational policy) and regulation (legal policy). Such frameworks resonate with the works of Vakkuri et al [35] and Rousi [36]. These can be applied as general guidelines and principles for development, yet context-dependent information must be sought additionally. The priorities and importance of factors affecting acceptance and adoption vary depending on the study. While the previous studies are extensive, we felt that they did not examine the element of trust and the specificities of application context variations to the extent that we are interested in.

For this study, we sought further to find theories and constructs that would enable us to delve deeper and in detail in terms of understanding the varying dynamics of individual perception of AI in health care according to dimensions and context. We used the ECCOLA model [35] and the Robot Governance Model [36] to establish the ethical AI themes and dimensions under examination. The ECCOLA model was developed on 3 prominent AI ethics studies and guidelines: the systematic mapping of global guidelines by Jobin et al [37], revealing 5 ethical principles (justice and fairness, transparency, responsibility, nonmaleficence, and privacy); Ethically Aligned Design by IEEE Xplore [38]; and the Ethics Guidelines for Trustworthy AI by the European Commission [39]. ECCOLA features 8 themes designed to be practically applicable for increasing ethical awareness in AI development processes. These 8 themes are stakeholder analysis, transparency, data, agency and oversight, safety and security, fairness, well-being, and accountability. The Robot Governance Model adds to this in that it depicts the properties (sense, process, and act) of the AI systems; connects these to specific challenges; and then accounts for layers of governance in relation to accountability and responsibility. In other words, these frameworks were chosen for their thoroughness, multidimensionality, and detail regarding applied settings.

The AI ethics–related themes and dimensions were built-in within the questionnaire in relation to constructs probed that were obtained from studies across the disciplines of human-computer interaction, information systems, and digital health. In this study, themes related to data, privacy, accessibility (fairness), well-being (trust and trade-offs), and safety and security (cybersecurity) are seen as integrated into the questions to *lower* the concepts from high-level principles to actionable propositions. The scenarios were used to tangibly depict the AI applications, while the questions featured were based on the Robot Governance Model, specifically focusing on the “systems and artifacts” layer. The AI acceptability study by Nadarzynski et al [19] was instrumental in formulating the constructs on the propensity to trust AI, also stemming from the foundational work by Pavlou and Gefen [14]. These were also complemented by other constructs obtained from the study by Cheung and To [40] investigating the propensity to trust in-app advertisements.

Using the aforementioned approach with a robust machine learning–based analysis, we established an understanding of the antecedents for trusting and using AI in specific health care use cases. Our main research questions were as follows:

1. How does context affect an individual’s willingness to trust and accept AI in health care?
2. How does the perceived level of risk (physical, psychological, and social vulnerability) affect an individual’s propensity to trust and accept AI in specific health care settings?

By addressing these questions, we aim to contribute to a deeper understanding of trust dynamics in AI health care applications, ultimately informing strategies for enhancing user acceptance.

## Methods

### Overview

Our methodological approach aimed to investigate public opinions on AI applications in health care and well-being among consumers. We used a web-based questionnaire and established theoretical frameworks from human-computer interaction, information systems, and digital health. The survey comprised demographic questions, evaluations of 8 AI use cases, and general opinions on AI in health care. After data collection, data preprocessing was conducted to ensure the quality and variability of responses. This was followed by confirmatory factor analysis to identify underlying constructs within the data. For predictive modeling, we used the CatBoost gradient boosted tree regression algorithm to capture nonlinear relationships and complex interactions. We applied the Shapley additive explanations (SHAP) technique to investigate model predictions for each predictor and their interactions. This multifaceted approach enabled us to effectively explore the factors influencing individuals’ attitudes toward AI in various health care contexts.

### Web-Based Survey Development and Distribution

Our web-based survey contained three parts:

1. Demographic and personal information (31 questions)
2. Opinions toward AI in health care and well-being for 8 use cases, from which 5 were randomly selected per participant to avoid waterfall effects (21 questions per use case)
3. General opinions of AI in health care and well-being (31 questions)

Questions in parts 2 and 3 were considered response variables, while questions in part 1 were considered predictor variables. For the development of the questions, we used frameworks developed by Vakkuri et al [35] and Rousi [36]. Our theoretical constructs were drawn from human-computer interaction [40], information systems [14], and digital health [19]. We adapted the work by Pavlou and Gefen [14] on the *propensity to trust* [19], characterized by *ease to trust*, *tendency to trust*, and *willingness to trust* with the assumption that main respondents would not be of expert level in terms of understanding how AI systems operate. For the intention to use AI, we adapted the Unified Theory of Acceptance and Use of Technology model [41], which has been adapted by various authors [10,11,13,20,24]. The question related to exercising habits was adapted from official well-being surveys by the Finnish Institute for Health and Welfare [42]. For personality traits, we applied a 10-question battery by Gosling et al [43]. Education and working fields were based on official categories by the Statistics Finland department. The remaining questions related to interest and knowledge of technology and the state of health were self-developed. Refer to Multimedia Appendix 1 [10,11,13,14,19,20,24,35,36,40,41] for detailed information on questions-related sources and how we combined them into factor constructs (Tables S1 and S2 and Figure S1 in Multimedia Appendix 1).

Overall, each respondent was asked to answer 167 questions, which typically took 15 to 20 minutes. Quantitative questions were numerical, single, and multiple choice as well as Likert type. In addition, respondents were allowed to respond via open-text questions (eg, feedback). The survey was implemented using LimeSurvey (version 6.0; LimeSurvey GmbH). Data were collected between May 12, 2023, and May 23, 2023, using a survey pool. A total of 1125 people responded to the survey, out of which a smaller subset (1100/1125, 97.78%) finished the survey fully or partially. A printed version of the survey can be found in Multimedia Appendix 2. Data were collected by a third-party service with access to a large pool of Finnish consumers, with each responder receiving a small monetary reward. The survey was offered both in Finnish and English.

We selected 8 AI use cases, which was considered maximum while still keeping the number of questions and response time reasonable. The criteria for choosing those use cases were to obtain a versatile description of the various AI tools in different contexts, ranging from self-care to hospital environments. As stated by Riedl [9], trust can vary strongly depending on what kind of AI system a user is dealing with (critical vs noncritical). On the basis of the variables of the frameworks [35,36], we selected applications that described different use cases where these aspects appeared differently depending on the use contexts. Thus, variables such as the sensitivity of the data being processed and the level of human agency and oversight associated with the AI-generated advice or actions were considered. The 8 AI use cases featured in the survey were as follows (short form in parenthesis):

1. Activity monitoring AI system to support a healthy lifestyle (*activitymonitor*)
2. Menstrual cycle monitoring and prediction AI system (*menstrual*)
3. AI-controlled robotic surgeon (*robotsurgeon*)
4. Nutrition monitoring and planning AI system (*nutrition*)
5. Real-time health monitoring, analysis, and prediction AI system (*healthmonitor*)
6. Mental health and well-being AI system (*mentalmonitor*)
7. Bioelectronic real-time health monitoring and adjustment AI system (*bioelectric*)
8. AI-controlled robotic nursing and caregiving system (*nursing*)

**Figure 1:**
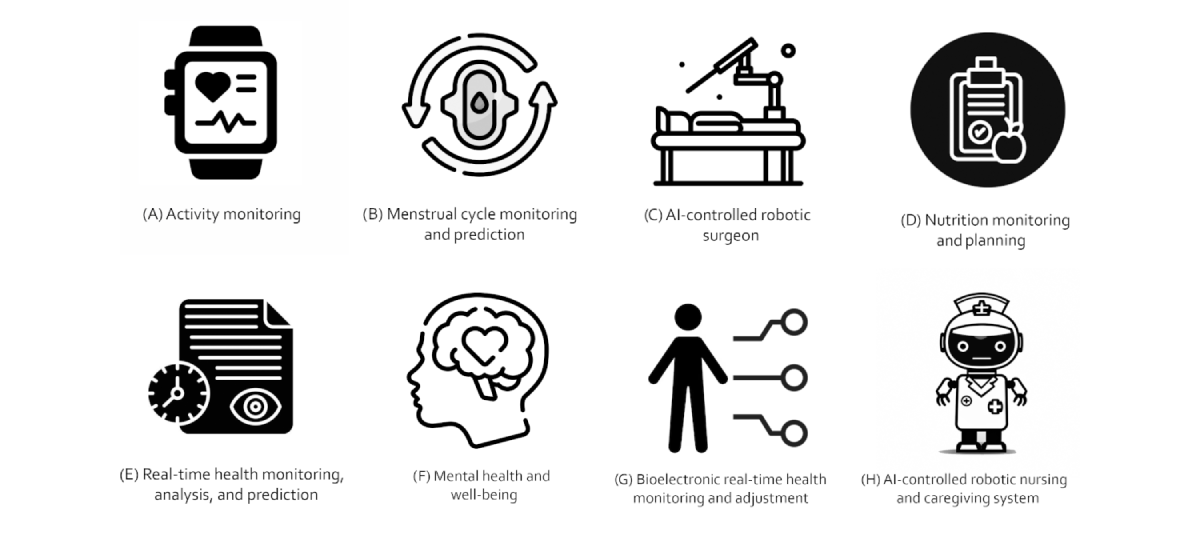
The 8 use cases from the health care and well-being domain included in the survey. These include both invasive and noninvasive cases that are either already available (eg, activity monitoring), emerging, or futuristic (eg, robotic surgeon and nursing system). AI: artificial intelligence.

Use cases are illustrated in Figure 1 with detailed descriptions as present in the survey shown in Multimedia Appendix 3.

### Data Preprocessing and Factor Constructs

Response data were converted into a table with rows representing responses from participants. Responses from part 1 of the survey were converted into the following variables in the 3 main groups. Variable abbreviations with descriptions related to basic demographics and personality types are as follows (variables and variable types are provided in parenthesis):

- *age*: age in years (numerical)
- *gender*: gender identity with 3 options (categorical)
- *education_level*: the highest level of education obtained with 5 options (categorical)
- education_field_* and work_sector_*: field of education and current work sector including education (*education*); commerce, administration, law, or services (*business_services*); science, information and communication technology, or engineering (*science_ICT_engineer*); health and wellness (*health*); and all others (*other*); and all with yes or no options (binary)
- persona_*: 10 personality dimensions with questions critical or quarrelsome (*critical_quarrelsome*), dependable or self-disciplined (*dependable_selfdisciplined*), anxious or easily upset (*anxious_easilyupset*), open to new experiences or complex (*newexperiences_complex*), reserved or quiet (*reserved_quiet*), sympathetic or warm (*sympathetic_warm*), calm or emotionally stable (*calm_stable*), conventional or uncreative (*conventional_uncreative*), disorganized or careless (*disorganized_careless*), and extraverted or enthusiastic (*extraverted_enthusiastic*), all with 7 Likert-scale options (ordinal)

Variable abbreviations with descriptions related to technology use, knowledge, and attitudes are as follows:

- *IT_skills*: self-evaluated IT skills compared to others with 5 options (ordinal)
- *technology_adoption*: self-evaluated adoption and use of new technology with 3 options (categorical)
- *technology_usage*: frequency of using technology to monitor own health and well-being with 5 options (ordinal)
- *AI_knowledge*: self-evaluated knowledge and experience of AI, a summary construct (numerical)
- *technology_attitude*: attitude toward technology, a factor construct (numerical)

Variable abbreviations with descriptions related to personal health, opinion on health care services, and physical activity are as follows:

- *health_status*: self-evaluated overall health status in the past 12 months with 5 options (ordinal)
- *health_service_usage*: frequency of health services use in the past 12 months with 5 options (ordinal)
- *health care_services*: how does one feel about the current state of health care services (private and public sector in Finland) with 5 options (ordinal)
- *exercise_level*: the amount of exercise and physical straining of oneself in free time with 4 options (categorical)

In addition, the variable *use_case* included 8 options. Detailed variable listings can be found in Table S1 in Multimedia Appendix 4, including a listing of all response options.

During preprocessing, participants who did not proceed to part 2 of the survey were removed. Furthermore, participants whose data lacked variability were excluded based on the assumption that they were mostly motivated by the reward without putting real effort into their responses or showing signs of survey fatigue. The criteria for the variability were evaluated for Likert-type questions, entailing that the minimum allowed SD was 0.15. Furthermore, we excluded participants whose responses were too extreme without using the Likert scale properly. For this, we set a threshold of 90%, and responses surpassing this ratio were excluded. These criteria were applied individually for use cases (part 2) and part 3 of the survey to avoid the removal of too much data. Ordinal categorical responses (eg, Likert type) were converted into numerical integer values using the conversion rules listed in Multimedia Appendix 4. After this initial preprocessing, *Lavaan* (version 0.617) [44] confirmatory factor analysis for R (R Foundation for Statistical Computing) was applied to create factor constructs from the Likert-type question batteries in parts 2 and 3 of the survey, as well as 1 question from part 1. The “*cfa*” function was applied with a maximum likelihood estimation algorithm. The model’s reliability was evaluated by calculating the comparative fit index, Tucker-Lewis Index, and root mean squared error. Internal consistency of factors was evaluated by computing the omega, alpha, and average variance explained [45], making sure our factor constructs were reliable and within acceptable limits [46-48]. See Multimedia Appendix 1 for details.

### Regression Modeling With Gradient Boosting

A CatBoost gradient boosted tree regression algorithm with multivariate root mean squared error loss was used to train predictive models [49] for parts 2 (model l) and 3 (model 2) of the survey. CatBoost builds an ensemble of decision trees, where each tree learns to improve prediction accuracy by correcting errors made by the previous trees. CatBoost can natively handle multivariate responses with both numerical and categorical predictor variables, as well as missing values. It can also approximate nonlinear relationships and complex, multilevel interactions, which are not feasible with linear models. Gradient boosting has been successfully applied to modeling in various fields, including behavioral [50] and health sciences [51].

For result interpretations and model analysis, we applied the SHAP method [52]. It is a method for interpreting machine learning models by calculating SHAP values, which quantify the contribution of each feature to the model’s predictions, such as tree-based gradient boosting methods, including CatBoost. On the basis of game theory, the SHAP algorithm assigns each feature a contribution score that indicates how much it influences a prediction. For tree models, the SHAP algorithm efficiently calculates these contributions by breaking down the model’s complex decision processes into simpler, additive components. This approach helps reveal which features have the greatest impact on predictions, allowing us to see not only the individual contributions but also how features interact with each other in determining outcomes. Tree SHAP analysis provides both local (sample level) and global (average) interpretations. Locally, it explains individual predictions, which can help understand individual participants. Globally, by aggregating these explanations across the dataset, the SHAP algorithm enables understanding of broader model behavior and identifying trends or dominant features [52]. In this study, both the main effects of each variable and the interaction of pairs of variables were investigated. While the main effects estimate how individual variables affect the predictions, the interaction effect is the additional combined feature effect after accounting for the main effects, thus giving more nuanced information. Interactions subtract the main effect of the features to obtain the pure interaction effect after accounting for individual effects. We report both mean and mean absolute SHAP values for features defined as follows:



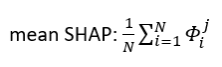



**(1)**



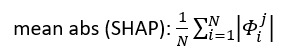



**(2)**

where 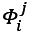. is a (local) SHAP value of sample *i* of feature *j* and *N* is the number of samples.

The Optuna hyperparameter optimization framework [53] was applied to find optimal CatBoost parameters for the data. Optuna is an automatic hyperparameter optimization software framework, particularly designed for machine learning purposes to optimize parameters that control the learning process. For this, the data were randomly split by setting 15% (165/1100) for testing, 15% (165/1100) for development, and the remaining 70% (770/1100) for training. Each participant was present in only 1 single split to maximize predictive power for new participants. Parameters were optimized via Optuna for the development set, and then a model was fitted using both training and development sets. Optuna was again applied in finding parameters that maximized and minimized predictions to allow illustrative examples. Finally, trained models were applied to the held-out separate test data. The performance of the model was assessed by computing the root mean squared error of the predictions, comparing that against a constant-only model, and computing Pearson *R*^2^ statistics for test data. We applied a permutation testing procedure to evaluate whether *R*^2^ values were notably different from 0 [54]. In this procedure, data were randomly shuffled multiple times to obtain an empirical distribution of null values that were compared against the original (unshuffled) values to estimate *P* values.

### Ethical Considerations

This study followed ethical standards and handled data with care. The research involved secondary analysis of survey data purchased from a Finnish third-party company, which is compliant with Finnish legislation. The data were collected anonymously, with no personal identifiers, so additional ethics review was not required. The third-party company obtained informed consent from participants, who willingly completed the survey after being informed of its purpose and use. Consent included provisions for secondary analysis of anonymized data by the researchers. While the third-party company had access to responder identities to ensure sample coverage, they had no access to survey responses and vice versa for the researchers. This system ensured the anonymity of responses. Potentially sensitive questions, that is, those related to personal health, were optional and skippable for the responders. All participants were compensated for their time per the company guidelines and agreements with panel members. No identifiable information or imagery is included in the manuscript or Multimedia Appendices 1-8.

## Results

### Measurement Constructs for the Use of AI for Use Cases

The number of valid data points was 5146 with 1100 participants. The confirmatory factor analysis for response variables (21 for each use case) resulted in a model that covered 19 items in 7 constructs. For all fit parameters and items, refer to Multimedia Appendix 1 with descriptive analysis. The 7-factor construct and their interpretations were as follows:

- *Intention* (3 items): intention to use the AI system if offered the chance
- *Trust* (3 items): trust that the AI system can make valid and accurate decisions
- *Predictions* (3 items): the importance of knowing how results were produced
- *Data* (3 items): the importance to know what type and size of training data were used
- *Privacy* (3 items): the importance to know how personal data are stored and used
- *Trade-off* (2 items): willingness to share personal data to improve AI predictions
- *Manufacturer* (2 items): importance to know the company developing the AI system

All factors were coded so that higher values indicate higher agreement, that is, toward the *strongly agree* direction in the Likert scale to aid interpretability. Many constructs for responses were highly correlated, particularly intention and trust (0.808), predictions and data (0.840), manufacturer and data (0.794), and manufacturer and predictions (0.752). All correlations are listed in Table 1.

All 33 predictor variables used in the analysis are listed in Multimedia Appendix 4.

**Table 1:** Correlation coefficients (n=5146 data points with 1100 participants) between 7 responses (factors) for AI use cases (part 2 of the survey).

			Intention		Manufacturer		Trade-off		Privacy		Data		Predictions		Trust
**Intention**															
	*r*	1		0.022		*0.553^a^*		–*0.106*		–*0.077*		*0.14*		*0.808*	
	*P* value	—^b^		.11		<.001		<.001		<.001		<.001		<.001	
**Manufacturer**															
	*r*	0.022		1		–*0.136*		*0.69*		*0.794*		*0.752*		–*0.053*	
	*P* value	.11		—		<.001		<.001		<.001		<.001		<.001	
**Trade-off**															
	*r*	*0.553*		–*0.136*		1		–*0.468*		–*0.216*		–0.033		*0.578*	
	*P* value	<.001		<.001		—		<.001		<.001		.02		<.001	
**Privacy**															
	*r*	–*0.106*		*0.69*		–*0.468*		1		*0.708*		*0.613*		–*0.228*	
	*P* value	<.001		<.001		<.001		—		<.001		<.001		<.001	
**Data**															
	*r*	–*0.077*		*0.794*		–*0.216*		*0.708*		1		*0.84*		–*0.174*	
	*P* value	<.001		<.001		<.001		<.001		—		<.001		<.001	
**Predictions**															
	*r*	*0.14*		*0.752*		–0.033		*0.613*		*0.84*		1		*0.044*	
	*P* value	<.001		<.001		.02		<.001		<.001		—		.004	
**Trust**															
	*r*	*0.808*		–*0.053*		*0.578*		–*0.228*		–*0.174*		*0.044*		1	
	*P* value	<.001		<.001		<.001		<.001		<.001		.004		—	

^a^All italicized values were significant at *P*<.01, using a 2-tailed permutation test against 0 (5000 iterations).

^b^Not applicable.

### Predictive Modeling With Gradient Boosting

For the predictive model 1 (data from parts 1 and 2 of the survey), we trained a CatBoost model for all 7 response factors. The correlation coefficients for training and testing data were 0.664 and 0.324, corresponding with *R*^2^ values of 0.441 and 0.105. See Table S1 in Multimedia Appendix 5 for detailed information on individual use cases and responses. The difference between training and testing sets indicates some potential overfitting during training. However, our model predicted >10% variance for the testing data and surpassed the chance level (*P*<.001; permutation test with 5000 iterations); thus, we considered it suitable for further analysis. For model 2 (data from parts 1 and 3 of the survey), the procedure was similar. We trained a CatBoost model for intention and trust responses. The final model reached *R*^2^ of 0.225 with 0.235 for intention and 0.220 for trust. See Figures S1-S3 and Table S1 in Multimedia Appendix 6 for full results for model 2.

In the following subsections, SHAP values are reported. The SHAP values were computed using a trained model applied to all available data. First, we investigated the overall importance of features for all 7 responses. Then, we concentrated on intention, trust, and trade-off, for which model 1 reached the best predictive accuracies.

### Combined Feature Importance for Use Cases

**Figure 2:**
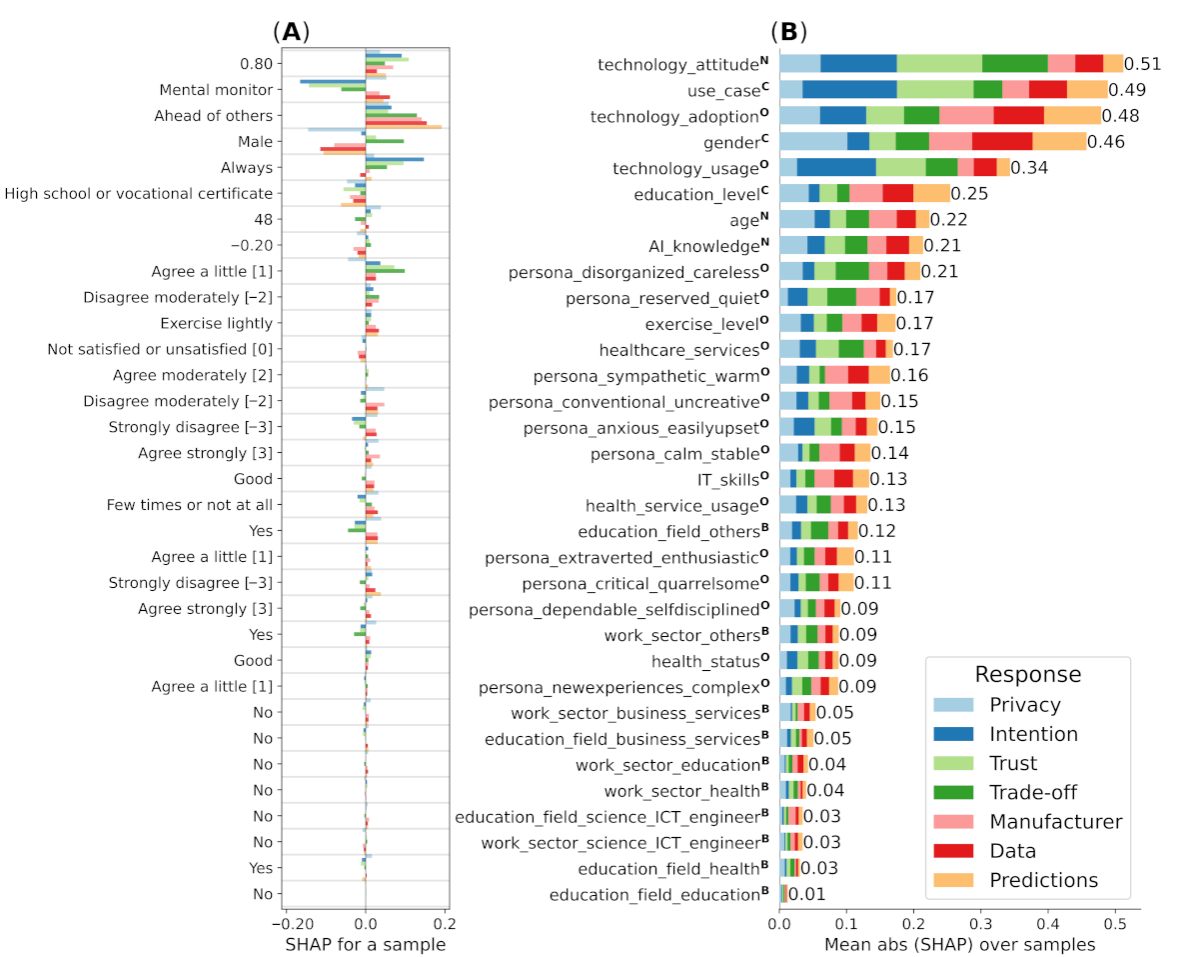
Impact of all 33 features on the 7 response variables measured via Shapley additive explanations (SHAP) main effect values. (A) Single representative sample with SHAP values of each feature contributing to the 7 response predictions. Values in parenthesis correspond to numerical Likert encodings. (B) Mean absolute values of SHAP values over all samples (n=5146 data points with 1100 participants) for each response stacked together for all 7 responses. Bars represent the relative impact of each feature for model predictions. B: binary (yes or no); C: categorical; ICT: information and communication technology; N: numerical; O: ordinal categorical.

**Figure 3:**
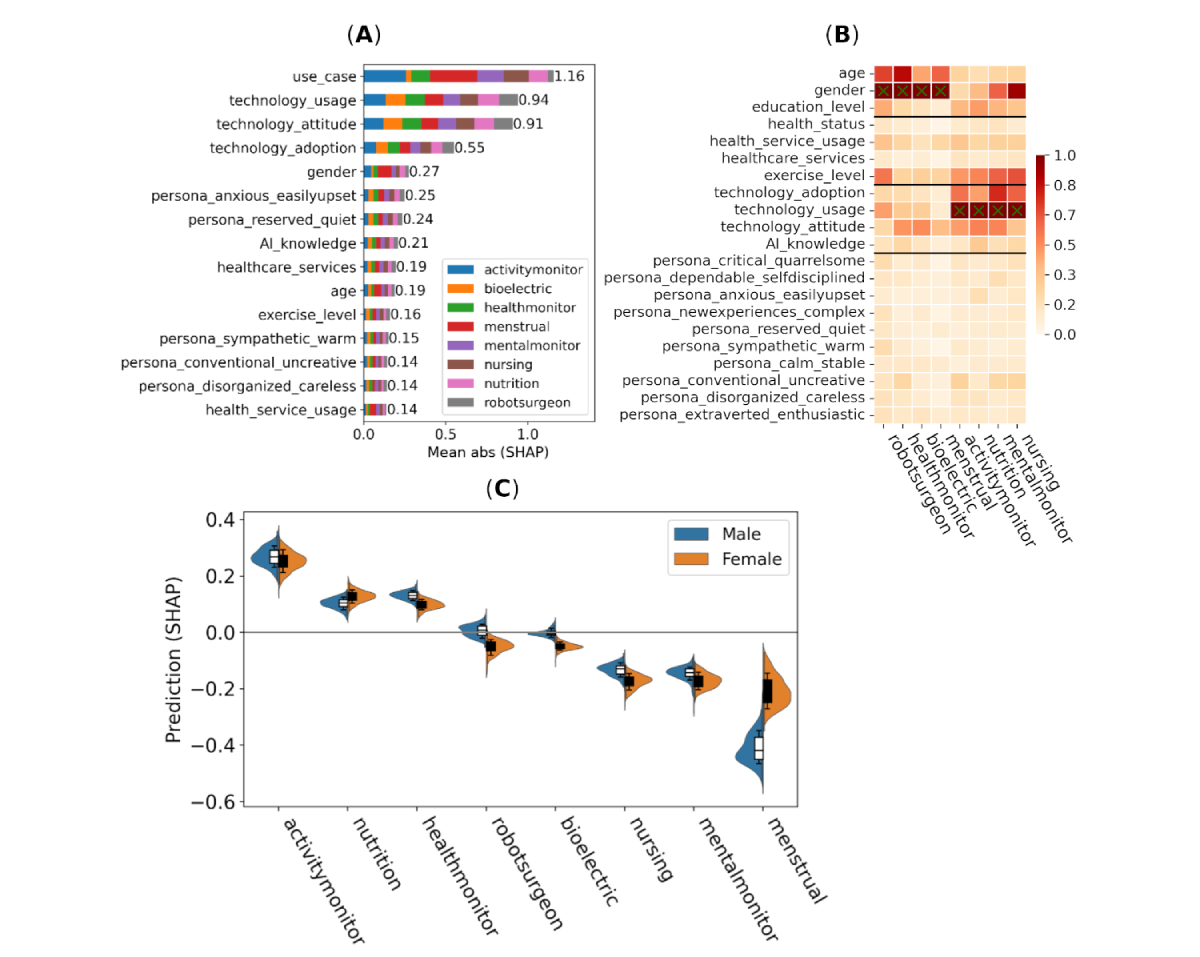
Impact of features on the intention to use the AI system if offered the chance (intention) response measured via Shapley additive explanations (SHAP) values (all data points; n=5146 data points from 1100 participants). (A) Mean absolute SHAP main effect values divided between use cases, (B) mean absolute SHAP interaction values with respect to the use case and selected variables, and (C) main effect SHAP values for each use case split between male and female (n=1050, 95.45%) participants.

In Figure 2A, we depict 1 representative sample from the data with its SHAP values computed from the trained model. After combining SHAP values of all samples (n=5146 data points with 1100 participants) by taking their absolute values and means and combining those with all 7 responses, we obtained magnitudes of impact to predictions for all 33 features. This is depicted in Figure 2B, where we have sorted features from the most important (at the top) to the least important (at the bottom) according to mean SHAP magnitudes. The order of features is the same in Figures 2A and 2B. Note that the ranking of features can differ between individual responses; for example, see Figures 3A and 6A for intention and trade-off responses, respectively.

The results indicate that the most important features overall (ranks 1 to 5) were technology_attitude, use_case, technology_adoption, gender, and technology_usage. Other important features covered education_level, age, AI_knowledge, and various personality features. Features related to the education field and work sector were negligible.

### Feature Importance for Intention to Use of Use Cases

Feature importance for intention (ie, intention to use the AI system if offered the chance) are depicted in Figure 3, including the mean of absolute SHAP values and SHAP values for individual use cases and interaction strength for the most important variables. In Figure 3B, we have sorted columns (use cases) according to the similarity of SHAP values for easier visual comparison.

**Figure 4:**
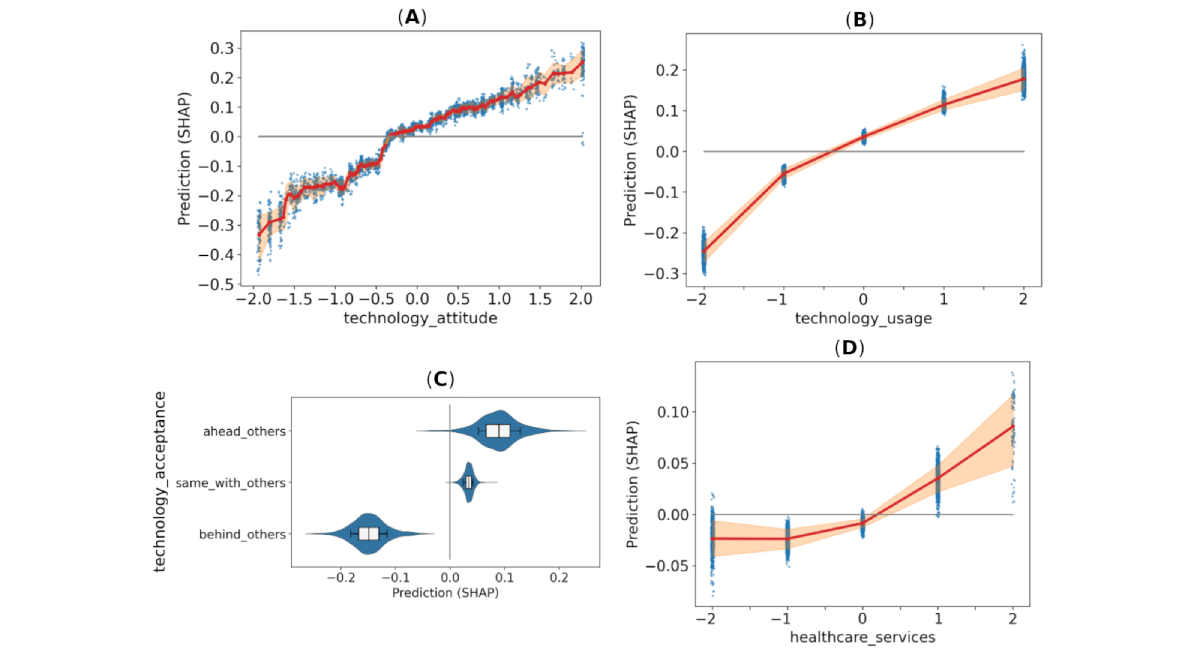
Impact of features on the intention to use the artificial intelligence (AI) system if offered the chance (Intention response) measured via Shapley additive explanation (SHAP) values (all data points; n=5146 data points from 1100 participants). The shaded and boxed regions correspond with the 75th percentile of data. (A) Technology attitude, (B) technology use, (C) technology adoption, and (D) opinion on the status of health care services.

In Figure 4, SHAP estimates are depicted for technology-related variables technology_attitude, technology_usage, and technology adoption, as well as a responders’ view of the status of current health care services (variable health care_services). The shaded regions in subplots in Figures 4A, 4B, and 4D correspond with the 75th percentile of data with the mean value at the center, both computed using a sliding-window approach, while box and whiskers in the subplot in Figure 4C correspond with the 50th and 75th percentile of data. These variables remained highly similar for trust and trade-off; hence, separate plots for those are omitted.

### Feature Importance for Trust Toward Use Cases

**Figure 5:**
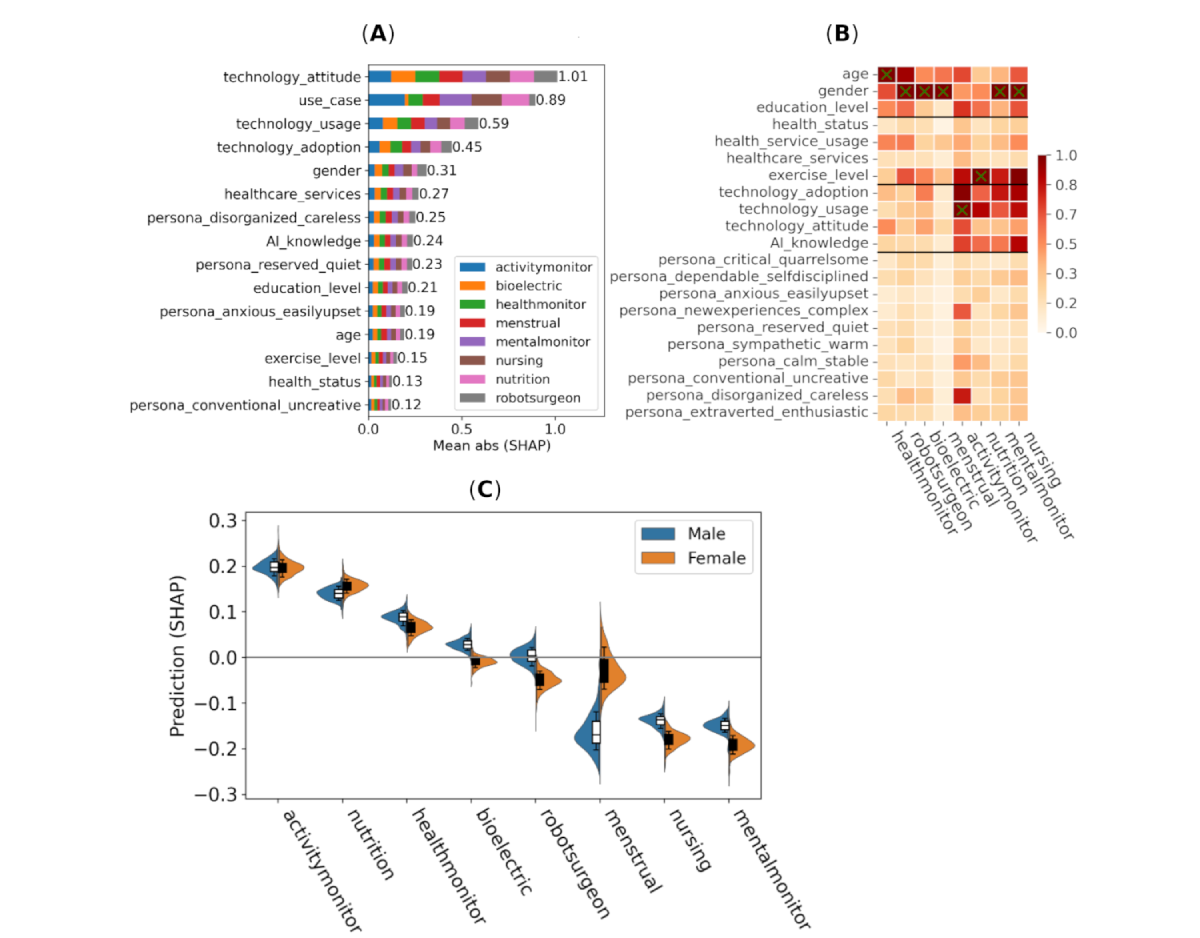
Impact of features on trust that artificial intelligence (AI) systems can make valid and accurate decisions (trust response) measured via Shapley additive explanation (SHAP) values (all data points; n=5146 data points from 1100 participants). (A) Mean absolute SHAP main effect values divided between use cases, (B) mean absolute SHAP interaction values with respect to the use case and selected variables, and (C) Main effect SHAP values for each use case split between male and female (n=1050, 95.45%) participants.

Feature importance for trust that AI systems can make valid and accurate decisions (trust) are depicted in Figure 5, including the mean of absolute SHAP values and SHAP values for individual use cases and interaction strength for the most important variables.

In distinction from Figure 3B, the importance of the exercise_level variable is elevated for activity and mental monitor, nutrition assistant, and nursing robot, as shown in Figure 5B.

### Feature Importance for the Trade-Off Willingness of Use Cases

**Figure 6:**
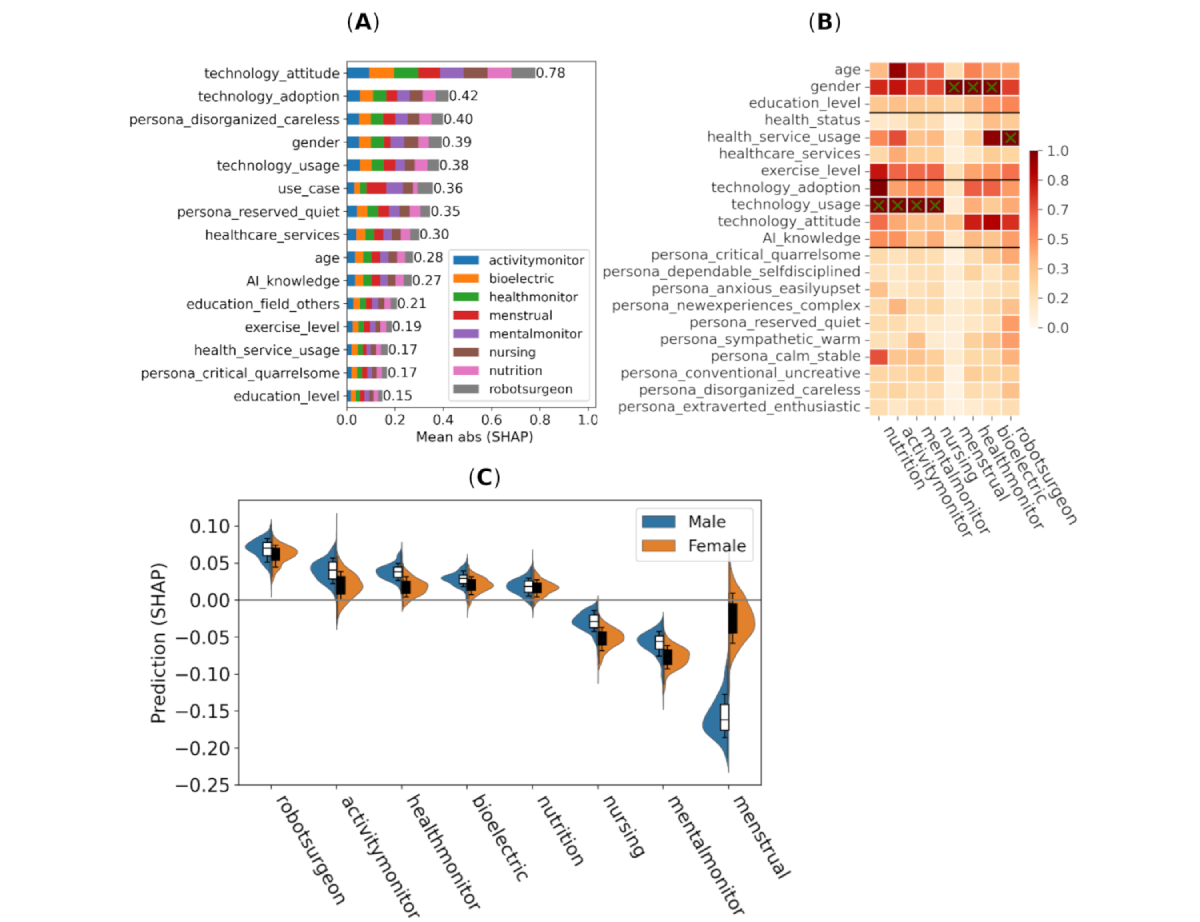
Impact of features on willingness to share personal data to improve artificial intelligence (AI) predictions (trade-off response) measured via Shapley additive explanation (SHAP) values (all data points; n=5146 data points from 1100 participants). (A) Mean absolute SHAP main effect values divided between use cases, (B) mean absolute SHAP interaction values with respect to the use case and selected variables, and (C) main effect SHAP values for each use case split between male and female (n=1050, 95.45%) participants.

Feature importance for willingness to share personal data to improve AI predictions (trade-off) is depicted in Figure 6, including the mean of absolute SHAP values and SHAP values for individual use cases and interaction strength for the most important variables.

### Impact of Age, Knowledge of AI, and Personality

**Figure 7:**
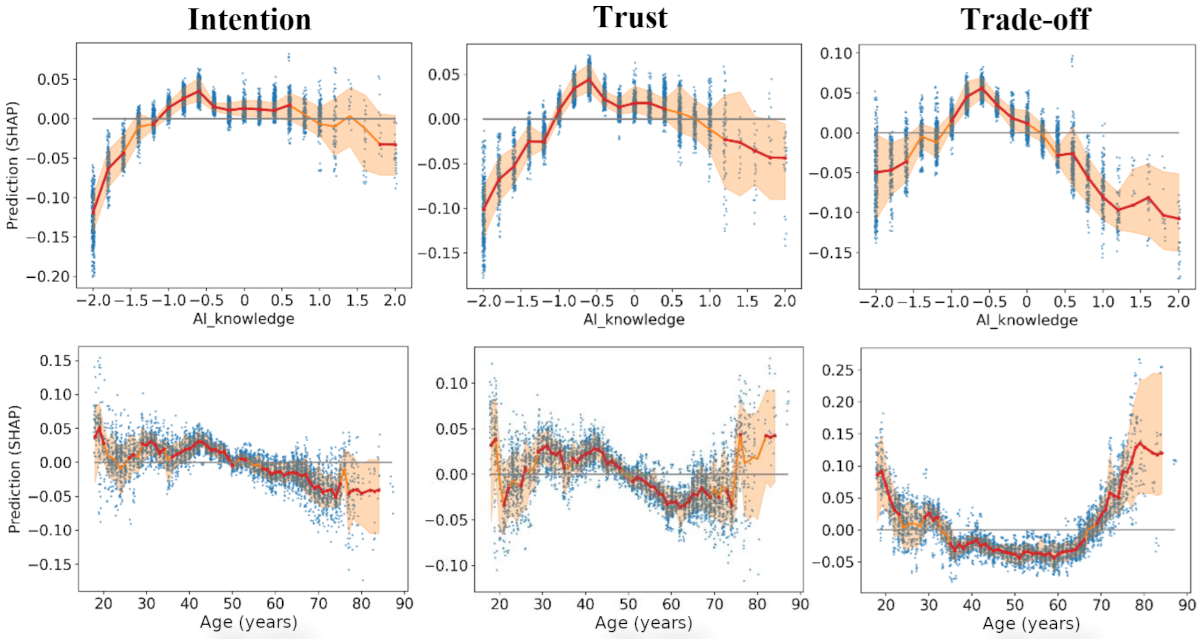
Impact of artificial intelligence (AI) knowledge and age on the intention, trust, and trade-off response measured via Shapley additive explanations (SHAP) main effect values (all data points; n=5146 data points from 1100 participants). The shaded region corresponds with the 75th percentile of data.

Finally, a side-by-side comparison of the other highly important variables, AI knowledge, and age, is depicted in Figure 7. Positive and negative values correspond with increasing and decreasing contributions to the prediction, respectively. Results indicate that those who were least familiar with AI were also less interested in using or trusting AI. Older people were generally more reserved, at least those aged 65 to 70 years. People aged >70 years were most positive toward AI when considering the trade-off measure.

**Figure 8:**
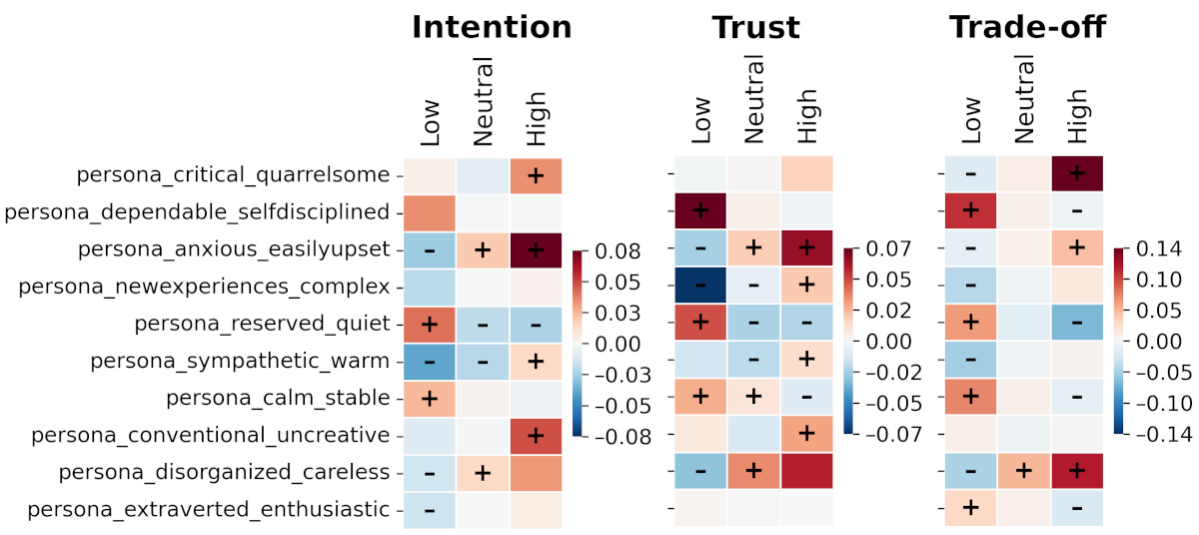
Impact of personality on intention, trust, and trade-off responses measured via Shapley additive explanations (SHAP) main effect mean values (all data points; n=5146 data points from 1100 participants). Plus (+) and minus (–) signs correspond to 75th percentile of SHAP values having positive or negative signs.

Figure 8 depicts the effect of personality traits. Each colored cell corresponds with the mean of SHAP values over low (Likert values of –3 and –2), neutral (Likert values from –1 to –1), and high (Likert values of 2 and 3) responses. Positive values correspond with increasing contributions to the prediction and vice versa. The strongest patterns by overall importance remain similar between all 3 responses, namely with the positive relationship for *disorganized and careless* and *anxious and easily upset* and negative for *reserved and quiet*.

### Nonlinear Interactions and Example Predictions

SHAP interaction matrices involving variable pairs are depicted in Figures S1 and S2 Multimedia Appendix 7 for both models. Strongest interactions involve variables age, gender, education_level, exercise_level, health care_services, health_status, technology_adoption, technology_attitude, technology_usage, and AI_knowledge. In addition to the use case, gender had strong interactions with education level, technology attitude, health status, and age. Personality had typically weak interactions with other variables, particularly for model 1. The strongest pairwise interactions are illustrated in Figures S3 (model 1) and S4 (model 2) in Multimedia Appendix 7.

We noticed that low and high levels of technology attitude had different effects depending on education level, gender, and AI knowledge. For example, low values of technology attitude in female participants can increase trust toward AI, while the effect was negative for male participants (Figure S3 in Multimedia Appendix 7). The same was noticed for high school or vocational educational backgrounds versus others (Figure S3 in Multimedia Appendix 7). The main effects showed a general positive relationship between opinion on health care services and acceptance of AI (Figure 3D); however, interactions revealed that age affects this relationship; opinions toward AI were reversed from between those aged <55 years (positive relationship) and those >55 years (negative relationship; Figure S3 in Multimedia Appendix 7). A similar type of “tipping point” effect occurred between technology attitude and AI knowledge (Figure S4 in Multimedia Appendix 7), technology attitude and gender (Figure S4 in Multimedia Appendix 7), and age and disorganized and careless personality (Figure S4 in Multimedia Appendix 7). Additional findings are listed in Multimedia Appendix 7.

Finally, in Multimedia Appendix 8, we depicted illustrative predictions for individual use cases with either very high or low predictions for trust (Figure S1 in Multimedia Appendix 8) and trade-off (Figure S2 in Multimedia Appendix 8) side by side. Predictions were dominated by technological attitude and adoption, but personality traits and gender also had a notable impact.

## Discussion

### Overview

In this study, the acceptance, trust, intention, and related views of consumers (health and medical care clients and prospective patients) toward the use of AI in the field of health care were evaluated to map the views of individuals, such as prospective patient, consumer, and user, toward AI use cases. The cases ranged from simple health-related applications to more futuristic cases of invasive AI solutions, such as surgical robotics. Using CatBoost and SHAP allowed us to find nonlinear relationships between predictors and responses. Evaluations were collected about the intention to use (intention), trust for AI (trust), model predictions (predictions), training data (data), privacy issues (privacy), willingness to trade-off information for benefits (trade-off), and manufacturer importance (manufacturer) for each use case.

### Principal Findings

Correlations between the 7 properties were statistically different from 0 (*P*<.01; Table 1) for all except intention and manufacturer and trade-off and predictions. Correlations were highest (>0.7) for intention and trust (0.808), predictions and data (0.840), data and manufacturer (0.794), predictions and manufacturer (0.752), and data and privacy (0.708). For negative correlations, the strongest was privacy and trade-off (–0.468), illustrating that people with a higher demand for privacy are not eager to compromise it for benefits. For the overall case, evaluations were analyzed for intention and trust and concerns over accessibility and cybersecurity in AI. For these, the strongest positive correlation was found for intention and trust (0.768) and a negative correlation for trust and cybersecurity (–0.591; Table S1 in Multimedia Appendix 6). The latter confirms that concern regarding security in AI is strongly associated with decreased trust in technology. For intention and trust, our results were at par with existing knowledge of the strong connection between the two [10,11,13].

For the predictive model 1 (with use cases), the single most important variable was technology attitude (rank 1), followed by related variables technology adoption (ranks 2 and 7), technology use (ranks 5 and 2), and AI knowledge (ranks 8 and 4), as depicted in Figure 2 and Figure S1 in Multimedia Appendix 7. Technology attitude was more important than the use case (rank 2). This indicates that general attitude, view, and knowledge of technology are the primary drivers of how consumers evaluate AI applications. This sets the baseline for the individual evaluation of AI that is further adjusted by particular use cases. This is in line with the previous research that showed how attitudes impact the willingness to accept technology, particularly AI in health care [18,19]. Personality had a major impact on all outputs. Although individual personality traits alone were not of the top importance, most of them (6/10, 60%) were considered more important than health status, work sector, or education field (Figure 3). In fact, the pooled importance of all 10 personality traits (a SHAP value of 1.38) came close to the pooled importance of 4 AI attitude, use, and knowledge-related variables (a SHAP value of 1.54). Thus, this study only slightly supports earlier research findings. In addition, as stated earlier, we critiqued the clarity of the earlier research findings. The findings of this study also display a positive link between attitude toward the state of health care services and trust, intention to use, and willingness to trade-off. People with positive and very positive views toward health care services were also positive toward AI (Figure 4).

With regard to trust and intention, our use cases were divided into 3 tiers of popularity (Figures 3C and 5C):

- Tier 1 (ranks 1 to 3): activity monitor, nutrition monitor, and health monitor (noninvasive)
- Tier 2 (ranks 4 to 5): robot surgeon and health monitor (bioelectric)
- Tire 3 (ranks 6 to 8): nursing robot, mental monitor, and menstrual monitor

However, predicted differences were small, around 0.5 on the numerical scale (ie, half step in the Likert scale). As expected, the gender difference was notable for the menstrual monitor (+0.1 for women) and also for the bioelectric health monitor, nursing robot, and robot surgeon (+0.02 for men). A similar finding was reported by Omrani et al [17] with men having higher levels of trust in AI than women. Looking into interaction effects (Figures 3B and 5B) between use cases and selected demographic and personality traits revealed another distinction between the following use cases:

- Group 1: robot surgeon, health monitors (noninvasive and bioelectric), and menstrual monitor
- Group 2: nursing robot, mental monitor, nutrition monitor, and activity monitor

For group 1, the most important features were gender and age. For group 2, the most important features were technology adaptation, exercise level, technology use, and AI knowledge. There was a negligible effect from health status, health service use, technology attitude, or personality traits, which was important only in setting the baseline opinion toward AI. The results for model 2 (without use cases) remained similar to those of model 1 regarding the most important features, which were technology attitude, technology use, and view on health care services (Figure S2 in Multimedia Appendix 6).

In addition to the use case, gender had notable interactions with education level, technology attitude, health status, and age, as measured via SHAP values. We noticed the following: (1) increasing technology attitude decreased trust and use intention for female participants; (2) with “ahead of others” skills in technology adoption, male participants had a higher willingness for trade-off compared to female participants; (3) among those with good health status, female participants had higher trust and use intention compared to male participants; and (4) increased age for female participants also increased trust, use intention, and willingness for trade-off, while the opposite was true for male participants (Multimedia Appendix 7).

Attitude toward technology was by far the most important factor for trade-off (0.78 in SHAP). At the same time, the use case was the sixth most important factor (0.36 in SHAP; Figure 6). The ranking of use cases was similar to above, however, with the robot surgeon now having ranked first (vs fourth and fifth earlier). This indicates that consumers acknowledge the importance of having the most accurate and detailed data about themselves available for invasive operations, such as surgery, and are willing to share sensitive health data. A robot surgeon could be preferred due to its potential accuracy, while a robot nurse would not be preferred due to the absence of social interaction and perhaps empathy with the patient. On the other hand, the same effect was not present for the mental health case (a SHAP value of –0.12 against the robot surgeon). One explanation for this could be that data related to mental health are considered more sensitive than physical data, even regarding health benefits. For interactions, a similar grouping as before was found regarding gender (group 1) and technology use (group 2) being the most important variables for each group.

For personality traits, we found that out of all 10 traits, the most important were *disorganized and careless* (rank 9) and *reserved and quiet* (rank 10 overall). This corresponds with the findings by Park and Woo [25], where extroverts were shown to demonstrate a more negative disposition toward technology than others. However, the contribution seen in this study is that the importance of individual traits varied depending on the output dimension. Covariance with outputs was mostly monotonous with respect to the Likert scale measured via the SHAP algorithm. Relationships in the 7 dimensions were positive for all except for *dependable and self-disciplined, reserved and quiet,* and *calm and stable* (Figure 8). It may be summarized that if a person has high levels of disorganized and careless, anxious and easily upset, and critical and quarrelsome traits and low levels of dependable and self-disciplined as well as reserved and quiet traits, they are likely to have higher levels of trust, intention to use, and willingness to do trade-offs for AI. Despite having strong main effects, interaction effects for personality with other variables were generally weak, particularly for model 1 (Multimedia Appendix 7). However, for model 2, some notable interactions were present for disorganized and careless, reserved and quiet, and extraverted and enthusiastic, particularly with AI knowledge. A high disorganized and careless score was associated with an increasing (increasingly positive) relationship with respect to AI knowledge toward trust and intention. The opposite relationship was present with respect to age.

We also found notable nonlinearities for certain features, highlighting the complex interactions underlying the data and the corresponding need for nonlinear modeling to explain them. For model 1, a decreasing (increasingly negative) relationship was seen toward intention, trust, and trade-off that started around the age of 40 years (Figure 7). However, people aged >70 years had a strong positive relationship for trade-off with a lesser degree of trust. This indicates more willingness toward trade-off, as one’s health deteriorates with age, particularly so for female participants (Figure S3 in Multimedia Appendix 7). This group recognizes the acute need for health and medical care, yet it has most likely considered or experienced current societal challenges in the underresourcing of the health and medical sectors. Thus, while trust is not high (ie, as seen in a forced digitalization relationship), the recognized need for technological intervention is understood. For model 2 (Figure S3 in Multimedia Appendix 6), three predictive relationships were found: (1) consumers aged <30 years had the lowest intention to use and trust AI, (2) middle-aged consumers aged 35 to 50 years had increased intention to use AI, and (3) the older consumers aged >65 years had the highest levels of trust in AI.

For AI knowledge, a strong inverted *U* shape was found, in which predictions for intention, trust, and trade-off were negative for those with the least and most knowledge about AI according to their own self-evaluations (Figure 7 and Figure S3 in Multimedia Appendix 6). This maintains a resemblance with the Dunning-Kruger effect [55], where people with the least and most knowledge in certain domains have the highest confidence or expectations for potential outcomes. In this study, the effect was inversed, whereby people with the self-evaluated least or most knowledge of AI possessed the most negative expectations. The effect was similar for both models 1 and 2, although for the latter, the dip for the high-end tail was smaller (Figure S3 in Multimedia Appendix 6). For age, the effect varied depending on the model and specific response. For trust and trade-off, we found a positive relationship for older responders (aged >65 years). For intention, young people (aged <30 years) were at the highest (Figure 7) or lowest (Figure S3 in Multimedia Appendix 6), indicating notable context dependency. Park and Woo [25] reported a curvilinear relationship between age and the functionality of the AI dimension, corresponding to a *U*-shaped dependency similar to our findings for trade-off. The effect of age remains diverse and context dependent.

Finally, for trust, intention, and trade-off responses, strong nonlinear interactions were found between age and health care services and technology attitude and AI knowledge (Figure S3 in Multimedia Appendix 7). For the former, an abrupt change could be observed in SHAP predictions for participants aged between 50 and 60 years: people aged >60 years with negative views on health care services (Likert responses <0) demonstrated a positive impact on outcomes. A similar effect of “flipping” of the views was found for AI knowledge concerning technology attitude (Figure S4 in Multimedia Appendix 7).

### Theoretical and Practical Contributions

This study contributes to existing knowledge both methodologically and data wise. Previous studies on the subject routinely used simple statistical methods, typically linear regression (eg, [17,22,23]) or structural equation modeling (eg, [7,20,25]). These can only consider limited numbers of variables and direct impacts without considering nonlinear interactions. Our study included 33 variables, considering all interactions between them, and a large sample of 1100 participants from all consumer groups. The importance of the variables was quantified using the SHAP method, which allows estimation and separation of main and interaction effects.

Attitude, exposure, use, and interest in technology were the main factors determining *baseline* readiness and interest in adopting AI tools in health care. There was a distinction between use cases according to how invasive (physically, socially, and psychologically) AI was perceived in the contexts. However, use cases had less overall importance on the population level than had been hypothesized at the beginning of the study. Regarding the relationship of perceived risk (vulnerability) between people and the AI systems, we assumed that the robot surgeon, mental health monitor, and menstrual monitor would be the most sensitive and least positive (more skeptical) application contexts for AI. Yet, age, gender, education level, and personality were proven to be relevant factors, while education and professional fields had negligible effects. Considering that most previous studies have been conducted on the younger population (average age ≤40 years [9]), our findings for the older population are relevant. Our results for interaction effects demonstrate that age, gender, education level, use, and attitude toward technology, and domain-specific variables (personal health and view of health care services) contribute to AI acceptance. For example, different combinations of age and education can impact female and male participants differently and nonlinearly.

While technological attitude was the major driving factor, the use case was associated with differences between genders and variable interactions. Hence, there was some context dependency. On the basis of literacy, one could assume that the application context within the use cases would significantly impact the ways in which participants evaluated the AI. Ries [27], for instance, proposed a context-dependent Bayesian model of trust, observing that trust is dependent on a number of factors that may either confirm or reject trust, such as conditions (ie, the quality and integrity of a system or object), other actors (host organization or security threats), or the level of risk (potential direct harm or no perceived risk). This stands to reason in perceptive studies as information driven through metaphors (ie, AI, health, type of procedure, or function) are interpreted via context, that is, their relationship to other elements within the perceived scenario [56]. Yet, similar to the recent findings of Stein et al [26], context does not seem to play as great a role in appraising technology as other factors such as personality type and age. The study by Stein et al [26] revealed that the motivation behind engagement (also reliant on personality type) held a strong relationship with attitude toward technology.

Our findings highlight the importance of considering a wide range of factors when determining the antecedents of trust and the use of AI in health care. These factors include the perception of ethical and privacy risks in AI-driven applications. The findings suggest that the health care context may have a more dominant influence on attitudes than specific use cases within health care. Regardless of the microapplication context (use context), the characteristics of AI in health care have an emotional weight. This would mean that all AI-driven applications are *equal*, if they are applied in the same field or overarching context, such as health care. Thus, the use cases can be interpreted rather as domains or sector-specific issues instead of contexts in and of themselves.

The findings related to AI knowledge and its reversed *U*-shape effect emphasize the importance of education and awareness raising of AI. In addition, it gravitates toward the fundamental findings of pragmatic approaches to ethical AI development [35,57], whereby awareness raising and education are critical. The results show that those with very low AI knowledge (novices) and experts are most critically disposed of AI regarding intention to use and trust the technology in health care settings, with novices showing the strongest negative attitudes. On the basis of the findings of the *U*-shaped effect, we conclude that novices and experts are the most critical. We expect experts to be aware of risks and precautions, whereas novices may be hesitant due to unfamiliarity with the technology. Thus, the majority are aware of risks to some extent, but these are not overestimated or underestimated.

### Limitations

This work is not without limitations. First, some use cases were still hypothetical (eg, autonomous robot surgeon and nurse), and respondents were required to imagine their experience of these systems based on partial (textual and pictorial) information. No actual user evaluations of these AI applications could be made available. Second, although the data were a representative sample of the Finnish population and consumers regarding age, gender, and education, it was still biased toward people who chose to participate in the survey and were proficient in digital technology, at least enough to respond to web-based surveys. The fact that all participants had access to computers, phones, and the internet may have skewed the sample toward a higher acceptance of technology. Third, participating in the survey was voluntary, which might cause self-selection bias. Fourth, although our fitted models were statistically valid, their predictive power for unseen testing data was generally low (*R*^2^ values of 0.105 and 0.225 for models 1 and 2). This may have resulted from multiple reasons, including the complexity of the survey, survey fatigue, use cases not being engaging, or some relevant predictor variables missing beyond those included. On the other hand, small *R*^2^ values appear common for web-based survey studies and reported by other researchers as well, for example, values of 0.08 to 0.17 [25], 0.03 to 0.14 [22], and 0.02 to 0.18 [25] for linear models without data splitting and 0.11 to 0.30 for boosting models with splitting [50].

### Conclusions

This study aimed to understand the willingness to use and trust AI by measuring overall attitudes to various factors in AI in health care in general, as well as dimensions of use case scenarios. The effect of context on individuals’ willingness to trust and accept AI in health care proved nuanced. Instead of a use case, the opinion was mainly driven by the individual’s attitude toward AI and use of technology. This carries through to the subquestion of the relationship between perceived risk and the AI application. Noninvasive and less sensitive AI applications, such as activity, nutrition, and health monitors, were considered the most trustworthy and favored, while the nursing robot and the mental health monitor were the least trustworthy and favored. However, when it came to making trade-offs for benefits, a robot surgeon was ranked highest. Mental health monitoring is perceived with more skepticism than general health monitoring and even menstrual monitoring. In addition, we found the following patterns:

1. An inverted, *U*-shaped relationship between AI knowledge and attitudes: both the least and most knowledgeable individuals (by self-evaluation) showed more negative attitudes for intention, trust, and trade-off. This maintains a resemblance to the Dunning-Kruger effect.
2. A clear personality-attitude relationship: positive attitudes toward AI in health care were associated with higher scores in certain traits (disorganized and careless and anxious and easily upset) and lower scores in others (reserved and quiet).
3. A distinct gender effect: women were more cautious than men toward AI in health care, with these differences becoming more pronounced depending on the use case, education level, technology attitude, and age.

These findings have important implications for designing health care–related AI systems. The results indicate that demographic factors influence attitudes toward AI in health care differently across various application settings. Consumer trust and intention to use AI are driven mainly by overall interest and use of technology, as well as personality traits, age, and gender. Training predictive AI models, such as those used in this study, could be used as a tool to adjust and inform on the correct implementation of other AI tools in specific situations. Such AI models could serve as decision-making tools for deploying AI and communicating the systems to clients (ie, explainable AI). Building on these implications, further research is needed into how developing interventions with AI increases trust in AI applications. This includes investigating how educational programs and hands-on experience with AI systems might influence acceptance formation. In addition, researchers should examine how increasing understanding and experiences about the benefits and limitations of AI affect the acceptance of AI and user trust in health care. Longitudinal research tracking how trust in AI evolves over time with increased awareness and exposure would provide valuable insights into health care organizations implementing AI solutions.

## Appendix

Multimedia Appendix 1 List of response variables, related references, and confirmatory factor analysis.

Multimedia Appendix 2 Copy of the complete web-based survey, including all questions.

Multimedia Appendix 3 Description of 8 artificial intelligence use cases.

Multimedia Appendix 4 List of predictor variables with descriptions and numerical mapping of ordinal responses.

Multimedia Appendix 5 Details of gradient boosting models and parameters and testing R2 values.

Multimedia Appendix 6 Predictive modeling results for the overall intention and trust (part 3 of the survey).

Multimedia Appendix 7 Pairwise interaction effects for predictive models.

Multimedia Appendix 8 Example model predictions for maximal and minimal trust and trade-off willingness.

## Abbreviations

ADM: automated decision-making
AI: artificial intelligence
PIIT: personal innovativeness in IT
SHAP: Shapley additive explanations

## Data Availability

The datasets generated and analyzed during this study are available from the corresponding author on reasonable request.
