# Supplementary material for: Trust and Acceptance Challenges in the Adoption of AI Applications in Health Care: Quantitative Survey Analysis
Source: J Med Internet Res. 2025 Mar 21;27:e65567. doi: 10.2196/65567 (PMC11971584; doi:10.2196/65567)
Supplement: Multimedia Appendix 1 [file jmir_v27i1e65567_app1.docx]

Confirmatory Factor Analysis fit parameters

*Questions and items*

For each use case (part 2 of the survey), the following 21 questions were asked using 5-point Likert scale. For each question, the corresponding variable name is listed.

1. How do the following describe your opinion on using this AI system? - I would use this AI system: **intention_1**
2. How do the following describe your opinion on using this AI system? - I think this AI system would be useful: **intention_2**
3. How do the following describe your opinion on using this AI system? - I plan on using AI for these purposes: **intention_3**
4. How do the following describe your opinion of trust about this AI system? - I trust that this AI system can make optimal decisions for my health and well-being: **trust_1**
5. How do the following describe your opinion of trust about this AI system? - I trust that this AI system is capable of making decisions for my health and well-being: **trust_2**
6. How do the following describe your opinion of trust about this AI system? - I trust that decisions made by this AI system are at least as good if not better than that of humans: **trust_3**
7. How do the following describe your opinion related to predictions made by this AI system? - I want to know how this AI system produces its results: **predictions_1**
8. How do the following describe your opinion related to predictions made by this AI system? - It’s important for me to be able to understand the processes behind results and decisions of this AI system: **predictions_2**
9. How do the following describe your opinion related to predictions made by this AI system? - Results produced by this AI system should be explained and shown in an understandable and transparent way: **predictions_3**
10. How do the following describe your opinion on training data of this AI system? - The quantity and type of data used in training this AI system should be explained to the user: **data_1**
11. How do the following describe your opinion on training data of this AI system? - The types and quantities of data used in training this AI system do not need to be made known to users: **data_2**
12. How do the following describe your opinion on training data of this AI system? - It is critical that users know how much and what kinds of data are used in training this AI system: **data_3**
13. How do the following describe your opinion related to the developer of this AI system? - The technology company developing this AI system (e.g., its size, country and familiarity) are crucial in building trust with users: **manufacturer_1**
14. How do the following describe your opinion related to the developer of this AI system? - The company, its national origins and brand reputation are not crucial information for users of this AI system: **manufacturer_2**
15. How do the following describe your opinion related to the developer of this AI system? - All users should be aware of which technology company has developed this AI system: **manufacturer_3**
16. How do the following describe your opinion on your data used by this AI system? - I don’t really care about where my data is stored, who can view it and how it is used: **privacy_1**
17. How do the following describe your opinion on your data used by this AI system? - I am sensitive towards knowing where data will be stored, who can access it, and how it will be used: **privacy_2**
18. How do the following describe your opinion on your data used by this AI system? - It is critical that I know how and where the data is stored, who has access and the ways in which it will be used: **privacy_3**
19. How do the following describe your opinion on your data used by this AI system? - I am willing to share my personal and sensitive data to improve predictions by this AI system: **tradeoff_1**
20. How do the following describe your opinion on your data used by this AI system? - I would not want to share my personal and sensitive data for the purpose of improving predictions by this AI system: **tradeoff_2**
21. How do the following describe your opinion on your data used by this AI system? - Sharing personal and sensitive data is meaningful to me when I know its improving the accuracy of predictions by this AI system: **tradeoff_3**

All questions were tailored for our survey, but were based on the literature listed below:

- Intention: (Venkatesh et al., 2003), (Esmaeilzadeh, 2020), (Choung et al., 2023), (Kuen et al., 2023) (Dhagarra et al., 2020), (Pavlou & Gefen, 2004), (Alanzi et al., 2023)
- Trust: (Vakkuri et al., 2021), (Rousi, 2022), (Cheung & To, 2017), (Pavlou & Gefen, 2004), (Nadarzynski et al., 2019), (Esmaeilzadeh, 2020)
- Predictions: (Vakkuri et al., 2021), (Rousi, 2022), (Nadarzynski et al., 2019)
- Data: (Vakkuri et al., 2021), (Rousi, 2022)
- Privacy: (Vakkuri et al., 2021), (Rousi, 2022), (Nadarzynski et al., 2019), (Esmaeilzadeh, 2020), (Dhagarra et al., 2020)
- Tradeoff: (Vakkuri et al., 2021), (Rousi, 2022), (Esmaeilzadeh, 2020)
- Manufacturer: (Vakkuri et al., 2021), (Rousi, 2022), (Esmaeilzadeh, 2020), (Dhagarra et al., 2020)

In part 3 of the survey, we asked and analyzed the following 16 questions using 5-point Likert scale. For each question, the corresponding variable name is included.

1. How do the following describe your opinion on using AI systems in healthcare and well-being? - Given the chance, I predict that I would prefer AI systems in the future: **intention_1**
2. How do the following describe your opinion on using AI systems in healthcare and well-being? - It is likely that I will use AI systems in the near future: **intention_2**
3. How do the following describe your opinion on using AI systems in healthcare and well-being? - Given the opportunity, I intend to use AI systems in the future: **intention_3**
4. How do the following describe your trust on AI applications in healthcare and well-being? - It is easy for me to trust AI applications: **trust_1**
5. How do the following describe your trust on AI applications in healthcare and well-being? - My tendency to trust AI applications is high: **trust_2**
6. How do the following describe your trust on AI applications in healthcare and well-being? - I tend to trust AI applications even though I have little knowledge: **trust_3**
7. How do the following describe your opinion on cybersecurity of AI in healthcare and well-being? - Issues with confidentiality may be experienced via the nature of AI: **cybersecurity_1**
8. How do the following describe your opinion on cybersecurity of AI in healthcare and well-being? - People will treat AI the same way they treat the internet, using it for information seeking without considering potential threats: **cybersecurity_2**
9. How do the following describe your opinion on cybersecurity of AI in healthcare and well-being? - I do not feel that the AI can responsibly guarantee confidentiality: **cybersecurity_3**
10. How do the following describe your opinion on cybersecurity of AI in healthcare and well-being? - I think these AI applications are prone to security threats: **cybersecurity_4**
11. How do the following describe your opinion on accessibility of AI in healthcare and well-being? - People of all types of abilities should be able to easily use AI: **accessibility_1**
12. How do the following describe your opinion on accessibility of AI in healthcare and well-being? - AI should be usable and accessible for everyone regardless of abilities: **accessibility_2**
13. How do the following describe your opinion on accessibility of AI in healthcare and well-being? - Accessible AI increases opportunities for both health and wellbeing practitioners and patients with disabilities: **accessibility_3**
14. How do the following describe your opinion on accessibility of AI in healthcare and well-being? - AI should be able to process input and offer output for people of diverse abilities: **accessibility_4**
15. How do the following describe your opinion on accessibility of AI in healthcare and well-being? - AI can support people with diverse needs: **accessibility_5**
16. How do the following describe your opinion on accessibility of AI in healthcare and well-being? - I feel concerned about people with special needs interacting with AI: **accessibility_6**

Intention and trust were based on the same sources as above in Part 2. For accessibility and cybersecurity, we applied (Vakkuri et al., 2021), (Rousi, 2022) and (Nadarzynski et al., 2019).

*Measurement model constructs and confirmatory factor analysis*

In order to build responses for the regression, we applied Confirmatory Factor Analysis (CFA) to build measurement model constructs. This was done independently for part 2 (use cases) and part 3 of the survey, as well as for one predictor variable from part 1 (background information) called “technology_attitude”.

**Model 1 (part 2 of the survey with use-cases):** The model with 19 items demonstrated a robust fit to the data, as evidenced by high values of both the Comparative Fit Index (CFI) and the Tucker-Lewis Index (TLI). Specifically, the CFI was 0.989, suggesting an excellent fit, a result consistent with guidelines proposed by Hu and Bentler (1999), who recommend a CFI value above 0.95 for indicating a good fit. Similarly, the TLI was 0.986, again exceeding the recommended threshold and reinforcing the model's adequacy (Hu & Bentler, 1999). In terms of reliability, the Cronbach's Alpha and Omega values ranged from 0.780 to 0.902 across different factors, indicating acceptable to good internal consistency. This is in line with the recommendations by Hair et al. (2022), who suggests that values above 0.7 are indicative of reliable constructs. Furthermore, the parameter estimates, especially the factor loadings, were statistically significant (p<0.001), underscoring the relevance of individual items in representing their respective latent constructs. These results affirmed the construct validity. Similarly, the construct for the technology orientation (4 items; technology_attitude) resulted in a valid measurement model with CFI 1.000 and TLI 0.999. Cronbach's Alpha and Omega values were 0.868 and 0.878. All values were within acceptable ranges (Byrne, 2001; Hair, J. F., Hult, G. T. M., Ringle, C. M., & Sarstedt, 2022; Hair et al., 2010).

**Model 2 (part 3 of the survey with overall opinion):** Factor constructs, and related items are listed in Table 3 of the appendix. For this CFA model, fitting parameters were CFI 1.000 and TLI 1.004. Cronbach's Alpha and Omega values ranged from 0.739 to 0.898 and the average variance extracted (avevar) from 0.537 to 0.745 across different factors, indicating an acceptable to good internal consistency. The construct for the technology orientation (4 items; technology_attitude) resulted in CFI and TLI of 1.000 and 1.002, and Cronbach's Alpha and Omega were 0.868 and 0.878. All values were within acceptable ranges (Byrne, 2001; Hair et al., 2010). In the predictive modeling, we, however, did not include Cybersecurity and Accessibility as prediction performance for these targets was low (below 0.20 in explained variance R^2^).

Fit parameters of response and predictor factor models, including estimates, standard errors and z-values are listed in Table S1 (model 1) and S2 (model 2).

**Table S1.** Fitting parameters for constructs involving responses (total 7) and predictors (total 1) for part 2 of the survey involving use-cases. Estimate P(>|z|)=0 for all items was 0.

| **Type** | **Construct** | **Item** | **Estimate** | **Std.Err** | **z-value** |
| --- | --- | --- | --- | --- | --- |
| Response | Intention | Q1_intention_1 | 1.057 | 0.012 | 91.795 |
|  |  | Q2_intention_2 | 0.849 | 0.01 | 85.97 |
|  |  | Q3_intention_3 | 0.987 | 0.011 | 90.783 |
|  | Trust | Q4_trust_1 | 0.918 | 0.01 | 90.724 |
|  |  | Q5_trust_2 | 0.908 | 0.01 | 90.588 |
|  |  | Q6_trust_3 | 0.829 | 0.009 | 87.359 |
|  | Predictions | Q7_predictions_1 | 0.728 | 0.01 | 73.745 |
|  |  | Q8_predictions_2 | 0.761 | 0.01 | 75.296 |
|  |  | Q9_predictions_3 | 0.647 | 0.009 | 73.173 |
|  | Data | Q10_data_1 | 0.733 | 0.009 | 81.138 |
|  |  | Q11_data_2* | 0.71 | 0.009 | 79.736 |
|  |  | Q12_data_3 | 0.751 | 0.009 | 82.005 |
|  | Privacy | Q16_privacy_1* | 0.669 | 0.009 | 71.008 |
|  |  | Q17_privacy_2 | 0.696 | 0.009 | 73.594 |
|  |  | Q18_privacy_3 | 0.704 | 0.009 | 75.106 |
|  | Trade-off | Q19_trafeoff_1 | 1.016 | 0.016 | 64.119 |
|  |  | Q20_tradeoff_2* | 0.813 | 0.013 | 64.119 |
|  | Manufacturer | Q13_manufacturer_1 | 0.626 | 0.011 | 58.541 |
|  |  | Q15_manufacturer_3 | 0.738 | 0.013 | 58.541 |
| Predictor | technology_attitude | attitude_1 | 0.992 | 0.015 | 66.571 |
|  |  | attitude_2 | 0.88 | 0.013 | 65.492 |
|  |  | attitude_3 | 0.594 | 0.011 | 55.658 |
|  |  | attitude_4 | 0.952 | 0.014 | 66.43 |

* = inverted

**Table S2.** Fitting parameters for constructs involving responses (total 4) for part 3 of the survey related to overall views of AI in healthcare and wellbeing. Estimate P(>|z|)=0 for all items was 0.

| **Type** | **Construct** | **Item** | **Estimate** | **Std.Err** | **z-value** |
| --- | --- | --- | --- | --- | --- |
| Response | Intention | intention_3 | 0.883 | 0.026 | 34.622 |
|  |  | intention_2 | 0.798 | 0.024 | 33.054 |
|  |  | intention_1 | 0.887 | 0.025 | 35.286 |
|  | Trust | trust_2 | 0.862 | 0.022 | 38.366 |
|  |  | trust_1 | 0.914 | 0.023 | 39.748 |
|  |  | trust_3 | 0.813 | 0.021 | 37.923 |
|  | Accessibility | accessibility_2 | 0.621 | 0.031 | 19.747 |
|  |  | accessibility_1 | 0.631 | 0.032 | 19.857 |
|  |  | accessibility_4 | 0.487 | 0.026 | 18.42 |
|  | Cybersecurity | cybersecurity_1 | 0.559 | 0.027 | 20.68 |
|  |  | cybersecurity_4 | 0.687 | 0.033 | 20.68 |

*Descriptive analysis for model 1*

After fitting the factor measurement models, the means of the factors were extracted by computing weighted averages of item values over the original data. The results are depicted in Figure S1 for use cases (part 2 of the survey), including the means and 75% percentile coverage. Predictions, Data, Privacy, and Manufacturer were considered important with means corresponding with “agree”. Intention, Trust and Tradeoff were considered neutral with means close to zero. The largest differences for use cases were found for Intention and Trust, while differences were generally small for others.


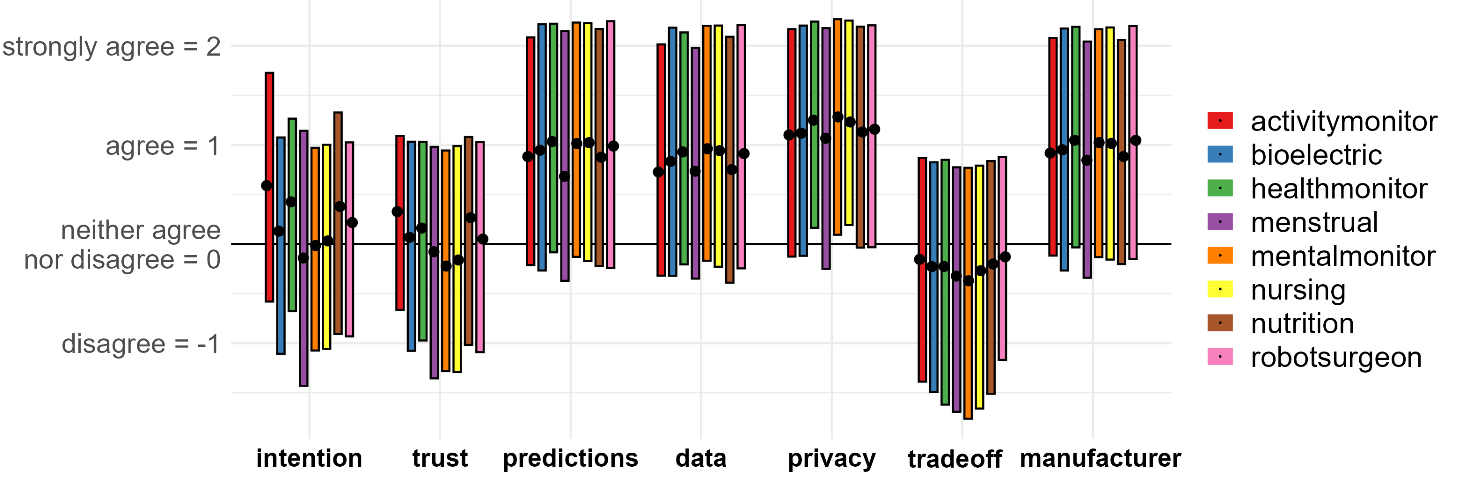


**Figure S1.** Response factors for use-cases (part 2 of the survey; n=5146, 1100 subjects) with their means (black dots) and 75% percentiles (bars) around means. The corresponding Likert-scale is shown on the y-axis.

References

Alanzi, T., Alsalem, A. A., Alzahrani, H., Almudaymigh, N., Alessa, A., Mulla, R., AlQahtani, L., Bajonaid, R., Alharthi, A., Alnahdi, O., & Alanzi, N. (2023). AI-Powered Mental Health Virtual Assistants Acceptance: An Empirical Study on Influencing Factors Among Generations X, Y, and Z. *Cureus*, *15*(11). https://doi.org/10.7759/cureus.49486

Byrne, B. (2001). Structural Equation Modeling: Perspectives on the Present and the Future. *International Journal of Testing*, *1*(3), 327–334. https://doi.org/10.1207/s15327574ijt013&4_11

Cheung, M. F. Y., & To, W. M. (2017). The influence of the propensity to trust on mobile users’ attitudes toward in-app advertisements: An extension of the theory of planned behavior. *Computers in Human Behavior*, *76*, 102–111. https://doi.org/10.1016/j.chb.2017.07.011

Choung, H., David, P., & Ross, A. (2023). Trust in AI and Its Role in the Acceptance of AI Technologies. *International Journal of Human–Computer Interaction*, *39*(9), 1727–1739. https://doi.org/10.1080/10447318.2022.2050543

Dhagarra, D., Goswami, M., & Kumar, G. (2020). Impact of Trust and Privacy Concerns on Technology Acceptance in Healthcare: An Indian Perspective. *International Journal of Medical Informatics*, *141*, 104164. https://doi.org/10.1016/j.ijmedinf.2020.104164

Esmaeilzadeh, P. (2020). Use of AI-based tools for healthcare purposes: A survey study from consumers’ perspectives. *BMC Medical Informatics and Decision Making*, *20*(1), 1–19. https://doi.org/10.1186/s12911-020-01191-1

Hair, J. F., Hult, G. T. M., Ringle, C. M., & Sarstedt, M. (2022). *A Primer on Partial Least Squares Structural Equation Modeling (PLS-SEM)* (3rd ed.). SAGE Publications, Inc.

Hair, J. F., Black, W. C., Babin, B. J., & Anderson, R. E. (2010). *Multivariate Data Analysis* (7th ed.). Prentice Hall.

Kuen, L., Westmattelmann, D., Bruckes, M., & Schewe, G. (2023). Who earns trust in online environments? A meta-analysis of trust in technology and trust in provider for technology acceptance. *Electronic Markets*, *33*(1). https://doi.org/10.1007/s12525-023-00672-1

Nadarzynski, T., Miles, O., Cowie, A., & Ridge, D. (2019). Acceptability of artificial intelligence (AI)-led chatbot services in healthcare: A mixed-methods study. *Digital Health*, *5*, 1–12. https://doi.org/10.1177/2055207619871808

Pavlou, P. A., & Gefen, D. (2004). Building effective online marketplaces with institution-based trust. *Information Systems Research*, *15*(1). https://doi.org/10.1287/isre.1040.0015

Rousi, R. (2022). With Clear Intention—An Ethical Responsibility Model for Robot Governance. *Frontiers in Computer Science*, *4*. https://doi.org/10.3389/fcomp.2022.852528

Vakkuri, V., Kemell, K. K., Jantunen, M., Halme, E., & Abrahamsson, P. (2021). ECCOLA — A method for implementing ethically aligned AI systems. *Journal of Systems and Software*, *182*. https://doi.org/10.1016/j.jss.2021.111067

Venkatesh, Morris, Davis, & Davis. (2003). User Acceptance of Information Technology: Toward a Unified View. *MIS Quarterly*, *27*(3), 425. https://doi.org/10.2307/30036540
