# Supplementary material for: Trust and Acceptance Challenges in the Adoption of AI Applications in Health Care: Quantitative Survey Analysis
Source: J Med Internet Res. 2025 Mar 21;27:e65567. doi: 10.2196/65567 (PMC11971584; doi:10.2196/65567)
Supplement: Multimedia Appendix 2 [file jmir_v27i1e65567_app2.docx]

AI in healthcare and well-being

## Welcome!

In this survey we study your experiences and opinions about artificial intelligence (AI) usage in the field of health and well-being. The survey contains three parts:

1. Background questions
2. Scenarios of AI usage (total 5)
3. General questions about AI

We define AI as a machine or a software that is able to perform activities that are typically associated with human intelligence, such as reasoning, learning, planning and creativity. Unlike traditional machines and computer programs, AI can create its own rules and principles to make decisions without humans.

Completing this survey takes around 15-20 min. The survey is a part of the [AI Forum](https://www.aiforum.fi/) [(https://www.aiforum.fi/)](https://www.aiforum.fi/) research project.

Responsible researcher and contact person: Senior researcher Janne Kauttonen (janne.kauttonen@haaga-helia.fi), Haaga-Helia University of Applied Sciences

There are 98 questions in this survey.

# Background_questions_page1

1

Part 1

We begin with demographic information. Questions marked with red stars are mandatory and necessary for proceeding.


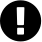


2 What is your age in years? *

Only numbers may be entered in this field.

Please write your answer here:


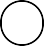

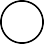

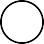

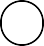


3 What is your gender identity? *

Please choose **only one** of the following:

Male Female Nonbinary

I would rather not say


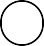

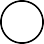

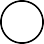

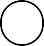

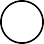


4 What is your highest level of education? *

Please choose **only one** of the following:

Elementary school

High school or vocational certificate Undergraduate degree (e.g., bachelor) Graduate degree (e.g., master's) Postgraduate degree (licentiate or doctorate)

5 Your field or fields of education? You may choose multiple. *

Please choose **all** that apply:

Education sector Humanities and arts Social sector

Commerce, Administration and Law Natural sciences

Information processing and telecommunications (ICT) Engineering

Agriculture and forestry sector Health and wellness sector Service sector

Other (none of above)

6 Your current field or fields of work? You may choose multiple. *

Please choose **all** that apply:

Education sector Humanities and arts Social sector

Commerce, Administration and Law Natural sciences

Information processing and telecommunications (ICT) Engineering

Agriculture and forestry sector Health and wellness sector Service sector

Other (none of above)

## 7 How do the following statements apply to your personality? *

Please choose the appropriate response for each item:

|  | **strongly disagre** | **disagre emodera** | **disagree e a telliyttle** | **neither agree nor**  **disagree** | **agree a little** | **agree modera** | **agree tsetlyrongly** |
| --- | --- | --- | --- | --- | --- | --- | --- |
| **critical, quarrelsome** | 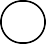 | 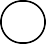 | 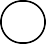 | 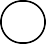 | 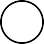 | 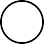 | 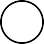 |
| **dependable, self- disciplined** | 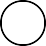 | 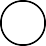 | 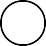 | 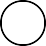 | 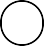 | 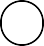 | 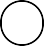 |
| **anxious, easily upset** | 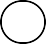 | 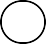 | 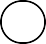 | 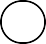 | 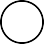 | 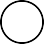 | 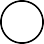 |
| **open to new experiences, complex** | 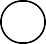 | 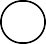 | 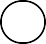 | 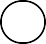 | 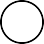 | 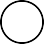 | 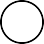 |
| **reserved, quiet** | 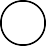 | 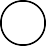 | 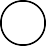 | 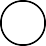 | 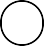 | 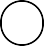 | 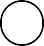 |
| **sympathetic, warm** | 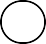 | 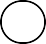 | 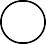 | 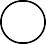 | 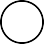 | 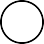 | 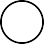 |
| **calm, emotionally stable** | 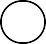 | 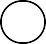 | 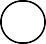 | 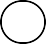 | 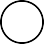 | 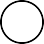 | 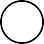 |
| **conventional, uncreative** | 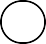 | 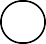 | 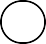 | 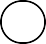 | 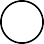 | 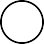 | 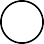 |
| **disorganized, careless** | 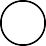 | 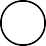 | 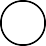 | 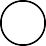 | 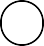 | 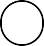 | 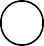 |
| **extraverted, enthusiastic** | 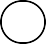 | 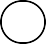 | 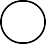 | 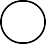 | 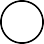 | 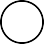 | 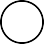 |

Consider the pair of traits and choose the best option, even if one characteristic applies more strongly than the other.

# Background_questions_page2


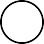

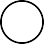

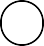

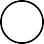

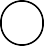


8 How do you consider your IT skills compared to others? *

Please choose **only one** of the following:

Very poor Poor Average Good Very good

Consider the usage of different software and hardware products (e.g., computers, applications, smartphones)


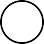

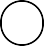

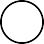


9 When it comes to adopting and using new technology, what best describes you? *

Please choose **only one** of the following:

Falling behind others, late adopter Keeping up with others

Ahead of others, early adopter

Consider any new technology related to both hardware and software products

10 How do the following describe your attitude towards new technologies? *

Please choose the appropriate response for each item:

|  | **strongly disagree** | **disagree** | **neither agree nor**  **disagree** | **agree** | **strongly agree** |
| --- | --- | --- | --- | --- | --- |
| **I usually keep an eye on emerging technology products** | 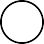 | 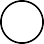 | 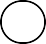 | 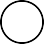 | 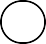 |
| **I always try out new technology products earlier compared to others** | 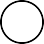 | 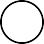 | 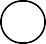 | 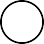 | 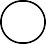 |
| **In general, I am willing to accept new technology things** | 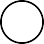 | 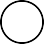 |  |  |  |
| **If I heard about a new technology product, I would look for ways to operate it** |  |  |  |  |  |

## 11 How do the following describe your knowledge and experience of AI? *

Please choose the appropriate response for each item:

|  | **strongly disagree** | **disagree** | **neither agree nor**  **disagree** | **agree** | **strongly agree** |
| --- | --- | --- | --- | --- | --- |
| **I know the difference between narrow, general and strong AI** |  |  |  |  |  |
| **I know what over- learning and under- learning means in training AI** |  |  |  |  |  |
| **I know difference between supervised and unsupervised learning** |  |  |  |  |  |
| **I have solid knowledge of the working principles of AI** |  |  |  |  |  |
| **I have experience in using AI applications** |  |  |  |  |  |

12 How do you feel about your overall health status in the past 12 months? *

Please choose **only one** of the following:

Very bad Bad

Not good or bad Good

Very good

13 How often have you used health services past 12 months? *

Please choose **only one** of the following:

Weekly or more

A few times per month Approximately monthly A few times or not at all

Include all visits to healthcare professionals, such as medical doctors, nurses, dentists and therapists

14 How do you feel about the current state of healthcare services? *

Please choose **only one** of the following:

Very unsatisfied Unsatisfied

Not satisfied or unsatisfied Satisfied

Very satisfied

Consider overall availability, cost and quality, including both public and private sector.

15 How much do you exercise and physically strain yourself in your free time? *

Please choose **only one** of the following:

I regularly practice strenuous sports several times a week in a competitive spirit (e.g., gym, running, orienteering, skiing, swimming, ball games in a competitive spirit)

I exercise several hours a week (e.g. gym, running, jogging, skiing, gymnastics, swimming, ball games)

I exercise lightly several hours a week (e.g. walking, fishing and hunting, gardening) I don't move much or strain myself physically

16 How frequently do you use technology to monitor your health and well-being? *

Please choose **only one** of the following:

Always Often Sometimes Seldom Never

Including any smart (sports) watches, smart rings, mobile apps and websites that collect data and give recommendations and feedback related to physical activity, nutrition, sleep and/or mental well-being

# scenario_welcome

17

Part 2

In the following pages, we describe various AI systems in healthcare and well-being. Note that these applications are not related to any specific products or services you might know or use, but correspond to a broader array of applications and systems available now and near future.

Please read the descriptions carefully and then answer all questions.

18 5

scenario1_activity_monitoring

19

## 20

**Activity monitoring AI system to support healthy lifestyle**

illustration

### Description:

This AI system aims to promote a healthy and active lifestyle by monitoring and analyzing physical activity and well-being. The system can measure the status of the body using a combination of a smartwatch and/or ring with wearable sensors embedded in clothes.

The system can help in optimizing daily physical activities and exercises by creating

individual workout plans and suggesting healthy options. The AI system constantly learns and improves from data and feedback. The AI system collects and analyzes the following data:

Heart rate and stroke volume Blood oxygenation and pressure Electrical activity of the muscles Respiration rhythm

Body temperature and perspiration Location data

Now please answer questions related to this AI system.

Only answer this question if the following conditions are met:

[eq1](https://haagahelia.limesurvey.net/questionAdministration/view/surveyid/566237/gid/25/qid/1353) [(/questionAdministration/view/surveyid/566237/gid/25/qid/1353)](https://haagahelia.limesurvey.net/questionAdministration/view/surveyid/566237/gid/25/qid/1353) LT [gcount](https://haagahelia.limesurvey.net/questionAdministration/view/surveyid/566237/gid/24/qid/1362) [(/questionAdministration/view/surveyid/566237/gid/24/qid/1362)](https://haagahelia.limesurvey.net/questionAdministration/view/surveyid/566237/gid/24/qid/1362)

21 How do the following describe your opinion on using this AI system? *

Only answer this question if the following conditions are met:

[eq1](https://haagahelia.limesurvey.net/questionAdministration/view/surveyid/566237/gid/25/qid/1353) [(/questionAdministration/view/surveyid/566237/gid/25/qid/1353)](https://haagahelia.limesurvey.net/questionAdministration/view/surveyid/566237/gid/25/qid/1353) LT [gcount](https://haagahelia.limesurvey.net/questionAdministration/view/surveyid/566237/gid/24/qid/1362) [(/questionAdministration/view/surveyid/566237/gid/24/qid/1362)](https://haagahelia.limesurvey.net/questionAdministration/view/surveyid/566237/gid/24/qid/1362)

Please choose the appropriate response for each item:

|  | **strongly disagree** | **disagree** | **neither agree nor**  **disagree** | **agree** | **strongly agree** |
| --- | --- | --- | --- | --- | --- |
| **I would use this AI system** |  |  |  |  |  |
| **I think this AI system would be useful** |  |  |  |  |  |
| **I plan on using AI for these purposes** |  |  |  |  |  |

## 22 How do the following describe your opinion of trust about this AI system? *

Only answer this question if the following conditions are met:

[eq1](https://haagahelia.limesurvey.net/questionAdministration/view/surveyid/566237/gid/25/qid/1353) [(/questionAdministration/view/surveyid/566237/gid/25/qid/1353)](https://haagahelia.limesurvey.net/questionAdministration/view/surveyid/566237/gid/25/qid/1353) LT [gcount](https://haagahelia.limesurvey.net/questionAdministration/view/surveyid/566237/gid/24/qid/1362) [(/questionAdministration/view/surveyid/566237/gid/24/qid/1362)](https://haagahelia.limesurvey.net/questionAdministration/view/surveyid/566237/gid/24/qid/1362)

Please choose the appropriate response for each item:

|  | **strongly disagree** | **disagree** | **neither agree nor**  **disagree** | **agree** | **strongly agree** |
| --- | --- | --- | --- | --- | --- |
| **I trust that this AI system can make**  **optimal decisions for my health and well- being** |  |  |  |  |  |
| **I trust that this AI**  **system is capable of making decisions for my health and well- being** |  |  |  |  |  |
| **I trust that decisions made by this AI**  **system are at least as good if not better than that of humans** |  |  |  |  |  |

## 23 How do the following describe your opinion related to predictions made by this AI system? *

Only answer this question if the following conditions are met:

[eq1](https://haagahelia.limesurvey.net/questionAdministration/view/surveyid/566237/gid/25/qid/1353) [(/questionAdministration/view/surveyid/566237/gid/25/qid/1353)](https://haagahelia.limesurvey.net/questionAdministration/view/surveyid/566237/gid/25/qid/1353) LT [gcount](https://haagahelia.limesurvey.net/questionAdministration/view/surveyid/566237/gid/24/qid/1362) [(/questionAdministration/view/surveyid/566237/gid/24/qid/1362)](https://haagahelia.limesurvey.net/questionAdministration/view/surveyid/566237/gid/24/qid/1362)

Please choose the appropriate response for each item:

|  | **strongly disagree** | **disagree** | **neither agree nor**  **disagree** | **agree** | **strongly agree** |
| --- | --- | --- | --- | --- | --- |
| **I want to know how this AI system produces its results** |  |  |  |  |  |
| **It’s important for me to be able to understand the processes behind results and decisions of this AI system** |  |  |  |  |  |
| **Results produced by this AI system should be explained and shown in an understandable and transparent way** |  |  |  |  |  |

## 24 How do the following describe your opinion on training data of this AI system? *

Only answer this question if the following conditions are met:

[eq1](https://haagahelia.limesurvey.net/questionAdministration/view/surveyid/566237/gid/25/qid/1353) [(/questionAdministration/view/surveyid/566237/gid/25/qid/1353)](https://haagahelia.limesurvey.net/questionAdministration/view/surveyid/566237/gid/25/qid/1353) LT [gcount](https://haagahelia.limesurvey.net/questionAdministration/view/surveyid/566237/gid/24/qid/1362) [(/questionAdministration/view/surveyid/566237/gid/24/qid/1362)](https://haagahelia.limesurvey.net/questionAdministration/view/surveyid/566237/gid/24/qid/1362)

Please choose the appropriate response for each item:

|  | **strongly disagree** | **disagree** | **neither agree nor**  **disagree** | **agree** | **strongly agree** |
| --- | --- | --- | --- | --- | --- |
| **The quantity and type of data used in training this AI system should be explained to the user** |  |  |  |  |  |
| **The types and quantities of data used in training this AI**  **system do not need to be made known to users** |  |  |  |  |  |
| **It is critical that users know how much and what kinds of data are used in training this AI system** |  |  |  |  |  |

Consider the data that was used in developing and training this AI system

## 25 How do the following describe your opinion related to the developer of this AI system? *

Only answer this question if the following conditions are met:

[eq1](https://haagahelia.limesurvey.net/questionAdministration/view/surveyid/566237/gid/25/qid/1353) [(/questionAdministration/view/surveyid/566237/gid/25/qid/1353)](https://haagahelia.limesurvey.net/questionAdministration/view/surveyid/566237/gid/25/qid/1353) LT [gcount](https://haagahelia.limesurvey.net/questionAdministration/view/surveyid/566237/gid/24/qid/1362) [(/questionAdministration/view/surveyid/566237/gid/24/qid/1362)](https://haagahelia.limesurvey.net/questionAdministration/view/surveyid/566237/gid/24/qid/1362)

Please choose the appropriate response for each item:

|  | **strongly disagree** | **disagree** | **neither agree nor**  **disagree** | **agree** | **strongly agree** |
| --- | --- | --- | --- | --- | --- |
| **The technology company developing this AI system (e.g., its size, country and familiarity) are crucial in building trust with users** |  |  |  |  |  |
| **The company, its national origins and brand reputation are not crucial information for users of this AI**  **system** |  |  |  |  |  |
| **All users should be aware of which technology company has developed this AI system** |  |  |  |  |  |

Consider the company or organization that is developing and selling this AI system

## 26 How do the following describe your opinion on your data used by this AI system? *

Only answer this question if the following conditions are met:

[eq1](https://haagahelia.limesurvey.net/questionAdministration/view/surveyid/566237/gid/25/qid/1353) [(/questionAdministration/view/surveyid/566237/gid/25/qid/1353)](https://haagahelia.limesurvey.net/questionAdministration/view/surveyid/566237/gid/25/qid/1353) LT [gcount](https://haagahelia.limesurvey.net/questionAdministration/view/surveyid/566237/gid/24/qid/1362) [(/questionAdministration/view/surveyid/566237/gid/24/qid/1362)](https://haagahelia.limesurvey.net/questionAdministration/view/surveyid/566237/gid/24/qid/1362)

Please choose the appropriate response for each item:

|  | **strongly disagree** | **disagree** | **neither agree nor**  **disagree** | **agree** | **strongly agree** |
| --- | --- | --- | --- | --- | --- |
| **I don’t really care about where my data is stored, who can view it and how it is used** |  |  |  |  |  |
| **I am sensitive towards knowing where data will be stored, who can access it, and how it will be used** |  |  |  |  |  |
| **It is critical that I know how and where the data is stored, who has access and the ways in which it will be used** |  |  |  |  |  |
| **I am willing to share my personal and sensitive data to improve predictions by this AI system** |  |  |  |  |  |
| **I would not want to share my personal and sensitive data for the purpose of improving predictions by this AI system** |  |  |  |  |  |

|  | **strongly disagree** | **disagree** | **neither agree nor**  **disagree** | **agree** | **strongly agree** |
| --- | --- | --- | --- | --- | --- |
| **Sharing personal and sensitive data is meaningful to me when I know its improving the accuracy of predictions by this AI system** |  |  |  |  |  |

Consider the data collected **from you**

27 What problems and ethical issues related to this AI system can you think of?

Only answer this question if the following conditions are met:

[eq1](https://haagahelia.limesurvey.net/questionAdministration/view/surveyid/566237/gid/25/qid/1353) [(/questionAdministration/view/surveyid/566237/gid/25/qid/1353)](https://haagahelia.limesurvey.net/questionAdministration/view/surveyid/566237/gid/25/qid/1353) LT [gcount](https://haagahelia.limesurvey.net/questionAdministration/view/surveyid/566237/gid/24/qid/1362) [(/questionAdministration/view/surveyid/566237/gid/24/qid/1362)](https://haagahelia.limesurvey.net/questionAdministration/view/surveyid/566237/gid/24/qid/1362)

Please write your answer here:

Please write you response in the field below (optional)

# scenario2_menstrual_monitoring

28

29

**Menstrual cycle monitoring and prediction AI system**

illustration

### Description:

The menstrual cycle monitoring and predicting AI system is designed to help track and predict the status of the menstrual cycle. This AI system can provide accurate predictions of when the next period is due, most likely ovulation time, and report any changes in the menstrual cycle that may require attention from healthcare services. It can be used for example to plan for pregnancy, track fertility and monitor hormonal levels. The AI system constantly learns from data and feedback. The AI system collects and analyzes the following data:

Menstrual cycle history

Symptoms such as cramps, bloating, headaches, mood changes Body temperature

Hormonal birth control use Physical and sexual activity

Status of diet, stress and medications

**Note:** You can answer on your own behalf or from the perspective of your (female) partner. You can also skip this scenario and move to the next one by leaving all responses empty.

Only answer this question if the following conditions are met:

[eq2](https://haagahelia.limesurvey.net/questionAdministration/view/surveyid/566237/gid/26/qid/1354) [(/questionAdministration/view/surveyid/566237/gid/26/qid/1354)](https://haagahelia.limesurvey.net/questionAdministration/view/surveyid/566237/gid/26/qid/1354) LT [gcount](https://haagahelia.limesurvey.net/questionAdministration/view/surveyid/566237/gid/24/qid/1362) [(/questionAdministration/view/surveyid/566237/gid/24/qid/1362)](https://haagahelia.limesurvey.net/questionAdministration/view/surveyid/566237/gid/24/qid/1362)

30 How do the following describe your opinion on using this AI system?

Only answer this question if the following conditions are met:

[eq2](https://haagahelia.limesurvey.net/questionAdministration/view/surveyid/566237/gid/26/qid/1354) [(/questionAdministration/view/surveyid/566237/gid/26/qid/1354)](https://haagahelia.limesurvey.net/questionAdministration/view/surveyid/566237/gid/26/qid/1354) LT [gcount](https://haagahelia.limesurvey.net/questionAdministration/view/surveyid/566237/gid/24/qid/1362) [(/questionAdministration/view/surveyid/566237/gid/24/qid/1362)](https://haagahelia.limesurvey.net/questionAdministration/view/surveyid/566237/gid/24/qid/1362)

Please choose the appropriate response for each item:

|  | **strongly disagree** | **disagree** | **neither agree nor**  **disagree** | **agree** | **strongly agree** |
| --- | --- | --- | --- | --- | --- |
| **I would use this AI system** |  |  |  |  |  |
| **I think this AI system would be useful** |  |  |  |  |  |
| **I plan on using AI for these purposes** |  |  |  |  |  |

## 31 How do the following describe your opinion of trust about this AI system?

Only answer this question if the following conditions are met:

[eq2](https://haagahelia.limesurvey.net/questionAdministration/view/surveyid/566237/gid/26/qid/1354) [(/questionAdministration/view/surveyid/566237/gid/26/qid/1354)](https://haagahelia.limesurvey.net/questionAdministration/view/surveyid/566237/gid/26/qid/1354) LT [gcount](https://haagahelia.limesurvey.net/questionAdministration/view/surveyid/566237/gid/24/qid/1362) [(/questionAdministration/view/surveyid/566237/gid/24/qid/1362)](https://haagahelia.limesurvey.net/questionAdministration/view/surveyid/566237/gid/24/qid/1362)

Please choose the appropriate response for each item:

|  | **strongly disagree** | **disagree** | **neither agree nor**  **disagree** | **agree** | **strongly agree** |
| --- | --- | --- | --- | --- | --- |
| **I trust that this AI system can make**  **optimal decisions for my health and well- being** |  |  |  |  |  |
| **I trust that this AI**  **system is capable of making decisions for my health and well- being** |  |  |  |  |  |
| **I trust that decisions made by this AI**  **system are at least as good if not better than that of humans** |  |  |  |  |  |

## 32 How do the following describe your opinion related to predictions made by this AI system?

Only answer this question if the following conditions are met:

[eq2](https://haagahelia.limesurvey.net/questionAdministration/view/surveyid/566237/gid/26/qid/1354) [(/questionAdministration/view/surveyid/566237/gid/26/qid/1354)](https://haagahelia.limesurvey.net/questionAdministration/view/surveyid/566237/gid/26/qid/1354) LT [gcount](https://haagahelia.limesurvey.net/questionAdministration/view/surveyid/566237/gid/24/qid/1362) [(/questionAdministration/view/surveyid/566237/gid/24/qid/1362)](https://haagahelia.limesurvey.net/questionAdministration/view/surveyid/566237/gid/24/qid/1362)

Please choose the appropriate response for each item:

|  | **strongly disagree** | **disagree** | **neither agree nor**  **disagree** | **agree** | **strongly agree** |
| --- | --- | --- | --- | --- | --- |
| **I want to know how this AI system produces its results** |  |  |  |  |  |
| **It’s important for me to be able to understand the processes behind results and decisions of this AI system** |  |  |  |  |  |
| **Results produced by this AI system should be explained and shown in an understandable and transparent way** |  |  |  |  |  |

## 33 How do the following describe your opinion on training data of this AI system?

Only answer this question if the following conditions are met:

[eq2](https://haagahelia.limesurvey.net/questionAdministration/view/surveyid/566237/gid/26/qid/1354) [(/questionAdministration/view/surveyid/566237/gid/26/qid/1354)](https://haagahelia.limesurvey.net/questionAdministration/view/surveyid/566237/gid/26/qid/1354) LT [gcount](https://haagahelia.limesurvey.net/questionAdministration/view/surveyid/566237/gid/24/qid/1362) [(/questionAdministration/view/surveyid/566237/gid/24/qid/1362)](https://haagahelia.limesurvey.net/questionAdministration/view/surveyid/566237/gid/24/qid/1362)

Please choose the appropriate response for each item:

|  | **strongly disagree** | **disagree** | **neither agree nor**  **disagree** | **agree** | **strongly agree** |
| --- | --- | --- | --- | --- | --- |
| **The quantity and type of data used in training this AI system should be explained to the user** |  |  |  |  |  |
| **The types and quantities of data used in training this AI**  **system do not need to be made known to users** |  |  |  |  |  |
| **It is critical that users know how much and what kinds of data are used in training this AI system** |  |  |  |  |  |

Consider the data that was used in developing and training this AI system

## 34 How do the following describe your opinion related to the developer of this AI system?

Only answer this question if the following conditions are met:

[eq2](https://haagahelia.limesurvey.net/questionAdministration/view/surveyid/566237/gid/26/qid/1354) [(/questionAdministration/view/surveyid/566237/gid/26/qid/1354)](https://haagahelia.limesurvey.net/questionAdministration/view/surveyid/566237/gid/26/qid/1354) LT [gcount](https://haagahelia.limesurvey.net/questionAdministration/view/surveyid/566237/gid/24/qid/1362) [(/questionAdministration/view/surveyid/566237/gid/24/qid/1362)](https://haagahelia.limesurvey.net/questionAdministration/view/surveyid/566237/gid/24/qid/1362)

Please choose the appropriate response for each item:

|  | **strongly disagree** | **disagree** | **neither agree nor**  **disagree** | **agree** | **strongly agree** |
| --- | --- | --- | --- | --- | --- |
| **The technology company developing this AI system (e.g., its size, country and familiarity) are crucial in building trust with users** |  |  |  |  |  |
| **The company, its national origins and brand reputation are not crucial information for users of this AI**  **system** |  |  |  |  |  |
| **All users should be aware of which technology company has developed this AI system** |  |  |  |  |  |

Consider the company or organization that is developing and selling this AI system

## 35 How do the following describe your opinion on your data used by this AI system?

Only answer this question if the following conditions are met:

[eq2](https://haagahelia.limesurvey.net/questionAdministration/view/surveyid/566237/gid/26/qid/1354) [(/questionAdministration/view/surveyid/566237/gid/26/qid/1354)](https://haagahelia.limesurvey.net/questionAdministration/view/surveyid/566237/gid/26/qid/1354) LT [gcount](https://haagahelia.limesurvey.net/questionAdministration/view/surveyid/566237/gid/24/qid/1362) [(/questionAdministration/view/surveyid/566237/gid/24/qid/1362)](https://haagahelia.limesurvey.net/questionAdministration/view/surveyid/566237/gid/24/qid/1362)

Please choose the appropriate response for each item:

|  | **strongly disagree** | **disagree** | **neither agree nor**  **disagree** | **agree** | **strongly agree** |
| --- | --- | --- | --- | --- | --- |
| **I don’t really care about where my data is stored, who can view it and how it is used** |  |  |  |  |  |
| **I am sensitive towards knowing where data will be stored, who can access it, and how it will be used** |  |  |  |  |  |
| **It is critical that I know how and where the data is stored, who has access and the ways in which it will be used** |  |  |  |  |  |
| **I am willing to share my personal and sensitive data to improve predictions by this AI system** |  |  |  |  |  |
| **I would not want to share my personal and sensitive data for the purpose of improving predictions by this AI system** |  |  |  |  |  |

|  | **strongly disagree** | **disagree** | **neither agree nor**  **disagree** | **agree** | **strongly agree** |
| --- | --- | --- | --- | --- | --- |
| **Sharing personal and sensitive data is meaningful to me when I know its improving the accuracy of predictions by this AI system** |  |  |  |  |  |

Consider the data collected **from you**

36 What problems and ethical issues related to this AI system can you think of?

Only answer this question if the following conditions are met:

[eq2](https://haagahelia.limesurvey.net/questionAdministration/view/surveyid/566237/gid/26/qid/1354) [(/questionAdministration/view/surveyid/566237/gid/26/qid/1354)](https://haagahelia.limesurvey.net/questionAdministration/view/surveyid/566237/gid/26/qid/1354) LT [gcount](https://haagahelia.limesurvey.net/questionAdministration/view/surveyid/566237/gid/24/qid/1362) [(/questionAdministration/view/surveyid/566237/gid/24/qid/1362)](https://haagahelia.limesurvey.net/questionAdministration/view/surveyid/566237/gid/24/qid/1362)

Please write your answer here:

Please write you response in the field below (optional)

# scenario3_robotic_surgeon

37

38

**AI-controlled robotic surgeon**

illustration

### Description:

The robotic surgeon is an AI-controlled system that can perform various types of surgeries for human patients. The machine is equipped with advanced sensors and mechanical instruments designed to perform surgical procedures autonomously and accurately without human intervention. The system uses real-time data from various imaging technologies (e.g., laser scanning, MRI, CT, and ultrasound) to create an

accurate 3D map of the patient's anatomy. The AI analyzes data and controls trajectories, speeds, and forces of instruments to predict and perform the operation optimally. The AI system collects and analyzes the following data:

Full health records, medical history and genetic data

Imaging data including laser scanning, MRI, CT and ultrasound Accurate, real-time sensor data to track patient physiology

Now please answer questions related to this AI system.

Only answer this question if the following conditions are met:

[eq3](https://haagahelia.limesurvey.net/questionAdministration/view/surveyid/566237/gid/27/qid/1355) [(/questionAdministration/view/surveyid/566237/gid/27/qid/1355)](https://haagahelia.limesurvey.net/questionAdministration/view/surveyid/566237/gid/27/qid/1355) LT [gcount](https://haagahelia.limesurvey.net/questionAdministration/view/surveyid/566237/gid/24/qid/1362) [(/questionAdministration/view/surveyid/566237/gid/24/qid/1362)](https://haagahelia.limesurvey.net/questionAdministration/view/surveyid/566237/gid/24/qid/1362)

39 How do the following describe your opinion on using this AI system? *

Only answer this question if the following conditions are met:

[eq3](https://haagahelia.limesurvey.net/questionAdministration/view/surveyid/566237/gid/27/qid/1355) [(/questionAdministration/view/surveyid/566237/gid/27/qid/1355)](https://haagahelia.limesurvey.net/questionAdministration/view/surveyid/566237/gid/27/qid/1355) LT [gcount](https://haagahelia.limesurvey.net/questionAdministration/view/surveyid/566237/gid/24/qid/1362) [(/questionAdministration/view/surveyid/566237/gid/24/qid/1362)](https://haagahelia.limesurvey.net/questionAdministration/view/surveyid/566237/gid/24/qid/1362)

Please choose the appropriate response for each item:

|  | **strongly disagree** | **disagree** | **neither agree nor**  **disagree** | **agree** | **strongly agree** |
| --- | --- | --- | --- | --- | --- |
| **I would use this AI system** |  |  |  |  |  |
| **I think this AI system would be useful** |  |  |  |  |  |
| **I plan on using AI for these purposes** |  |  |  |  |  |

## 40 How do the following describe your opinion of trust about this AI system? *

Only answer this question if the following conditions are met:

[eq3](https://haagahelia.limesurvey.net/questionAdministration/view/surveyid/566237/gid/27/qid/1355) [(/questionAdministration/view/surveyid/566237/gid/27/qid/1355)](https://haagahelia.limesurvey.net/questionAdministration/view/surveyid/566237/gid/27/qid/1355) LT [gcount](https://haagahelia.limesurvey.net/questionAdministration/view/surveyid/566237/gid/24/qid/1362) [(/questionAdministration/view/surveyid/566237/gid/24/qid/1362)](https://haagahelia.limesurvey.net/questionAdministration/view/surveyid/566237/gid/24/qid/1362)

Please choose the appropriate response for each item:

|  | **strongly disagree** | **disagree** | **neither agree nor**  **disagree** | **agree** | **strongly agree** |
| --- | --- | --- | --- | --- | --- |
| **I trust that this AI system can make**  **optimal decisions for my health and well- being** |  |  |  |  |  |
| **I trust that this AI**  **system is capable of making decisions for my health and well- being** |  |  |  |  |  |
| **I trust that decisions made by this AI**  **system are at least as good if not better than that of humans** |  |  |  |  |  |

## 41 How do the following describe your opinion related to predictions made by this AI system? *

Only answer this question if the following conditions are met:

[eq3](https://haagahelia.limesurvey.net/questionAdministration/view/surveyid/566237/gid/27/qid/1355) [(/questionAdministration/view/surveyid/566237/gid/27/qid/1355)](https://haagahelia.limesurvey.net/questionAdministration/view/surveyid/566237/gid/27/qid/1355) LT [gcount](https://haagahelia.limesurvey.net/questionAdministration/view/surveyid/566237/gid/24/qid/1362) [(/questionAdministration/view/surveyid/566237/gid/24/qid/1362)](https://haagahelia.limesurvey.net/questionAdministration/view/surveyid/566237/gid/24/qid/1362)

Please choose the appropriate response for each item:

|  | **strongly disagree** | **disagree** | **neither agree nor**  **disagree** | **agree** | **strongly agree** |
| --- | --- | --- | --- | --- | --- |
| **I want to know how this AI system produces its results** |  |  |  |  |  |
| **It’s important for me to be able to understand the processes behind results and decisions of this AI system** |  |  |  |  |  |
| **Results produced by this AI system should be explained and shown in an understandable and transparent way** |  |  |  |  |  |

## 42 How do the following describe your opinion on training data of this AI system? *

Only answer this question if the following conditions are met:

[eq3](https://haagahelia.limesurvey.net/questionAdministration/view/surveyid/566237/gid/27/qid/1355) [(/questionAdministration/view/surveyid/566237/gid/27/qid/1355)](https://haagahelia.limesurvey.net/questionAdministration/view/surveyid/566237/gid/27/qid/1355) LT [gcount](https://haagahelia.limesurvey.net/questionAdministration/view/surveyid/566237/gid/24/qid/1362) [(/questionAdministration/view/surveyid/566237/gid/24/qid/1362)](https://haagahelia.limesurvey.net/questionAdministration/view/surveyid/566237/gid/24/qid/1362)

Please choose the appropriate response for each item:

|  | **strongly disagree** | **disagree** | **neither agree nor**  **disagree** | **agree** | **strongly agree** |
| --- | --- | --- | --- | --- | --- |
| **The quantity and type of data used in training this AI system should be explained to the user** |  |  |  |  |  |
| **The types and quantities of data used in training this AI**  **system do not need to be made known to users** |  |  |  |  |  |
| **It is critical that users know how much and what kinds of data are used in training this AI system** |  |  |  |  |  |

Consider the data that was used in developing and training this AI system

## 43 How do the following describe your opinion related to the developer of this AI system? *

Only answer this question if the following conditions are met:

[eq3](https://haagahelia.limesurvey.net/questionAdministration/view/surveyid/566237/gid/27/qid/1355) [(/questionAdministration/view/surveyid/566237/gid/27/qid/1355)](https://haagahelia.limesurvey.net/questionAdministration/view/surveyid/566237/gid/27/qid/1355) LT [gcount](https://haagahelia.limesurvey.net/questionAdministration/view/surveyid/566237/gid/24/qid/1362) [(/questionAdministration/view/surveyid/566237/gid/24/qid/1362)](https://haagahelia.limesurvey.net/questionAdministration/view/surveyid/566237/gid/24/qid/1362)

Please choose the appropriate response for each item:

|  | **strongly disagree** | **disagree** | **neither agree nor**  **disagree** | **agree** | **strongly agree** |
| --- | --- | --- | --- | --- | --- |
| **The technology company developing this AI system (e.g., its size, country and familiarity) are crucial in building trust with users** |  |  |  |  |  |
| **The company, its national origins and brand reputation are not crucial information for users of this AI**  **system** |  |  |  |  |  |
| **All users should be aware of which technology company has developed this AI system** |  |  |  |  |  |

Consider the company or organization that is developing and selling this AI system

## 44 How do the following describe your opinion on your data used by this AI system? *

Only answer this question if the following conditions are met:

[eq3](https://haagahelia.limesurvey.net/questionAdministration/view/surveyid/566237/gid/27/qid/1355) [(/questionAdministration/view/surveyid/566237/gid/27/qid/1355)](https://haagahelia.limesurvey.net/questionAdministration/view/surveyid/566237/gid/27/qid/1355) LT [gcount](https://haagahelia.limesurvey.net/questionAdministration/view/surveyid/566237/gid/24/qid/1362) [(/questionAdministration/view/surveyid/566237/gid/24/qid/1362)](https://haagahelia.limesurvey.net/questionAdministration/view/surveyid/566237/gid/24/qid/1362)

Please choose the appropriate response for each item:

|  | **strongly disagree** | **disagree** | **neither agree nor**  **disagree** | **agree** | **strongly agree** |
| --- | --- | --- | --- | --- | --- |
| **I don’t really care about where my data is stored, who can view it and how it is used** |  |  |  |  |  |
| **I am sensitive towards knowing where data will be stored, who can access it, and how it will be used** |  |  |  |  |  |
| **It is critical that I know how and where the data is stored, who has access and the ways in which it will be used** |  |  |  |  |  |
| **I am willing to share my personal and sensitive data to improve predictions by this AI system** |  |  |  |  |  |
| **I would not want to share my personal and sensitive data for the purpose of improving predictions by this AI system** |  |  |  |  |  |

|  | **strongly disagree** | **disagree** | **neither agree nor**  **disagree** | **agree** | **strongly agree** |
| --- | --- | --- | --- | --- | --- |
| **Sharing personal and sensitive data is meaningful to me when I know its improving the accuracy of predictions by this AI system** |  |  |  |  |  |

Consider the data collected **from you**

45 What problems and ethical issues related to this AI system can you think of?

Only answer this question if the following conditions are met:

[eq3](https://haagahelia.limesurvey.net/questionAdministration/view/surveyid/566237/gid/27/qid/1355) [(/questionAdministration/view/surveyid/566237/gid/27/qid/1355)](https://haagahelia.limesurvey.net/questionAdministration/view/surveyid/566237/gid/27/qid/1355) LT [gcount](https://haagahelia.limesurvey.net/questionAdministration/view/surveyid/566237/gid/24/qid/1362) [(/questionAdministration/view/surveyid/566237/gid/24/qid/1362)](https://haagahelia.limesurvey.net/questionAdministration/view/surveyid/566237/gid/24/qid/1362)

Please write your answer here:

Please write you response in the field below (optional)

# scenario4_nutrition_optimization

46

47

**Nutrition monitoring and planning AI system**

illustration

**Description:**

This AI system aims to promote a healthy diet by monitoring and analyzing consumed meals and activities. The system analyzes all meals, calorie consumption, physical activity, rest and taste priorities to create an individualized and optimized meal plan.

Personalized diets are based on predicted blood glucose responses and energy consumption. The AI system constantly learns from data and feedback. The AI system collects and analyzes the following data:

Physical activity including sports, rest and sleep Information on consumed nutrients, meals and drinks

Body state and composition, including weight and fat percentage Medical conditions, medications and food allergies

Now please answer questions related to this AI system.

Only answer this question if the following conditions are met:

[eq4](https://haagahelia.limesurvey.net/questionAdministration/view/surveyid/566237/gid/28/qid/1356) [(/questionAdministration/view/surveyid/566237/gid/28/qid/1356)](https://haagahelia.limesurvey.net/questionAdministration/view/surveyid/566237/gid/28/qid/1356) LT [gcount](https://haagahelia.limesurvey.net/questionAdministration/view/surveyid/566237/gid/24/qid/1362) [(/questionAdministration/view/surveyid/566237/gid/24/qid/1362)](https://haagahelia.limesurvey.net/questionAdministration/view/surveyid/566237/gid/24/qid/1362)

48 How do the following describe your opinion on using this AI system? *

Only answer this question if the following conditions are met:

[eq4](https://haagahelia.limesurvey.net/questionAdministration/view/surveyid/566237/gid/28/qid/1356) [(/questionAdministration/view/surveyid/566237/gid/28/qid/1356)](https://haagahelia.limesurvey.net/questionAdministration/view/surveyid/566237/gid/28/qid/1356) LT [gcount](https://haagahelia.limesurvey.net/questionAdministration/view/surveyid/566237/gid/24/qid/1362) [(/questionAdministration/view/surveyid/566237/gid/24/qid/1362)](https://haagahelia.limesurvey.net/questionAdministration/view/surveyid/566237/gid/24/qid/1362)

Please choose the appropriate response for each item:

|  | **strongly disagree** | **disagree** | **neither agree nor**  **disagree** | **agree** | **strongly agree** |
| --- | --- | --- | --- | --- | --- |
| **I would use this AI system** |  |  |  |  |  |
| **I think this AI system would be useful** |  |  |  |  |  |
| **I plan on using AI for these purposes** |  |  |  |  |  |

## 49 How do the following describe your opinion of trust about this AI system? *

Only answer this question if the following conditions are met:

[eq4](https://haagahelia.limesurvey.net/questionAdministration/view/surveyid/566237/gid/28/qid/1356) [(/questionAdministration/view/surveyid/566237/gid/28/qid/1356)](https://haagahelia.limesurvey.net/questionAdministration/view/surveyid/566237/gid/28/qid/1356) LT [gcount](https://haagahelia.limesurvey.net/questionAdministration/view/surveyid/566237/gid/24/qid/1362) [(/questionAdministration/view/surveyid/566237/gid/24/qid/1362)](https://haagahelia.limesurvey.net/questionAdministration/view/surveyid/566237/gid/24/qid/1362)

Please choose the appropriate response for each item:

|  | **strongly disagree** | **disagree** | **neither agree nor**  **disagree** | **agree** | **strongly agree** |
| --- | --- | --- | --- | --- | --- |
| **I trust that this AI system can make**  **optimal decisions for my health and well- being** |  |  |  |  |  |
| **I trust that this AI**  **system is capable of making decisions for my health and well- being** |  |  |  |  |  |
| **I trust that decisions made by this AI**  **system are at least as good if not better than that of humans** |  |  |  |  |  |

## 50 How do the following describe your opinion related to predictions made by this AI system? *

Only answer this question if the following conditions are met:

[eq4](https://haagahelia.limesurvey.net/questionAdministration/view/surveyid/566237/gid/28/qid/1356) [(/questionAdministration/view/surveyid/566237/gid/28/qid/1356)](https://haagahelia.limesurvey.net/questionAdministration/view/surveyid/566237/gid/28/qid/1356) LT [gcount](https://haagahelia.limesurvey.net/questionAdministration/view/surveyid/566237/gid/24/qid/1362) [(/questionAdministration/view/surveyid/566237/gid/24/qid/1362)](https://haagahelia.limesurvey.net/questionAdministration/view/surveyid/566237/gid/24/qid/1362)

Please choose the appropriate response for each item:

|  | **strongly disagree** | **disagree** | **neither agree nor**  **disagree** | **agree** | **strongly agree** |
| --- | --- | --- | --- | --- | --- |
| **I want to know how this AI system produces its results** |  |  |  |  |  |
| **It’s important for me to be able to understand the processes behind results and decisions of this AI system** |  |  |  |  |  |
| **Results produced by this AI system should be explained and shown in an understandable and transparent way** |  |  |  |  |  |

## 51 How do the following describe your opinion on training data of this AI system? *

Only answer this question if the following conditions are met:

[eq4](https://haagahelia.limesurvey.net/questionAdministration/view/surveyid/566237/gid/28/qid/1356) [(/questionAdministration/view/surveyid/566237/gid/28/qid/1356)](https://haagahelia.limesurvey.net/questionAdministration/view/surveyid/566237/gid/28/qid/1356) LT [gcount](https://haagahelia.limesurvey.net/questionAdministration/view/surveyid/566237/gid/24/qid/1362) [(/questionAdministration/view/surveyid/566237/gid/24/qid/1362)](https://haagahelia.limesurvey.net/questionAdministration/view/surveyid/566237/gid/24/qid/1362)

Please choose the appropriate response for each item:

|  | **strongly disagree** | **disagree** | **neither agree nor**  **disagree** | **agree** | **strongly agree** |
| --- | --- | --- | --- | --- | --- |
| **The quantity and type of data used in training this AI system should be explained to the user** |  |  |  |  |  |
| **The types and quantities of data used in training this AI**  **system do not need to be made known to users** |  |  |  |  |  |
| **It is critical that users know how much and what kinds of data are used in training this AI system** |  |  |  |  |  |

Consider the data that was used in developing and training this AI system

## 52 How do the following describe your opinion related to the developer of this AI system? *

Only answer this question if the following conditions are met:

[eq4](https://haagahelia.limesurvey.net/questionAdministration/view/surveyid/566237/gid/28/qid/1356) [(/questionAdministration/view/surveyid/566237/gid/28/qid/1356)](https://haagahelia.limesurvey.net/questionAdministration/view/surveyid/566237/gid/28/qid/1356) LT [gcount](https://haagahelia.limesurvey.net/questionAdministration/view/surveyid/566237/gid/24/qid/1362) [(/questionAdministration/view/surveyid/566237/gid/24/qid/1362)](https://haagahelia.limesurvey.net/questionAdministration/view/surveyid/566237/gid/24/qid/1362)

Please choose the appropriate response for each item:

|  | **strongly disagree** | **disagree** | **neither agree nor**  **disagree** | **agree** | **strongly agree** |
| --- | --- | --- | --- | --- | --- |
| **The technology company developing this AI system (e.g., its size, country and familiarity) are crucial in building trust with users** |  |  |  |  |  |
| **The company, its national origins and brand reputation are not crucial information for users of this AI**  **system** |  |  |  |  |  |
| **All users should be aware of which technology company has developed this AI system** |  |  |  |  |  |

Consider the company or organization that is developing and selling this AI system

## 53 How do the following describe your opinion on your data used by this AI system? *

Only answer this question if the following conditions are met:

[eq4](https://haagahelia.limesurvey.net/questionAdministration/view/surveyid/566237/gid/28/qid/1356) [(/questionAdministration/view/surveyid/566237/gid/28/qid/1356)](https://haagahelia.limesurvey.net/questionAdministration/view/surveyid/566237/gid/28/qid/1356) LT [gcount](https://haagahelia.limesurvey.net/questionAdministration/view/surveyid/566237/gid/24/qid/1362) [(/questionAdministration/view/surveyid/566237/gid/24/qid/1362)](https://haagahelia.limesurvey.net/questionAdministration/view/surveyid/566237/gid/24/qid/1362)

Please choose the appropriate response for each item:

|  | **strongly disagree** | **disagree** | **neither agree nor**  **disagree** | **agree** | **strongly agree** |
| --- | --- | --- | --- | --- | --- |
| **I don’t really care about where my data is stored, who can view it and how it is used** |  |  |  |  |  |
| **I am sensitive towards knowing where data will be stored, who can access it, and how it will be used** |  |  |  |  |  |
| **It is critical that I know how and where the data is stored, who has access and the ways in which it will be used** |  |  |  |  |  |
| **I am willing to share my personal and sensitive data to improve predictions by this AI system** |  |  |  |  |  |
| **I would not want to share my personal and sensitive data for the purpose of improving predictions by this AI system** |  |  |  |  |  |

|  | **strongly disagree** | **disagree** | **neither agree nor**  **disagree** | **agree** | **strongly agree** |
| --- | --- | --- | --- | --- | --- |
| **Sharing personal and sensitive data is meaningful to me when I know its improving the accuracy of predictions by this AI system** |  |  |  |  |  |

Consider the data collected **from you**

54 What problems and ethical issues related to this AI system can you think of?

Only answer this question if the following conditions are met:

[eq4](https://haagahelia.limesurvey.net/questionAdministration/view/surveyid/566237/gid/28/qid/1356) [(/questionAdministration/view/surveyid/566237/gid/28/qid/1356)](https://haagahelia.limesurvey.net/questionAdministration/view/surveyid/566237/gid/28/qid/1356) LT [gcount](https://haagahelia.limesurvey.net/questionAdministration/view/surveyid/566237/gid/24/qid/1362) [(/questionAdministration/view/surveyid/566237/gid/24/qid/1362)](https://haagahelia.limesurvey.net/questionAdministration/view/surveyid/566237/gid/24/qid/1362)

Please write your answer here:

Please write you response in the field below (optional)

# scenario5_health_monitoring

55

56

**Real-time health monitoring, analysis and prediction AI system**

illustration

### Description:

This AI system monitors personal physical health and helps with any medical issues and treatments. The system can detect and predict any changes in personal health and provide recommendations on how one can improve and maintain their health, or whether there is a need to seek medical help. The system leverages your personal and medical information and takes advantage of your genetic profile to make predictions and recommendations. The AI system constantly learns from data and feedback. The AI system collects and analyzes the following data:

Physical health history, such as any issues with health and treatments Basic medical information, such as medication and illnesses

Physical activity information Genetic data

Basic demographic information

Now please answer questions related to this AI system.

Only answer this question if the following conditions are met:

[eq5](https://haagahelia.limesurvey.net/questionAdministration/view/surveyid/566237/gid/29/qid/1357) [(/questionAdministration/view/surveyid/566237/gid/29/qid/1357)](https://haagahelia.limesurvey.net/questionAdministration/view/surveyid/566237/gid/29/qid/1357) LT [gcount](https://haagahelia.limesurvey.net/questionAdministration/view/surveyid/566237/gid/24/qid/1362) [(/questionAdministration/view/surveyid/566237/gid/24/qid/1362)](https://haagahelia.limesurvey.net/questionAdministration/view/surveyid/566237/gid/24/qid/1362)

57 How do the following describe your opinion on using this AI system? *

Only answer this question if the following conditions are met:

[eq5](https://haagahelia.limesurvey.net/questionAdministration/view/surveyid/566237/gid/29/qid/1357) [(/questionAdministration/view/surveyid/566237/gid/29/qid/1357)](https://haagahelia.limesurvey.net/questionAdministration/view/surveyid/566237/gid/29/qid/1357) LT [gcount](https://haagahelia.limesurvey.net/questionAdministration/view/surveyid/566237/gid/24/qid/1362) [(/questionAdministration/view/surveyid/566237/gid/24/qid/1362)](https://haagahelia.limesurvey.net/questionAdministration/view/surveyid/566237/gid/24/qid/1362)

Please choose the appropriate response for each item:

|  | **strongly disagree** | **disagree** | **neither agree nor**  **disagree** | **agree** | **strongly agree** |
| --- | --- | --- | --- | --- | --- |
| **I would use this AI system** |  |  |  |  |  |
| **I think this AI system would be useful** |  |  |  |  |  |
| **I plan on using AI for these purposes** |  |  |  |  |  |

## 58 How do the following describe your opinion of trust about this AI system? *

Only answer this question if the following conditions are met:

[eq5](https://haagahelia.limesurvey.net/questionAdministration/view/surveyid/566237/gid/29/qid/1357) [(/questionAdministration/view/surveyid/566237/gid/29/qid/1357)](https://haagahelia.limesurvey.net/questionAdministration/view/surveyid/566237/gid/29/qid/1357) LT [gcount](https://haagahelia.limesurvey.net/questionAdministration/view/surveyid/566237/gid/24/qid/1362) [(/questionAdministration/view/surveyid/566237/gid/24/qid/1362)](https://haagahelia.limesurvey.net/questionAdministration/view/surveyid/566237/gid/24/qid/1362)

Please choose the appropriate response for each item:

|  | **strongly disagree** | **disagree** | **neither agree nor**  **disagree** | **agree** | **strongly agree** |
| --- | --- | --- | --- | --- | --- |
| **I trust that this AI system can make**  **optimal decisions for my health and well- being** |  |  |  |  |  |
| **I trust that this AI**  **system is capable of making decisions for my health and well- being** |  |  |  |  |  |
| **I trust that decisions made by this AI**  **system are at least as good if not better than that of humans** |  |  |  |  |  |

## 59 How do the following describe your opinion related to predictions made by this AI system? *

Only answer this question if the following conditions are met:

[eq5](https://haagahelia.limesurvey.net/questionAdministration/view/surveyid/566237/gid/29/qid/1357) [(/questionAdministration/view/surveyid/566237/gid/29/qid/1357)](https://haagahelia.limesurvey.net/questionAdministration/view/surveyid/566237/gid/29/qid/1357) LT [gcount](https://haagahelia.limesurvey.net/questionAdministration/view/surveyid/566237/gid/24/qid/1362) [(/questionAdministration/view/surveyid/566237/gid/24/qid/1362)](https://haagahelia.limesurvey.net/questionAdministration/view/surveyid/566237/gid/24/qid/1362)

Please choose the appropriate response for each item:

|  | **strongly disagree** | **disagree** | **neither agree nor**  **disagree** | **agree** | **strongly agree** |
| --- | --- | --- | --- | --- | --- |
| **I want to know how this AI system produces its results** |  |  |  |  |  |
| **It’s important for me to be able to understand the processes behind results and decisions of this AI system** |  |  |  |  |  |
| **Results produced by this AI system should be explained and shown in an understandable and transparent way** |  |  |  |  |  |

## 60 How do the following describe your opinion on training data of this AI system? *

Only answer this question if the following conditions are met:

[eq5](https://haagahelia.limesurvey.net/questionAdministration/view/surveyid/566237/gid/29/qid/1357) [(/questionAdministration/view/surveyid/566237/gid/29/qid/1357)](https://haagahelia.limesurvey.net/questionAdministration/view/surveyid/566237/gid/29/qid/1357) LT [gcount](https://haagahelia.limesurvey.net/questionAdministration/view/surveyid/566237/gid/24/qid/1362) [(/questionAdministration/view/surveyid/566237/gid/24/qid/1362)](https://haagahelia.limesurvey.net/questionAdministration/view/surveyid/566237/gid/24/qid/1362)

Please choose the appropriate response for each item:

|  | **strongly disagree** | **disagree** | **neither agree nor**  **disagree** | **agree** | **strongly agree** |
| --- | --- | --- | --- | --- | --- |
| **The quantity and type of data used in training this AI system should be explained to the user** |  |  |  |  |  |
| **The types and quantities of data used in training this AI**  **system do not need to be made known to users** |  |  |  |  |  |
| **It is critical that users know how much and what kinds of data are used in training this AI system** |  |  |  |  |  |

Consider the data that was used in developing and training this AI system

## 61 How do the following describe your opinion related to the developer of this AI system? *

Only answer this question if the following conditions are met:

[eq5](https://haagahelia.limesurvey.net/questionAdministration/view/surveyid/566237/gid/29/qid/1357) [(/questionAdministration/view/surveyid/566237/gid/29/qid/1357)](https://haagahelia.limesurvey.net/questionAdministration/view/surveyid/566237/gid/29/qid/1357) LT [gcount](https://haagahelia.limesurvey.net/questionAdministration/view/surveyid/566237/gid/24/qid/1362) [(/questionAdministration/view/surveyid/566237/gid/24/qid/1362)](https://haagahelia.limesurvey.net/questionAdministration/view/surveyid/566237/gid/24/qid/1362)

Please choose the appropriate response for each item:

|  | **strongly disagree** | **disagree** | **neither agree nor**  **disagree** | **agree** | **strongly agree** |
| --- | --- | --- | --- | --- | --- |
| **The technology company developing this AI system (e.g., its size, country and familiarity) are crucial in building trust with users** |  |  |  |  |  |
| **The company, its national origins and brand reputation are not crucial information for users of this AI**  **system** |  |  |  |  |  |
| **All users should be aware of which technology company has developed this AI system** |  |  |  |  |  |

Consider the company or organization that is developing and selling this AI system

## 62 How do the following describe your opinion on your data used by this AI system? *

Only answer this question if the following conditions are met:

[eq5](https://haagahelia.limesurvey.net/questionAdministration/view/surveyid/566237/gid/29/qid/1357) [(/questionAdministration/view/surveyid/566237/gid/29/qid/1357)](https://haagahelia.limesurvey.net/questionAdministration/view/surveyid/566237/gid/29/qid/1357) LT [gcount](https://haagahelia.limesurvey.net/questionAdministration/view/surveyid/566237/gid/24/qid/1362) [(/questionAdministration/view/surveyid/566237/gid/24/qid/1362)](https://haagahelia.limesurvey.net/questionAdministration/view/surveyid/566237/gid/24/qid/1362)

Please choose the appropriate response for each item:

|  | **strongly disagree** | **disagree** | **neither agree nor**  **disagree** | **agree** | **strongly agree** |
| --- | --- | --- | --- | --- | --- |
| **I don’t really care about where my data is stored, who can view it and how it is used** |  |  |  |  |  |
| **I am sensitive towards knowing where data will be stored, who can access it, and how it will be used** |  |  |  |  |  |
| **It is critical that I know how and where the data is stored, who has access and the ways in which it will be used** |  |  |  |  |  |
| **I am willing to share my personal and sensitive data to improve predictions by this AI system** |  |  |  |  |  |
| **I would not want to share my personal and sensitive data for the purpose of improving predictions by this AI system** |  |  |  |  |  |

|  | **strongly disagree** | **disagree** | **neither agree nor**  **disagree** | **agree** | **strongly agree** |
| --- | --- | --- | --- | --- | --- |
| **Sharing personal and sensitive data is meaningful to me when I know its improving the accuracy of predictions by this AI system** |  |  |  |  |  |

Consider the data collected **from you**

63 What problems and ethical issues related to this AI system can you think of?

Only answer this question if the following conditions are met:

[eq5](https://haagahelia.limesurvey.net/questionAdministration/view/surveyid/566237/gid/29/qid/1357) [(/questionAdministration/view/surveyid/566237/gid/29/qid/1357)](https://haagahelia.limesurvey.net/questionAdministration/view/surveyid/566237/gid/29/qid/1357) LT [gcount](https://haagahelia.limesurvey.net/questionAdministration/view/surveyid/566237/gid/24/qid/1362) [(/questionAdministration/view/surveyid/566237/gid/24/qid/1362)](https://haagahelia.limesurvey.net/questionAdministration/view/surveyid/566237/gid/24/qid/1362)

Please write your answer here:

Please write you response in the field below (optional)

# scenario6_mental_health

64

65

**Mental health and well-being AI system**

illustration

**Description:**

This AI system helps you maintain good and balanced mental health, and gives assistance with all ongoing psychological or mental problems. The system can predict emotional states and feelings, such as excitement, stress, depression and happiness. One can discuss with the system via voice or typing. The system provides suggestions and recommendations to maintain and improve one's mental health and also notifies when it detects or predicts potential issues. The AI system constantly learns from data and feedback. The AI system collects and analyzes the following data:

Basic medical information, medications and genetic information Mental health history with treatments and issues

Mental state, emotions and feelings

Social activity and interpersonal relationships

Now please answer questions related to this AI system.

Only answer this question if the following conditions are met:

[eq6](https://haagahelia.limesurvey.net/questionAdministration/view/surveyid/566237/gid/30/qid/1358) [(/questionAdministration/view/surveyid/566237/gid/30/qid/1358)](https://haagahelia.limesurvey.net/questionAdministration/view/surveyid/566237/gid/30/qid/1358) LT [gcount](https://haagahelia.limesurvey.net/questionAdministration/view/surveyid/566237/gid/24/qid/1362) [(/questionAdministration/view/surveyid/566237/gid/24/qid/1362)](https://haagahelia.limesurvey.net/questionAdministration/view/surveyid/566237/gid/24/qid/1362)

66 How do the following describe your opinion on using this AI system? *

Only answer this question if the following conditions are met:

[eq6](https://haagahelia.limesurvey.net/questionAdministration/view/surveyid/566237/gid/30/qid/1358) [(/questionAdministration/view/surveyid/566237/gid/30/qid/1358)](https://haagahelia.limesurvey.net/questionAdministration/view/surveyid/566237/gid/30/qid/1358) LT [gcount](https://haagahelia.limesurvey.net/questionAdministration/view/surveyid/566237/gid/24/qid/1362) [(/questionAdministration/view/surveyid/566237/gid/24/qid/1362)](https://haagahelia.limesurvey.net/questionAdministration/view/surveyid/566237/gid/24/qid/1362)

Please choose the appropriate response for each item:

|  | **strongly disagree** | **disagree** | **neither agree nor**  **disagree** | **agree** | **strongly agree** |
| --- | --- | --- | --- | --- | --- |
| **I would use this AI system** |  |  |  |  |  |
| **I think this AI system would be useful** |  |  |  |  |  |
| **I plan on using AI for these purposes** |  |  |  |  |  |

## 67 How do the following describe your opinion of trust about this AI system? *

Only answer this question if the following conditions are met:

[eq6](https://haagahelia.limesurvey.net/questionAdministration/view/surveyid/566237/gid/30/qid/1358) [(/questionAdministration/view/surveyid/566237/gid/30/qid/1358)](https://haagahelia.limesurvey.net/questionAdministration/view/surveyid/566237/gid/30/qid/1358) LT [gcount](https://haagahelia.limesurvey.net/questionAdministration/view/surveyid/566237/gid/24/qid/1362) [(/questionAdministration/view/surveyid/566237/gid/24/qid/1362)](https://haagahelia.limesurvey.net/questionAdministration/view/surveyid/566237/gid/24/qid/1362)

Please choose the appropriate response for each item:

|  | **strongly disagree** | **disagree** | **neither agree nor**  **disagree** | **agree** | **strongly agree** |
| --- | --- | --- | --- | --- | --- |
| **I trust that this AI system can make**  **optimal decisions for my health and well- being** |  |  |  |  |  |
| **I trust that this AI**  **system is capable of making decisions for my health and well- being** |  |  |  |  |  |
| **I trust that decisions made by this AI**  **system are at least as good if not better than that of humans** |  |  |  |  |  |

## 68 How do the following describe your opinion related to predictions made by this AI system? *

Only answer this question if the following conditions are met:

[eq6](https://haagahelia.limesurvey.net/questionAdministration/view/surveyid/566237/gid/30/qid/1358) [(/questionAdministration/view/surveyid/566237/gid/30/qid/1358)](https://haagahelia.limesurvey.net/questionAdministration/view/surveyid/566237/gid/30/qid/1358) LT [gcount](https://haagahelia.limesurvey.net/questionAdministration/view/surveyid/566237/gid/24/qid/1362) [(/questionAdministration/view/surveyid/566237/gid/24/qid/1362)](https://haagahelia.limesurvey.net/questionAdministration/view/surveyid/566237/gid/24/qid/1362)

Please choose the appropriate response for each item:

|  | **strongly disagree** | **disagree** | **neither agree nor**  **disagree** | **agree** | **strongly agree** |
| --- | --- | --- | --- | --- | --- |
| **I want to know how this AI system produces its results** |  |  |  |  |  |
| **It’s important for me to be able to understand the processes behind results and decisions of this AI system** |  |  |  |  |  |
| **Results produced by this AI system should be explained and shown in an understandable and transparent way** |  |  |  |  |  |

## 69 How do the following describe your opinion on training data of this AI system? *

Only answer this question if the following conditions are met:

[eq6](https://haagahelia.limesurvey.net/questionAdministration/view/surveyid/566237/gid/30/qid/1358) [(/questionAdministration/view/surveyid/566237/gid/30/qid/1358)](https://haagahelia.limesurvey.net/questionAdministration/view/surveyid/566237/gid/30/qid/1358) LT [gcount](https://haagahelia.limesurvey.net/questionAdministration/view/surveyid/566237/gid/24/qid/1362) [(/questionAdministration/view/surveyid/566237/gid/24/qid/1362)](https://haagahelia.limesurvey.net/questionAdministration/view/surveyid/566237/gid/24/qid/1362)

Please choose the appropriate response for each item:

|  | **strongly disagree** | **disagree** | **neither agree nor**  **disagree** | **agree** | **strongly agree** |
| --- | --- | --- | --- | --- | --- |
| **The quantity and type of data used in training this AI system should be explained to the user** |  |  |  |  |  |
| **The types and quantities of data used in training this AI**  **system do not need to be made known to users** |  |  |  |  |  |
| **It is critical that users know how much and what kinds of data are used in training this AI system** |  |  |  |  |  |

Consider the data that was used in developing and training this AI system

## 70 How do the following describe your opinion related to the developer of this AI system? *

Only answer this question if the following conditions are met:

[eq6](https://haagahelia.limesurvey.net/questionAdministration/view/surveyid/566237/gid/30/qid/1358) [(/questionAdministration/view/surveyid/566237/gid/30/qid/1358)](https://haagahelia.limesurvey.net/questionAdministration/view/surveyid/566237/gid/30/qid/1358) LT [gcount](https://haagahelia.limesurvey.net/questionAdministration/view/surveyid/566237/gid/24/qid/1362) [(/questionAdministration/view/surveyid/566237/gid/24/qid/1362)](https://haagahelia.limesurvey.net/questionAdministration/view/surveyid/566237/gid/24/qid/1362)

Please choose the appropriate response for each item:

|  | **strongly disagree** | **disagree** | **neither agree nor**  **disagree** | **agree** | **strongly agree** |
| --- | --- | --- | --- | --- | --- |
| **The technology company developing this AI system (e.g., its size, country and familiarity) are crucial in building trust with users** |  |  |  |  |  |
| **The company, its national origins and brand reputation are not crucial information for users of this AI**  **system** |  |  |  |  |  |
| **All users should be aware of which technology company has developed this AI system** |  |  |  |  |  |

Consider the company or organization that is developing and selling this AI system

## 71 How do the following describe your opinion on your data used by this AI system? *

Only answer this question if the following conditions are met:

[eq6](https://haagahelia.limesurvey.net/questionAdministration/view/surveyid/566237/gid/30/qid/1358) [(/questionAdministration/view/surveyid/566237/gid/30/qid/1358)](https://haagahelia.limesurvey.net/questionAdministration/view/surveyid/566237/gid/30/qid/1358) LT [gcount](https://haagahelia.limesurvey.net/questionAdministration/view/surveyid/566237/gid/24/qid/1362) [(/questionAdministration/view/surveyid/566237/gid/24/qid/1362)](https://haagahelia.limesurvey.net/questionAdministration/view/surveyid/566237/gid/24/qid/1362)

Please choose the appropriate response for each item:

|  | **strongly disagree** | **disagree** | **neither agree nor**  **disagree** | **agree** | **strongly agree** |
| --- | --- | --- | --- | --- | --- |
| **I don’t really care about where my data is stored, who can view it and how it is used** |  |  |  |  |  |
| **I am sensitive towards knowing where data will be stored, who can access it, and how it will be used** |  |  |  |  |  |
| **It is critical that I know how and where the data is stored, who has access and the ways in which it will be used** |  |  |  |  |  |
| **I am willing to share my personal and sensitive data to improve predictions by this AI system** |  |  |  |  |  |
| **I would not want to share my personal and sensitive data for the purpose of improving predictions by this AI system** |  |  |  |  |  |

|  | **strongly disagree** | **disagree** | **neither agree nor**  **disagree** | **agree** | **strongly agree** |
| --- | --- | --- | --- | --- | --- |
| **Sharing personal and sensitive data is meaningful to me when I know its improving the accuracy of predictions by this AI system** |  |  |  |  |  |

Consider the data collected **from you**

72 What problems and ethical issues related to this AI system can you think of?

Only answer this question if the following conditions are met:

[eq6](https://haagahelia.limesurvey.net/questionAdministration/view/surveyid/566237/gid/30/qid/1358) [(/questionAdministration/view/surveyid/566237/gid/30/qid/1358)](https://haagahelia.limesurvey.net/questionAdministration/view/surveyid/566237/gid/30/qid/1358) LT [gcount](https://haagahelia.limesurvey.net/questionAdministration/view/surveyid/566237/gid/24/qid/1362) [(/questionAdministration/view/surveyid/566237/gid/24/qid/1362)](https://haagahelia.limesurvey.net/questionAdministration/view/surveyid/566237/gid/24/qid/1362)

Please write your answer here:

Please write you response in the field below (optional)

# scenario7_bioelectronic_system

73

74

**Bioelectronic real-time health monitoring and adjustment AI system**

illustration

### Description:

This AI system aims to promote health by monitoring, analyzing and adjusting the

chemical composition of the body via implanted bioelectronics. The system uses smart sensors and electronics that are inserted inside the body or under the skin. Sensors collect real-time physiological data, including chemical balance, hormone levels, antibodies and blood concentration which are analyzed via AI for any anomalies and issues with health. The system has the ability to release medications and beneficial chemicals to maintain the optimal state of one's body. This AI system can help to prevent or manage health issues and diseases. The AI system constantly learns and improves from data and feedback. The AI system collects and analyzes the following data:

Vital signs and physiological indicators Full blood profile

Physical activity, sleep, rest and exercises Diet, stress levels and medications Complete personal and medical information

Now please answer questions related to this AI system.

Only answer this question if the following conditions are met:

[eq7](https://haagahelia.limesurvey.net/questionAdministration/view/surveyid/566237/gid/31/qid/1359) [(/questionAdministration/view/surveyid/566237/gid/31/qid/1359)](https://haagahelia.limesurvey.net/questionAdministration/view/surveyid/566237/gid/31/qid/1359) LT [gcount](https://haagahelia.limesurvey.net/questionAdministration/view/surveyid/566237/gid/24/qid/1362) [(/questionAdministration/view/surveyid/566237/gid/24/qid/1362)](https://haagahelia.limesurvey.net/questionAdministration/view/surveyid/566237/gid/24/qid/1362)

75 How do the following describe your opinion on using this AI system? *

Only answer this question if the following conditions are met:

[eq7](https://haagahelia.limesurvey.net/questionAdministration/view/surveyid/566237/gid/31/qid/1359) [(/questionAdministration/view/surveyid/566237/gid/31/qid/1359)](https://haagahelia.limesurvey.net/questionAdministration/view/surveyid/566237/gid/31/qid/1359) LT [gcount](https://haagahelia.limesurvey.net/questionAdministration/view/surveyid/566237/gid/24/qid/1362) [(/questionAdministration/view/surveyid/566237/gid/24/qid/1362)](https://haagahelia.limesurvey.net/questionAdministration/view/surveyid/566237/gid/24/qid/1362)

Please choose the appropriate response for each item:

|  | **strongly disagree** | **disagree** | **neither agree nor**  **disagree** | **agree** | **strongly agree** |
| --- | --- | --- | --- | --- | --- |
| **I would use this AI system** |  |  |  |  |  |
| **I think this AI system would be useful** |  |  |  |  |  |
| **I plan on using AI for these purposes** |  |  |  |  |  |

## 76 How do the following describe your opinion of trust about this AI system? *

Only answer this question if the following conditions are met:

[eq7](https://haagahelia.limesurvey.net/questionAdministration/view/surveyid/566237/gid/31/qid/1359) [(/questionAdministration/view/surveyid/566237/gid/31/qid/1359)](https://haagahelia.limesurvey.net/questionAdministration/view/surveyid/566237/gid/31/qid/1359) LT [gcount](https://haagahelia.limesurvey.net/questionAdministration/view/surveyid/566237/gid/24/qid/1362) [(/questionAdministration/view/surveyid/566237/gid/24/qid/1362)](https://haagahelia.limesurvey.net/questionAdministration/view/surveyid/566237/gid/24/qid/1362)

Please choose the appropriate response for each item:

|  | **strongly disagree** | **disagree** | **neither agree nor**  **disagree** | **agree** | **strongly agree** |
| --- | --- | --- | --- | --- | --- |
| **I trust that this AI system can make**  **optimal decisions for my health and well- being** |  |  |  |  |  |
| **I trust that this AI**  **system is capable of making decisions for my health and well- being** |  |  |  |  |  |
| **I trust that decisions made by this AI**  **system are at least as good if not better than that of humans** |  |  |  |  |  |

## 77 How do the following describe your opinion related to predictions made by this AI system? *

Only answer this question if the following conditions are met:

[eq7](https://haagahelia.limesurvey.net/questionAdministration/view/surveyid/566237/gid/31/qid/1359) [(/questionAdministration/view/surveyid/566237/gid/31/qid/1359)](https://haagahelia.limesurvey.net/questionAdministration/view/surveyid/566237/gid/31/qid/1359) LT [gcount](https://haagahelia.limesurvey.net/questionAdministration/view/surveyid/566237/gid/24/qid/1362) [(/questionAdministration/view/surveyid/566237/gid/24/qid/1362)](https://haagahelia.limesurvey.net/questionAdministration/view/surveyid/566237/gid/24/qid/1362)

Please choose the appropriate response for each item:

|  | **strongly disagree** | **disagree** | **neither agree nor**  **disagree** | **agree** | **strongly agree** |
| --- | --- | --- | --- | --- | --- |
| **I want to know how this AI system produces its results** |  |  |  |  |  |
| **It’s important for me to be able to understand the processes behind results and decisions of this AI system** |  |  |  |  |  |
| **Results produced by this AI system should be explained and shown in an understandable and transparent way** |  |  |  |  |  |

## 78 How do the following describe your opinion on training data of this AI system? *

Only answer this question if the following conditions are met:

[eq7](https://haagahelia.limesurvey.net/questionAdministration/view/surveyid/566237/gid/31/qid/1359) [(/questionAdministration/view/surveyid/566237/gid/31/qid/1359)](https://haagahelia.limesurvey.net/questionAdministration/view/surveyid/566237/gid/31/qid/1359) LT [gcount](https://haagahelia.limesurvey.net/questionAdministration/view/surveyid/566237/gid/24/qid/1362) [(/questionAdministration/view/surveyid/566237/gid/24/qid/1362)](https://haagahelia.limesurvey.net/questionAdministration/view/surveyid/566237/gid/24/qid/1362)

Please choose the appropriate response for each item:

|  | **strongly disagree** | **disagree** | **neither agree nor**  **disagree** | **agree** | **strongly agree** |
| --- | --- | --- | --- | --- | --- |
| **The quantity and type of data used in training this AI system should be explained to the user** |  |  |  |  |  |
| **The types and quantities of data used in training this AI**  **system do not need to be made known to users** |  |  |  |  |  |
| **It is critical that users know how much and what kinds of data are used in training this AI system** |  |  |  |  |  |

Consider the data that was used in developing and training this AI system

## 79 How do the following describe your opinion related to the developer of this AI system? *

Only answer this question if the following conditions are met:

[eq7](https://haagahelia.limesurvey.net/questionAdministration/view/surveyid/566237/gid/31/qid/1359) [(/questionAdministration/view/surveyid/566237/gid/31/qid/1359)](https://haagahelia.limesurvey.net/questionAdministration/view/surveyid/566237/gid/31/qid/1359) LT [gcount](https://haagahelia.limesurvey.net/questionAdministration/view/surveyid/566237/gid/24/qid/1362) [(/questionAdministration/view/surveyid/566237/gid/24/qid/1362)](https://haagahelia.limesurvey.net/questionAdministration/view/surveyid/566237/gid/24/qid/1362)

Please choose the appropriate response for each item:

|  | **strongly disagree** | **disagree** | **neither agree nor**  **disagree** | **agree** | **strongly agree** |
| --- | --- | --- | --- | --- | --- |
| **The technology company developing this AI system (e.g., its size, country and familiarity) are crucial in building trust with users** |  |  |  |  |  |
| **The company, its national origins and brand reputation are not crucial information for users of this AI**  **system** |  |  |  |  |  |
| **All users should be aware of which technology company has developed this AI system** |  |  |  |  |  |

Consider the company or organization that is developing and selling this AI system

## 80 How do the following describe your opinion on your data used by this AI system? *

Only answer this question if the following conditions are met:

[eq7](https://haagahelia.limesurvey.net/questionAdministration/view/surveyid/566237/gid/31/qid/1359) [(/questionAdministration/view/surveyid/566237/gid/31/qid/1359)](https://haagahelia.limesurvey.net/questionAdministration/view/surveyid/566237/gid/31/qid/1359) LT [gcount](https://haagahelia.limesurvey.net/questionAdministration/view/surveyid/566237/gid/24/qid/1362) [(/questionAdministration/view/surveyid/566237/gid/24/qid/1362)](https://haagahelia.limesurvey.net/questionAdministration/view/surveyid/566237/gid/24/qid/1362)

Please choose the appropriate response for each item:

|  | **strongly disagree** | **disagree** | **neither agree nor**  **disagree** | **agree** | **strongly agree** |
| --- | --- | --- | --- | --- | --- |
| **I don’t really care about where my data is stored, who can view it and how it is used** |  |  |  |  |  |
| **I am sensitive towards knowing where data will be stored, who can access it, and how it will be used** |  |  |  |  |  |
| **It is critical that I know how and where the data is stored, who has access and the ways in which it will be used** |  |  |  |  |  |
| **I am willing to share my personal and sensitive data to improve predictions by this AI system** |  |  |  |  |  |
| **I would not want to share my personal and sensitive data for the purpose of improving predictions by this AI system** |  |  |  |  |  |

|  | **strongly disagree** | **disagree** | **neither agree nor**  **disagree** | **agree** | **strongly agree** |
| --- | --- | --- | --- | --- | --- |
| **Sharing personal and sensitive data is meaningful to me when I know its improving the accuracy of predictions by this AI system** |  |  |  |  |  |

Consider the data collected **from you**

81 What problems and ethical issues related to this AI system can you think of?

Only answer this question if the following conditions are met:

[eq7](https://haagahelia.limesurvey.net/questionAdministration/view/surveyid/566237/gid/31/qid/1359) [(/questionAdministration/view/surveyid/566237/gid/31/qid/1359)](https://haagahelia.limesurvey.net/questionAdministration/view/surveyid/566237/gid/31/qid/1359) LT [gcount](https://haagahelia.limesurvey.net/questionAdministration/view/surveyid/566237/gid/24/qid/1362) [(/questionAdministration/view/surveyid/566237/gid/24/qid/1362)](https://haagahelia.limesurvey.net/questionAdministration/view/surveyid/566237/gid/24/qid/1362)

Please write your answer here:

Please write you response in the field below (optional)

# scenario8_nurse_robot

82

83

**AI-controlled robotic nursing and caregiving system**

illustration

### Description:

This AI system can perform nursing and caregiving activities for patients and the elderly. The machine is equipped with advanced sensors and mechanical limbs to provide care and support to patients without the need for human intervention. The robot uses real-time sensor data with cameras and microphones to monitor the patient's condition and behaviour. The system administers medications, feeds, bathes, adjusts the patient's position and provides emotional support as needed. One can communicate with AI via talking or typing. The AI system learns via interactions and feedback. The AI system collects and analyzes the following data:

Health records with the complete medical history Real-time sensor data

Vital signs, activity and behavioural information Diet, hydration and medication information

Social contacts and interactions

Now please answer questions related to this AI system.

Only answer this question if the following conditions are met:

[eq8](https://haagahelia.limesurvey.net/questionAdministration/view/surveyid/566237/gid/32/qid/1360) [(/questionAdministration/view/surveyid/566237/gid/32/qid/1360)](https://haagahelia.limesurvey.net/questionAdministration/view/surveyid/566237/gid/32/qid/1360) LT [gcount](https://haagahelia.limesurvey.net/questionAdministration/view/surveyid/566237/gid/24/qid/1362) [(/questionAdministration/view/surveyid/566237/gid/24/qid/1362)](https://haagahelia.limesurvey.net/questionAdministration/view/surveyid/566237/gid/24/qid/1362)

84 How do the following describe your opinion on using this AI system? *

Only answer this question if the following conditions are met:

[eq8](https://haagahelia.limesurvey.net/questionAdministration/view/surveyid/566237/gid/32/qid/1360) [(/questionAdministration/view/surveyid/566237/gid/32/qid/1360)](https://haagahelia.limesurvey.net/questionAdministration/view/surveyid/566237/gid/32/qid/1360) LT [gcount](https://haagahelia.limesurvey.net/questionAdministration/view/surveyid/566237/gid/24/qid/1362) [(/questionAdministration/view/surveyid/566237/gid/24/qid/1362)](https://haagahelia.limesurvey.net/questionAdministration/view/surveyid/566237/gid/24/qid/1362)

Please choose the appropriate response for each item:

|  | **strongly disagree** | **disagree** | **neither agree nor**  **disagree** | **agree** | **strongly agree** |
| --- | --- | --- | --- | --- | --- |
| **I would use this AI system** |  |  |  |  |  |
| **I think this AI system would be useful** |  |  |  |  |  |
| **I plan on using AI for these purposes** |  |  |  |  |  |

## 85 How do the following describe your opinion of trust about this AI system? *

Only answer this question if the following conditions are met:

[eq8](https://haagahelia.limesurvey.net/questionAdministration/view/surveyid/566237/gid/32/qid/1360) [(/questionAdministration/view/surveyid/566237/gid/32/qid/1360)](https://haagahelia.limesurvey.net/questionAdministration/view/surveyid/566237/gid/32/qid/1360) LT [gcount](https://haagahelia.limesurvey.net/questionAdministration/view/surveyid/566237/gid/24/qid/1362) [(/questionAdministration/view/surveyid/566237/gid/24/qid/1362)](https://haagahelia.limesurvey.net/questionAdministration/view/surveyid/566237/gid/24/qid/1362)

Please choose the appropriate response for each item:

|  | **strongly disagree** | **disagree** | **neither agree nor**  **disagree** | **agree** | **strongly agree** |
| --- | --- | --- | --- | --- | --- |
| **I trust that this AI system can make**  **optimal decisions for my health and well- being** |  |  |  |  |  |
| **I trust that this AI**  **system is capable of making decisions for my health and well- being** |  |  |  |  |  |
| **I trust that decisions made by this AI**  **system are at least as good if not better than that of humans** |  |  |  |  |  |

## 86 How do the following describe your opinion related to predictions made by this AI system? *

Only answer this question if the following conditions are met:

[eq8](https://haagahelia.limesurvey.net/questionAdministration/view/surveyid/566237/gid/32/qid/1360) [(/questionAdministration/view/surveyid/566237/gid/32/qid/1360)](https://haagahelia.limesurvey.net/questionAdministration/view/surveyid/566237/gid/32/qid/1360) LT [gcount](https://haagahelia.limesurvey.net/questionAdministration/view/surveyid/566237/gid/24/qid/1362) [(/questionAdministration/view/surveyid/566237/gid/24/qid/1362)](https://haagahelia.limesurvey.net/questionAdministration/view/surveyid/566237/gid/24/qid/1362)

Please choose the appropriate response for each item:

|  | **strongly disagree** | **disagree** | **neither agree nor**  **disagree** | **agree** | **strongly agree** |
| --- | --- | --- | --- | --- | --- |
| **I want to know how this AI system produces its results** |  |  |  |  |  |
| **It’s important for me to be able to understand the processes behind results and decisions of this AI system** |  |  |  |  |  |
| **Results produced by this AI system should be explained and shown in an understandable and transparent way** |  |  |  |  |  |

## 87 How do the following describe your opinion on training data of this AI system? *

Only answer this question if the following conditions are met:

[eq8](https://haagahelia.limesurvey.net/questionAdministration/view/surveyid/566237/gid/32/qid/1360) [(/questionAdministration/view/surveyid/566237/gid/32/qid/1360)](https://haagahelia.limesurvey.net/questionAdministration/view/surveyid/566237/gid/32/qid/1360) LT [gcount](https://haagahelia.limesurvey.net/questionAdministration/view/surveyid/566237/gid/24/qid/1362) [(/questionAdministration/view/surveyid/566237/gid/24/qid/1362)](https://haagahelia.limesurvey.net/questionAdministration/view/surveyid/566237/gid/24/qid/1362)

Please choose the appropriate response for each item:

|  | **strongly disagree** | **disagree** | **neither agree nor**  **disagree** | **agree** | **strongly agree** |
| --- | --- | --- | --- | --- | --- |
| **The quantity and type of data used in training this AI system should be explained to the user** |  |  |  |  |  |
| **The types and quantities of data used in training this AI**  **system do not need to be made known to users** |  |  |  |  |  |
| **It is critical that users know how much and what kinds of data are used in training this AI system** |  |  |  |  |  |

Consider the data that was used in developing and training this AI system

## 88 How do the following describe your opinion related to the developer of this AI system? *

Only answer this question if the following conditions are met:

[eq8](https://haagahelia.limesurvey.net/questionAdministration/view/surveyid/566237/gid/32/qid/1360) [(/questionAdministration/view/surveyid/566237/gid/32/qid/1360)](https://haagahelia.limesurvey.net/questionAdministration/view/surveyid/566237/gid/32/qid/1360) LT [gcount](https://haagahelia.limesurvey.net/questionAdministration/view/surveyid/566237/gid/24/qid/1362) [(/questionAdministration/view/surveyid/566237/gid/24/qid/1362)](https://haagahelia.limesurvey.net/questionAdministration/view/surveyid/566237/gid/24/qid/1362)

Please choose the appropriate response for each item:

|  | **strongly disagree** | **disagree** | **neither agree nor**  **disagree** | **agree** | **strongly agree** |
| --- | --- | --- | --- | --- | --- |
| **The technology company developing this AI system (e.g., its size, country and familiarity) are crucial in building trust with users** |  |  |  |  |  |
| **The company, its national origins and brand reputation are not crucial information for users of this AI**  **system** |  |  |  |  |  |
| **All users should be aware of which technology company has developed this AI system** |  |  |  |  |  |

Consider the company or organization that is developing and selling this AI system

## 89 How do the following describe your opinion on your data used by this AI system? *

Only answer this question if the following conditions are met:

[eq8](https://haagahelia.limesurvey.net/questionAdministration/view/surveyid/566237/gid/32/qid/1360) [(/questionAdministration/view/surveyid/566237/gid/32/qid/1360)](https://haagahelia.limesurvey.net/questionAdministration/view/surveyid/566237/gid/32/qid/1360) LT [gcount](https://haagahelia.limesurvey.net/questionAdministration/view/surveyid/566237/gid/24/qid/1362) [(/questionAdministration/view/surveyid/566237/gid/24/qid/1362)](https://haagahelia.limesurvey.net/questionAdministration/view/surveyid/566237/gid/24/qid/1362)

Please choose the appropriate response for each item:

|  | **strongly disagree** | **disagree** | **neither agree nor**  **disagree** | **agree** | **strongly agree** |
| --- | --- | --- | --- | --- | --- |
| **I don’t really care about where my data is stored, who can view it and how it is used** |  |  |  |  |  |
| **I am sensitive towards knowing where data will be stored, who can access it, and how it will be used** |  |  |  |  |  |
| **It is critical that I know how and where the data is stored, who has access and the ways in which it will be used** |  |  |  |  |  |
| **I am willing to share my personal and sensitive data to improve predictions by this AI system** |  |  |  |  |  |
| **I would not want to share my personal and sensitive data for the purpose of improving predictions by this AI system** |  |  |  |  |  |

|  | **strongly disagree** | **disagree** | **neither agree nor**  **disagree** | **agree** | **strongly agree** |
| --- | --- | --- | --- | --- | --- |
| **Sharing personal and sensitive data is meaningful to me when I know its improving the accuracy of predictions by this AI system** |  |  |  |  |  |

Consider the data collected **from you**

90 What problems and ethical issues related to this AI system can you think of?

Only answer this question if the following conditions are met:

[eq8](https://haagahelia.limesurvey.net/questionAdministration/view/surveyid/566237/gid/32/qid/1360) [(/questionAdministration/view/surveyid/566237/gid/32/qid/1360)](https://haagahelia.limesurvey.net/questionAdministration/view/surveyid/566237/gid/32/qid/1360) LT [gcount](https://haagahelia.limesurvey.net/questionAdministration/view/surveyid/566237/gid/24/qid/1362) [(/questionAdministration/view/surveyid/566237/gid/24/qid/1362)](https://haagahelia.limesurvey.net/questionAdministration/view/surveyid/566237/gid/24/qid/1362)

Please write your answer here:

Please write you response in the field below (optional)

# Final_questions

91

Part 3

In this last part, consider AI systems in **healthcare and well-being in general**, including those scenarios described previously.

92 How do the following describe your opinion

on using AI systems in healthcare and well-being?

*

Please choose the appropriate response for each item:

|  | **strongly disagree** | **disagree** | **neither agree nor**  **disagree** | **agree** | **strongly agree** |
| --- | --- | --- | --- | --- | --- |
| **Given the chance, I predict that I would prefer AI systems in the future** |  |  |  |  |  |
| **It is likely that I will use AI systems in the near future** |  |  |  |  |  |
| **Given the opportunity, I intend to use AI systems in the future** |  |  |  |  |  |

93 How do the following describe your trust on AI applications in healthcare and well-being? *

Please choose the appropriate response for each item:

|  | **strongly disagree** | **disagree** | **neither agree or disagree** | **agree** | **strongly agree** |
| --- | --- | --- | --- | --- | --- |
| **It is easy for me to trust AI applications** |  |  |  |  |  |
| **My tendency to trust AI applications is high** |  |  |  |  |  |
| **I tend to trust AI applications even though I have little knowledge** |  |  |  |  |  |

## 94 How do the following describe your attitude towards AI in healthcare and well-being? *

Please choose the appropriate response for each item:

|  | **strongly disagree** | **disagree** | **neither agree nor**  **disagree** | **agree** | **strongly agree** |
| --- | --- | --- | --- | --- | --- |
| **AI will provide solutions to many of our problems** |  |  |  |  |  |
| **With AI anything is possible** |  |  |  |  |  |
| **I feel that I get more accomplished because of AI** |  |  |  |  |  |
| **AI decreases people's own control** |  |  |  |  |  |
| **AI makes life more uncertain** |  |  |  |  |  |
| **AI makes people jobless** |  |  |  |  |  |

## 95 How do the following describe your opinion on cybersecurity of AI in healthcare and well-being? *

Please choose the appropriate response for each item:

|  | **strongly disagree** | **disagree** | **neither agree nor**  **disagree** | **agree** | **strongly agree** |
| --- | --- | --- | --- | --- | --- |
| **Issues with confidentiality may be experienced via the nature of AI** |  |  |  |  |  |
| **People will treat AI the same way they treat the internet, using it**  **for information seeking without considering potential threats** |  |  |  |  |  |
| **I do not feel that the AI can responsibly guarantee confidentiality** |  |  |  |  |  |
| **I think these AI application are prone to security threats** |  |  |  |  |  |

## 96 Assess the following claims of AI in healthcare and well-being. *

Please choose the appropriate response for each item:

|  | **strongly disagree** | **disagree** | **neither agree nor**  **disagree** | **agree** | **strongly agree** |
| --- | --- | --- | --- | --- | --- |
| **I am not sure on how accurate AI systems are** |  |  |  |  |  |
| **I do not know whether or not AI systems are as accurate as humans** |  |  |  |  |  |
| **The online dominance of AI information retrieval makes me sceptical about its accuracy** |  |  |  |  |  |
| **AI may have a lot of potential, but it is not ready yet** |  |  |  |  |  |
| **The performance of AI systems and its flaws tells me that the technology is not mature** |  |  |  |  |  |
| **The state of the technology is not yet as accurate or efficient as human experts** |  |  |  |  |  |
| **I do not think that AI can replace human- human interaction** |  |  |  |  |  |

|  | **strongly disagree** | **disagree** | **neither agree nor**  **disagree** | **agree** | **strongly agree** |
| --- | --- | --- | --- | --- | --- |
| **The thought of AI seems to instill social isolation** |  |  |  |  |  |
| **You will not get a deep and meaningful conversation from a AI system** |  |  |  |  |  |

## 97 How do the following describe your opinion on accessibility of AI in healthcare and well-being? *

Please choose the appropriate response for each item:

|  | **strongly disagree** | **disagree** | **neither agree nor**  **disagree** | **agree** | **strongly agree** |
| --- | --- | --- | --- | --- | --- |
| **People of all types of abilities should be able to easily use AI** |  |  |  |  |  |
| **AI should be usable and accessible for everyone regardless of abilities** |  |  |  |  |  |
| **Accessible AI increases opportunities for both health and wellbeing practitioners and patients with disabilities** |  |  |  |  |  |
| **AI should be able to process input and**  **offer output for people of diverse abilities** |  |  |  |  |  |
| **AI can support people with diverse needs** |  |  |  |  |  |
| **I feel concerned about people with special needs interacting with AI** |  |  |  |  |  |

Accessibility means taking account of people's differences and special needs

98 What concerns do you have related to people with special needs interacting with AI?

Please write you response in the field below (optional).

Only answer this question if the following conditions are met:

Answer was 'agree' *or* 'strongly agree' at question ' [G13Q00005]' (How do the following describe your opinion on accessibility of AI in healthcare and well-being? (I feel concerned about people with special needs interacting with AI))

Please write your answer here:

## Thank you for responding!

If you have any questions or you want to give feedback, please contact: Janne Kauttonen, [janne.kauttonen@haaga-helia.fi](mailto:janne.kauttonen@haaga-helia.fi)

23.5.2023 – 11:36

Submit your survey.

Thank you for completing this survey.
