# Supplementary material for: Trust and Acceptance Challenges in the Adoption of AI Applications in Health Care: Quantitative Survey Analysis
Source: J Med Internet Res. 2025 Mar 21;27:e65567. doi: 10.2196/65567 (PMC11971584; doi:10.2196/65567)
Supplement: Multimedia Appendix 3 [file jmir_v27i1e65567_app3.docx]

Descriptions of use cases

Descriptions of all 8 use cases as presented to responders. All texts were presented in Finnish and English (selectable in the beginning of the survey).

1. **Activity monitoring AI system to support a healthy lifestyle**

**
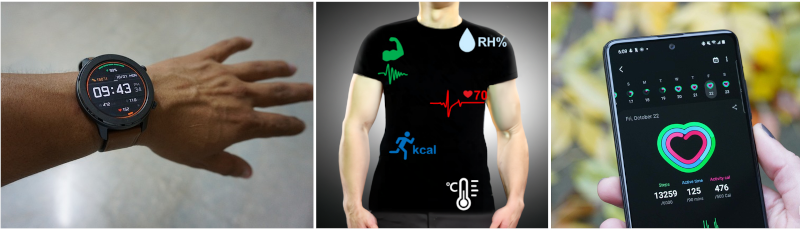
**

This AI system aims to promote a healthy and active lifestyle by monitoring and analyzing physical activity and well-being. The system can measure the status of the body using a combination of a smartwatch and/or ring with wearable sensors embedded in clothes. The system can help in optimizing daily physical activities and exercises by creating individual workout plans and suggesting healthy options. The AI system constantly learns and improves from data and feedback. The AI system collects and analyzes the following data:

- Heart rate and stroke volume
- Blood oxygenation and pressure
- Electrical activity of the muscles
- Respiration rhythm
- Body temperature and perspiration
- Location data

1. **Menstrual cycle monitoring and prediction AI system**


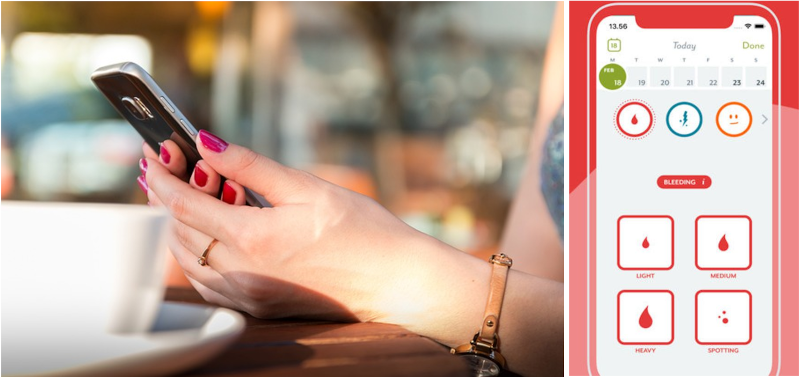


“The menstrual cycle monitoring and predicting AI system is designed to help track and predict the status of the menstrual cycle. This AI system can provide accurate predictions of when the next period is due, most likely ovulation time, and report any changes in the menstrual cycle that may require attention from healthcare services. It can be used for example to plan for pregnancy, track fertility and monitor hormonal levels. The AI system constantly learns from data and feedback. The AI system collects and analyzes the following data:

- Menstrual cycle history
- Symptoms such as cramps, bloating, headaches, mood changes
- Body temperature
- Hormonal birth control use
- Physical and sexual activity
- Status of diet, stress and medications

Note: You can answer on your own behalf or from the perspective of your (female)
partner. You can also skip this scenario and move to the next one by leaving all
responses empty.

1. **AI-controlled robotic surgeon**


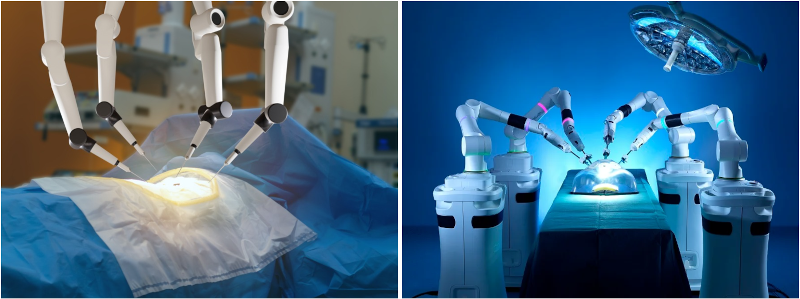


The robotic surgeon is an AI-controlled system that can perform various types of
surgeries for human patients. The machine is equipped with advanced sensors and
mechanical instruments designed to perform surgical procedures autonomously and
accurately without human intervention. The system uses real-time data from various
imaging technologies (e.g., laser scanning, MRI, CT, and ultrasound) to create an
accurate 3D map of the patient's anatomy. The AI analyzes data and controls trajectories, speeds, and forces of instruments to predict and perform the operation optimally. The AI system collects and analyzes the following data:

- Full health records, medical history and genetic data
- Imaging data including laser scanning, MRI, CT and ultrasound
- Accurate, real-time sensor data to track patient physiology

1. **Nutrition monitoring and planning AI system**


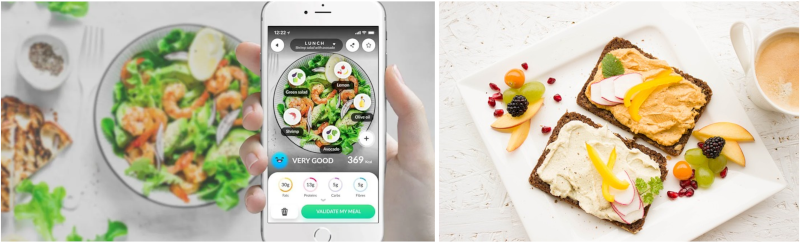


This AI system aims to promote a healthy diet by monitoring and analyzing consumed meals and activities. The system analyzes all meals, calorie consumption, physical activity, rest and taste priorities to create an individualized and optimized meal plan. Personalized diets are based on predicted blood glucose responses and energy consumption. The AI system constantly learns from data and feedback. The AI system collects and analyzes the following data:

- Physical activity including sports, rest and sleep
- Information on consumed nutrients, meals and drinks
- Body state and composition, including weight and fat percentage
- Medical conditions, medications and food allergies

1. **Real-time health monitoring, analysis and prediction AI system**


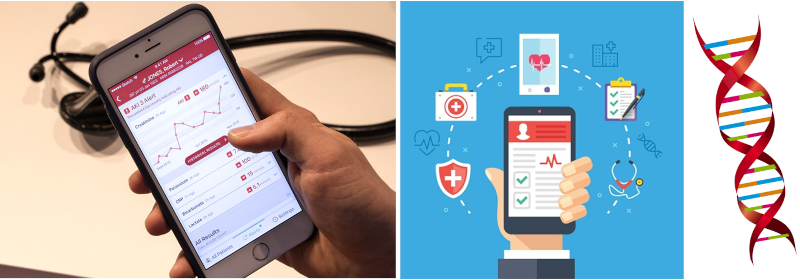


This AI system monitors personal physical health and helps with any medical issues and treatments. The system can detect and predict any changes in personal health and provide recommendations on how one can improve and maintain their health, or whether there is a need to seek medical help. The system leverages your personal and medical information and takes advantage of your genetic profile to make predictions and recommendations. The AI system constantly learns from data and feedback. The AI system collects and analyzes the following data:

- Physical health history, such as any issues with health and treatments
- Basic medical information, such as medication and illnesses
- Physical activity information
- Genetic data
- Basic demographic information

1. **Mental health and well-being AI system**


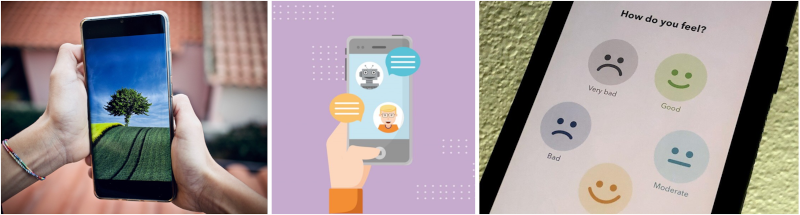


This AI system helps you maintain good and balanced mental health, and gives
assistance with all ongoing psychological or mental problems. The system can predict emotional states and feelings, such as excitement, stress, depression and happiness. One can discuss with the system via voice or typing. The system provides suggestions and recommendations to maintain and improve one's mental health and also notifies when it detects or predicts potential issues. The AI system constantly learns from data and feedback. The AI system collects and analyzes the following data:

- Basic medical information, medications and genetic information
- Mental health history with treatments and issues
- Mental state, emotions and feelings
- Social activity and interpersonal relationships

1. **Bioelectronic real-time health monitoring and adjustment AI system**


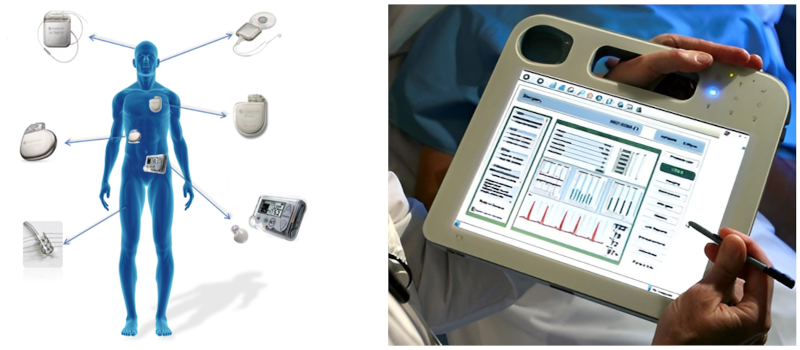


This AI system aims to promote health by monitoring, analyzing and adjusting the
chemical composition of the body via implanted bioelectronics. The system uses smart sensors and electronics that are inserted inside the body or under the skin. Sensors collect real-time physiological data, including chemical balance, hormone levels, antibodies and blood concentration which are analyzed via AI for any anomalies and issues with health. The system has the ability to release medications and beneficial chemicals to maintain the optimal state of one's body. This AI system can help to prevent or manage health issues and diseases. The AI system constantly learns and improves from data and feedback. The AI system collects and analyzes the following data:

- Vital signs and physiological indicators
- Full blood profile
- Physical activity, sleep, rest and exercises
- Diet, stress levels and medications
- Complete personal and medical information

1. **AI-controlled robotic nursing and caregiving system**


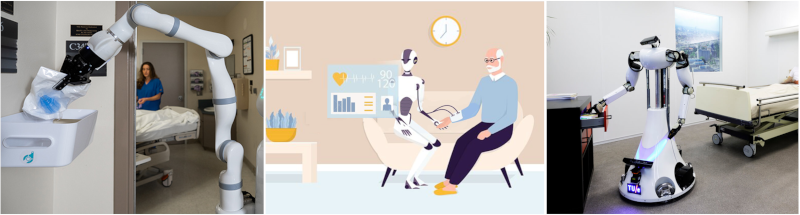


This AI system can perform nursing and caregiving activities for patients and the elderly. The machine is equipped with advanced sensors and mechanical limbs to provide care and support to patients without the need for human intervention. The robot uses real-time sensor data with cameras and microphones to monitor the patient's condition and behaviour. The system administers medications, feeds, bathes, adjusts the patient's position and provides emotional support as needed. One can communicate with AI via talking or typing. The AI system learns via interactions and feedback. The AI system collects and analyzes the following data:

- Health records with the complete medical history
- Real-time sensor data
- Vital signs, activity and behavioural information
- Diet, hydration and medication information
- Social contacts and interactions
