# Supplementary material for: Trust and Acceptance Challenges in the Adoption of AI Applications in Health Care: Quantitative Survey Analysis
Source: J Med Internet Res. 2025 Mar 21;27:e65567. doi: 10.2196/65567 (PMC11971584; doi:10.2196/65567)
Supplement: Multimedia Appendix 4 [file jmir_v27i1e65567_app4.docx]

Predictor variables

Table S1 contains the list of predictor variables included in the analysis and training predictive regression models, i.e., model 1 for use cases and model 2 for overall opinions (no use_case variable included). As part of preprocessing, some responses were combined in response to rare responses and also to reduce number of predictor variables that help avoiding model overfitting. Here variable descriptions are also shortened from their longer texts shown in the survey. To see full original questions and all response options, see Multimedia Appendix 1 that contains the copy of the full survey.

**Table S1.** Predictor variables used in the analysis, including descriptions and variable types and available response options. The bolded response was the most common (i.e., mode or mean) response found in our data.

| **Variable name** | **Variable description** | **Variable type and options** |
| --- | --- | --- |
| age | The current age in years | Numerical: **49** (range 18-87) |
| gender | Gender identity | Categorical: “Male”, “**Female**”, “Nonbinary or would not say” |
| education_level | Highest level of education | Categorical: "Elementary school", "**High school or vocational cert**.", "Undergraduate", "Graduate degree", "Postgraduate degree" |
| education_field_education | Education from education (pedagogy) field | Binary: “yes”, “**no**” |
| education_field_business_services | Education from commerce, administration, law or service field |  |
| education_field_science_ICT_engineer | Education from science, Information and communication technology (ICT) or engineering field |  |
| education_field_health | Education from health and wellness field |  |
| education_field_others | Education from any other field |  |
| work_field_education | Working in education sector | Binary: “yes”, “**no**” |
| work_field_business_services | Working in commerce, administration, law or service sector |  |
| work_field_science_ICT_engineer | Working in science, Information and communication technology (ICT) or engineering sector |  |
| work_field_health | Working in health and wellness sector |  |
| work_field_others | Working in any other sector |  |
|  | How do following statements apply to one’s personality: |  |
| persona_critical_quarrelsome | critical, quarrelsome | Ordinal: "strongly disagree", "moderately disagree", "disagree a little", "neither agree nor disagree", "**agree a little**", "agree moderately", "agree strongly" |
| persona_dependable_selfdisciplined | dependable, self-disciplined |  |
| persona_anxious_easilyupset | anxious, easily upset |  |
| persona_newexperiences_complex | open to new experiences, complex |  |
| persona_reserved_quiet | reserved, quiet |  |
| persona_sympathetic_warm | sympathetic, warm |  |
| persona_calm_stable | calm, emotionally stable |  |
| persona_conventional_uncreative | conventional, uncreative |  |
| persona_disorganized_careless | disorganized, careless |  |
| persona_extraverted_enthusiastic | extraverted, enthusiastic |  |
| IT_skills | Self-evaluated IT skills compared to others | Ordinal: “Very poor”, “Poor”,  “**Average**”, “Good”, “Very good“ |
| technology_adoption | Self-evaluated adoption and usage of new technology | Categorical: "Falling behind others, late adopter", "**Keeping up with others**", "Ahead of others, early adopter" |
| health_status | Self-evaluated overall health status in the past 12 months | Ordinal: "Very bad", "Bad", "Not good or bad", "**Good**", "Very good" |
| health_service_usage | Frequency of health services usage past 12 months | Categorical: "Weekly or more", "A few times per month", "Approximately monthly", "**A few times or not at all**" |
| healthcare_services | How do one feel about the current state of healthcare services (private and public in Finland) | Ordinal: "Very unsatisfied", "**Unsatisfied**", "Not satisfied or unsatisfied", "Satisfied", "Very satisfied" |
| exercise_level | Amount of exercise and physical straining of oneself in free time | Categorical: "Regular strenuous sports several times a week", "Several hours a week", "**Lightly several hours a week**", "Do not move much or strain oneself physically" |
| technology_usage | Frequency of using technology to monitor own health and well-being | Ordinal: "Always", "Often", "**Sometimes**", "Seldom", "Never" |
| technology_attitude | Self-evaluated attitude towards new technologies (a factor construct from 4 Likert-type questions) | Numerical (**0** with range -1.93,…,2.02) |
| AI_knowledge | Self-evaluated knowledge and experience of AI (a summary construct from 5 Likert-type questions) | Numerical (**0.47** with range  -2,…,2) |
| use_case | Eight use cases from healthcare and wellbeing sector | Categorical: "activitymonitor", "menstrual", "robotsurgeon", "nutrition", "healthmonitor", "mentalmonitor","bioelectric", "nursing" |

During preprocessing and before modeling, we used the following mapping of ordinal responses into numerical ones:

- For 7-point Likert scale: "strongly disagree" -3, "disagree moderately" -2, "disagree a little" -1, "neither agree nor disagree" 0, "agree a little" 1, "agree moderately" 2, "'strongly agree": 3,
- For 5-point Likert scale: "strongly disagree" -2, "disagree" -1, "neither agree nor disagree" 0, "agree" 1, "strongly agree" 2.
- For IT skills and health status compared to others (variables IT_skills and health_status): "Very good" 2, "Good" 1, "Average / Not good or bad" 0, "Poor/Bad" -1, "Very poor/bad" -2.
- For opinion on healthcare services (variable healthcare_services): "Very satisfied" 2, "Satisfied" 1, "Not satisfied or unsatisfied" 0, "Unsatisfied" -1, "Very unsatisfied" -2.
