# Supplementary material for: Trust and Acceptance Challenges in the Adoption of AI Applications in Health Care: Quantitative Survey Analysis
Source: J Med Internet Res. 2025 Mar 21;27:e65567. doi: 10.2196/65567 (PMC11971584; doi:10.2196/65567)
Supplement: Multimedia Appendix 5 [file jmir_v27i1e65567_app5.docx]

Gradient boosting models

After using Optuna (<https://optuna.org>), the optimized key hyperparameters for the development data were: random_strength 11.35, iterations 1397, max depth 5, learning rate 0.0091 and l2_leaf_reg 0.0331. To better understand the predictions and errors for model 1, explained variance R^2^ values were computed separately for each use case and response. The results are shown in Table S1. The highest accuracies with over 10% variance explained were obtained for Intention (0.235), Trust (0.168) and Trade-off (0.132). There was no notable difference for use-cases as the R^2^ values varied between 0.084 and 0.102.

**Table S1.** Explained variance measured with R^2^ for the test data (1125 datapoints from 192 subjects) for the multi-variate regression model 1. Here we have split data between 8 use-cases and 7 output dimensions to provide both individual and combined values.

|  | **Activity monitor** | **Bioelectric device** | **Health monitor** | **Menstrual monitor** | **Mental-health monitor** | **Nursing assistant** | **Nutrition assistant** | **Robot surgeon** | **all** |
| --- | --- | --- | --- | --- | --- | --- | --- | --- | --- |
| **Intention** | 0.209 | 0.191 | 0.163 | 0.184 | 0.177 | 0.138 | 0.235 | 0.105 | 0.235 |
| **Trust** | 0.058 | 0.199 | 0.156 | 0.135 | 0.194 | 0.063 | 0.228 | 0.086 | 0.168 |
| **Predictions** | 0.056 | 0.064 | 0.069 | 0.052 | 0.002 | 0.091 | 0.095 | 0.113 | 0.067 |
| **Data** | 0.063 | 0.032 | 0.042 | 0.028 | 0.018 | 0.047 | 0.072 | 0.098 | 0.052 |
| **Privacy** | 0.113 | 0.099 | 0.066 | 0.045 | 0.051 | 0.077 | 0.078 | 0.130 | 0.085 |
| **Trade-off** | 0.096 | 0.146 | 0.133 | 0.160 | 0.093 | 0.201 | 0.153 | 0.093 | 0.132 |
| **Manufacturer** | 0.050 | 0.015 | 0.016 | 0.016 | 0.013 | 0.023 | 0.060 | 0.076 | 0.033 |
| **all** | 0.092 | 0.098 | 0.084 | 0.095 | 0.102 | 0.094 | 0.136 | 0.093 | 0.105 |

For model 2 (part 3 of the survey), the procedure was similar as for model 1. We trained a Catboost model for Intention and Trust. The final model reached R^2^ of 0.225 with 0.235 for Intention and 0.220 for Trust only.
