# Supplementary material for: Trust and Acceptance Challenges in the Adoption of AI Applications in Health Care: Quantitative Survey Analysis
Source: J Med Internet Res. 2025 Mar 21;27:e65567. doi: 10.2196/65567 (PMC11971584; doi:10.2196/65567)
Supplement: Multimedia Appendix 6 [file jmir_v27i1e65567_app6.docx]

Results for overall Intention and Trust

*Measurement constructs for the overall usage of AI in healthcare and well-being*

In part 3 of the survey, we asked for opinions on the overall usage of AI in the field of healthcare and well-being, not related to any particular use case. The number of valid data points was 1086 (one per subject). The confirmatory factor analysis for response variables (total 14), resulted in a model that covered 11 items in 4 constructs. See Multimedia Appendix 1 and Table S2 for fit parameters and items.

The four-factor construct, and their interpretations were as follows:

- **Intention (3 items)**: Intention to use the AI system if offered a chance
- **Trust (3 items)**: Tendency to trust AI applications
- **Cybersecurity (2 items)**: AI applications cannot guarantee confidentiality
- **Accessibility (3 items)**: AI should be usable for everyone regardless of abilities

After fitting the factor measurement models, the means of the factors were extracted by computing weighted averages of item values over the original data. The results are depicted in Fig. S1 including the means and 75% percentile coverage. Accessibility and Cybersecurity were considered most important, with mean values close to “agree”. Interestingly, there was a notable difference between Intention and Trust, with the latter having a negative mean value (towards “disagree”).


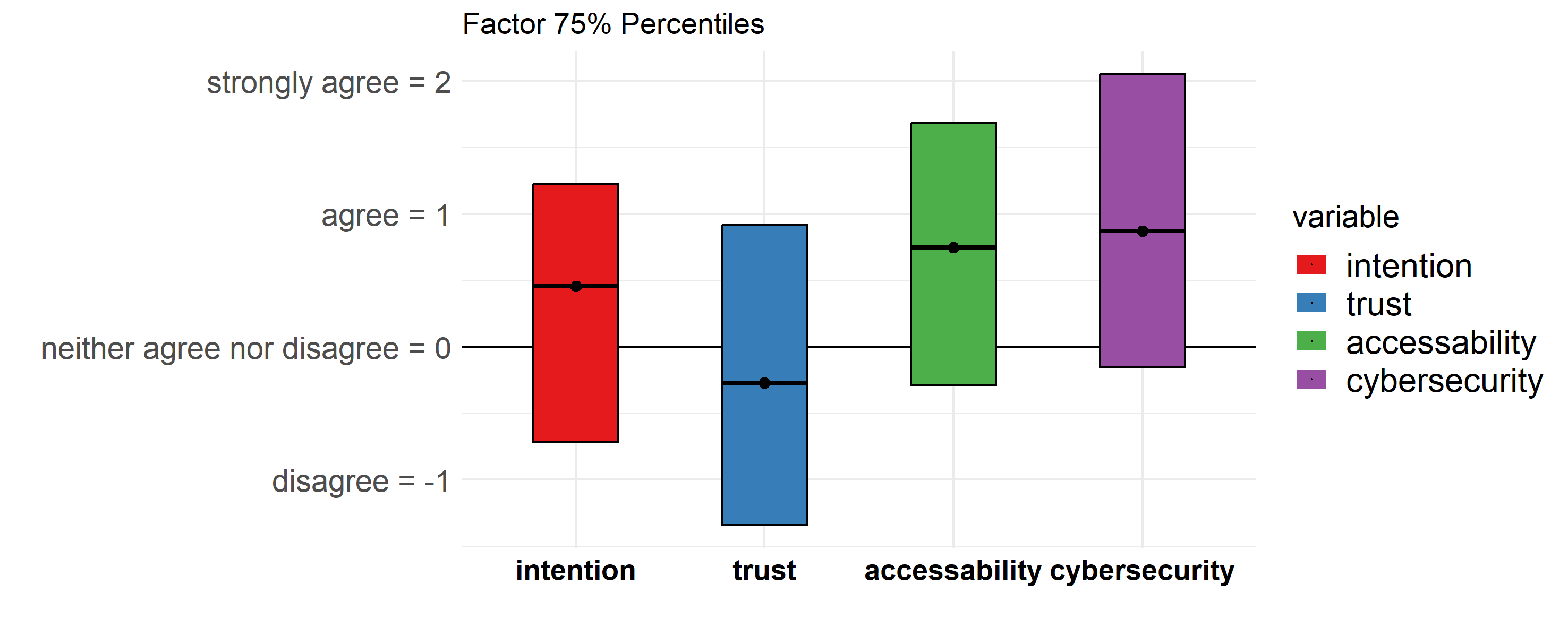


**Figure S1.** Response factors for the overall case (part 3 of the survey; n=1086 subjects) with their means (black dots) and 75% percentiles (bars) around means. The corresponding Likert-scale is shown on the y-axis.

Correlations between factors are listed in Table S1 with all being far from zero (at p<0.01), except between Accessibility and Cybersecurity with a correlation value -0.012.

**Table S1.** Correlation coefficients (n=1086 subjects) between four predictors (factors) for overall AI use in healthcare and well-being (part 3 of the survey). All values bolded were significant at p<0.01 using a two-tailed permutation test against zero (5000 iterations).

|  | **Trust** | **Accessibility** | **Cybersecurity** |
| --- | --- | --- | --- |
| **Intention** | **0.768** | **0.391** | **-0.313** |
| **Trust** |  | **0.246** | **-0.591** |
| **Accessibility** |  |  | -0.012 |

The optimal hyperparameters for a trained Catboost predictive model for the overall Intention and Trust were random_strength 4.94, iterations 400, max_depth 3, learning_rate 0.052 and l2_leaf_reg 18.878. Fig. S2 depicts the feature importance of all predictors measured via mean absolute SHAP values.


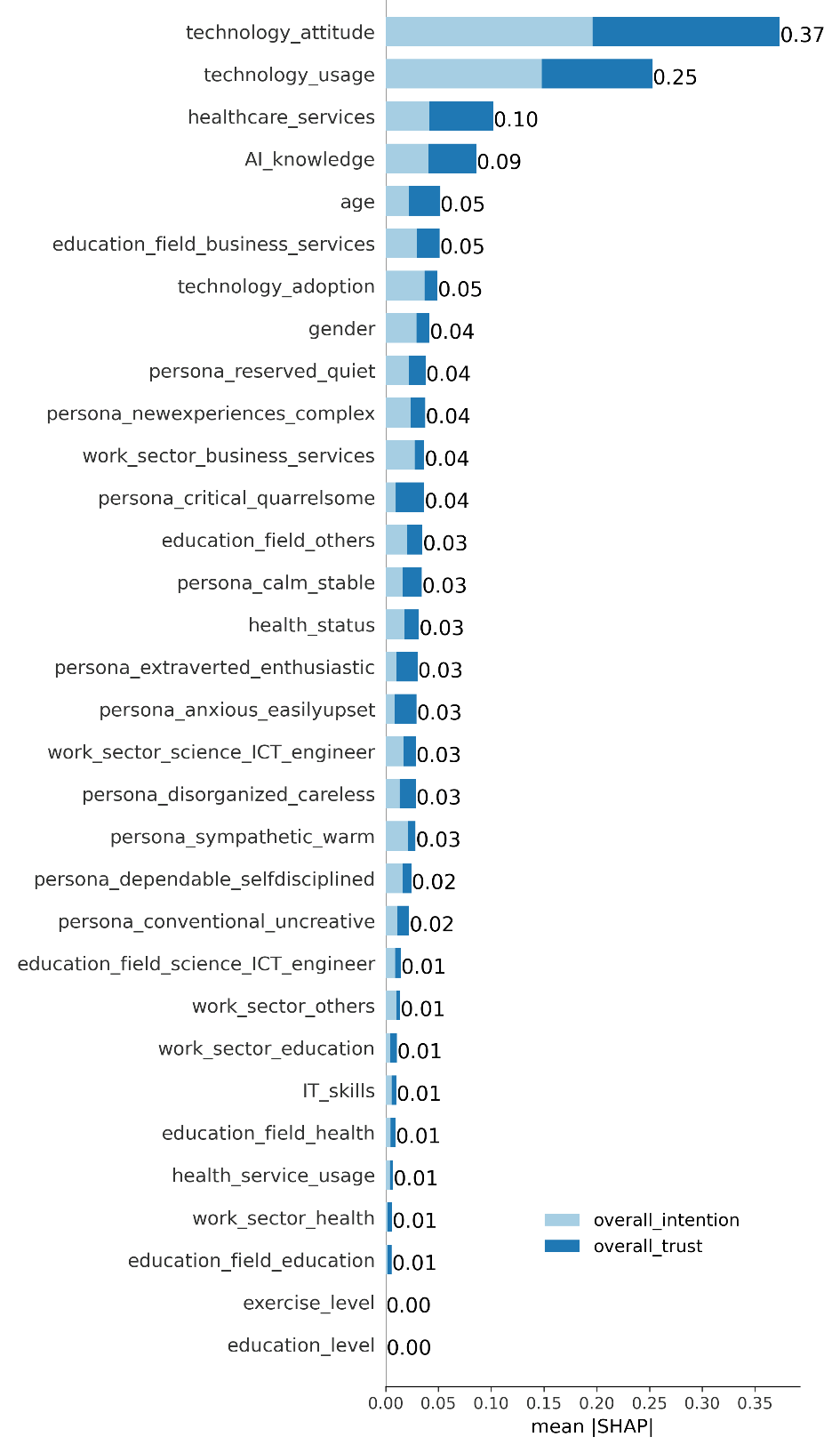


**Figure S2.** Feature importance for predictions of Intention and Trust towards overall usage of AI in healthcare and wellbeing (part 3 of the survey) as measured via SHAP values. Values were computed from all datapoints (n=1086 subjects).

Again, attitude towards AI, usage of AI and knowledge of AI, as well as a view of healthcare services status dominated the prediction (ranks 1-4). The effect of technology_usage, healthcare_services, and technology_attitude was very similar to those shown in Fig. 4 of the article, with a monotonous increasing trend, hence we omit figures here. SHAP values for AI_knowledge and age are depicted in Fig. S3. Unlike for model 1, here the age showed strong non-linearity for Intention and a monotonic increasing trend for Trust (see Fig. 6 in article). For gender, males had a higher predicted impact on Intention (+0.05) and Trust (+0.02) compared to females, yet the overall impact was small (rank 8 in Fig. S3).

| **Intention** | **Trust** |
| --- | --- |
| 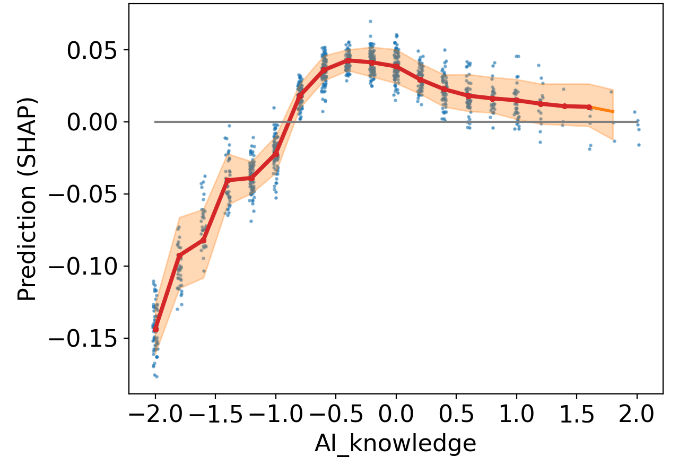 | 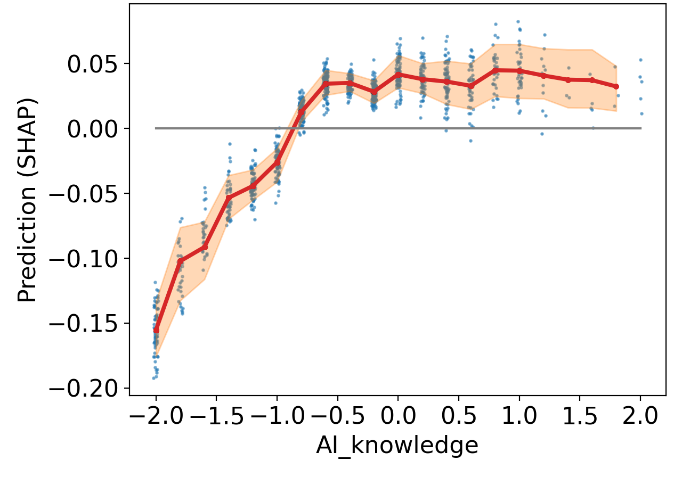 |
| 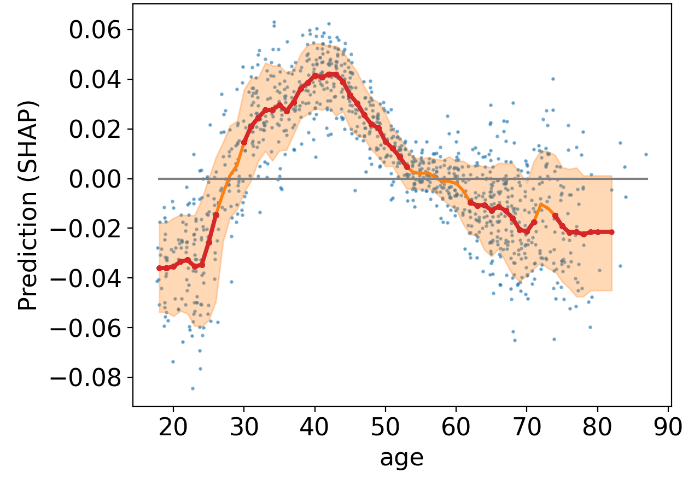 | 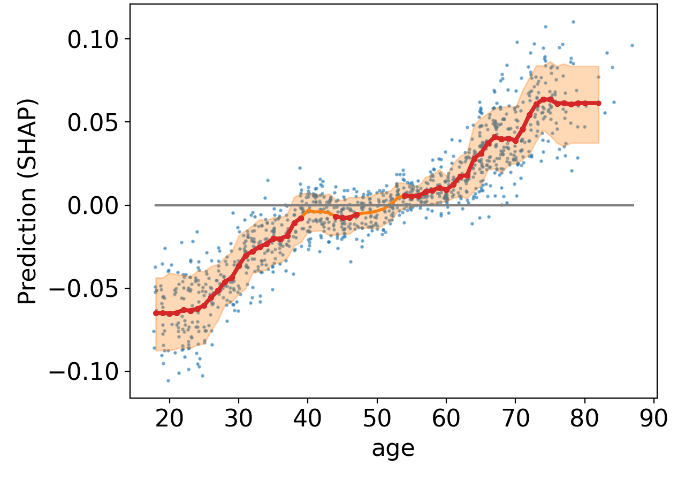 |

**Figure S3**. Impact of age on the Intention and Trust response measured via SHAP main effect values. The shaded region corresponds with the 75% percentile of data. Values were computed from all datapoints (n=1086 subjects).
