# Supplementary material for: Trust and Acceptance Challenges in the Adoption of AI Applications in Health Care: Quantitative Survey Analysis
Source: J Med Internet Res. 2025 Mar 21;27:e65567. doi: 10.2196/65567 (PMC11971584; doi:10.2196/65567)
Supplement: Multimedia Appendix 7 [file jmir_v27i1e65567_app7.docx]

**Pair-wise interaction effects**

In addition to individual, the main effect for predictor variables, SHAP also provides interaction variables between pairs of predictors which allows analysis of complex interplays. The strength of interactions is depicted in Fig. S1 averaged over all output dimensions and in Fig. S2 only for Trust. The top-10 highest values are marked with green crosses.

| (a)  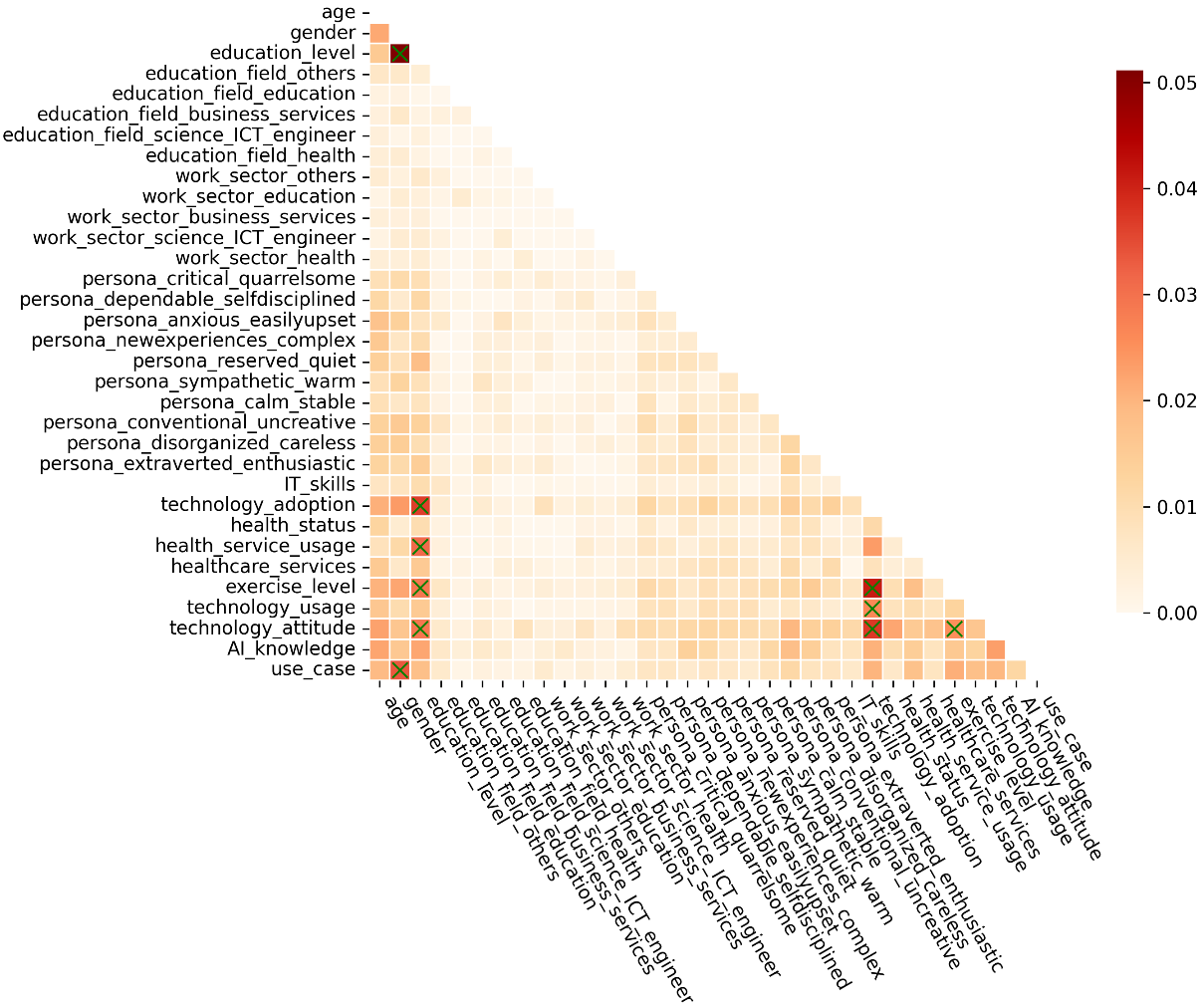 |
| --- |
| (b)  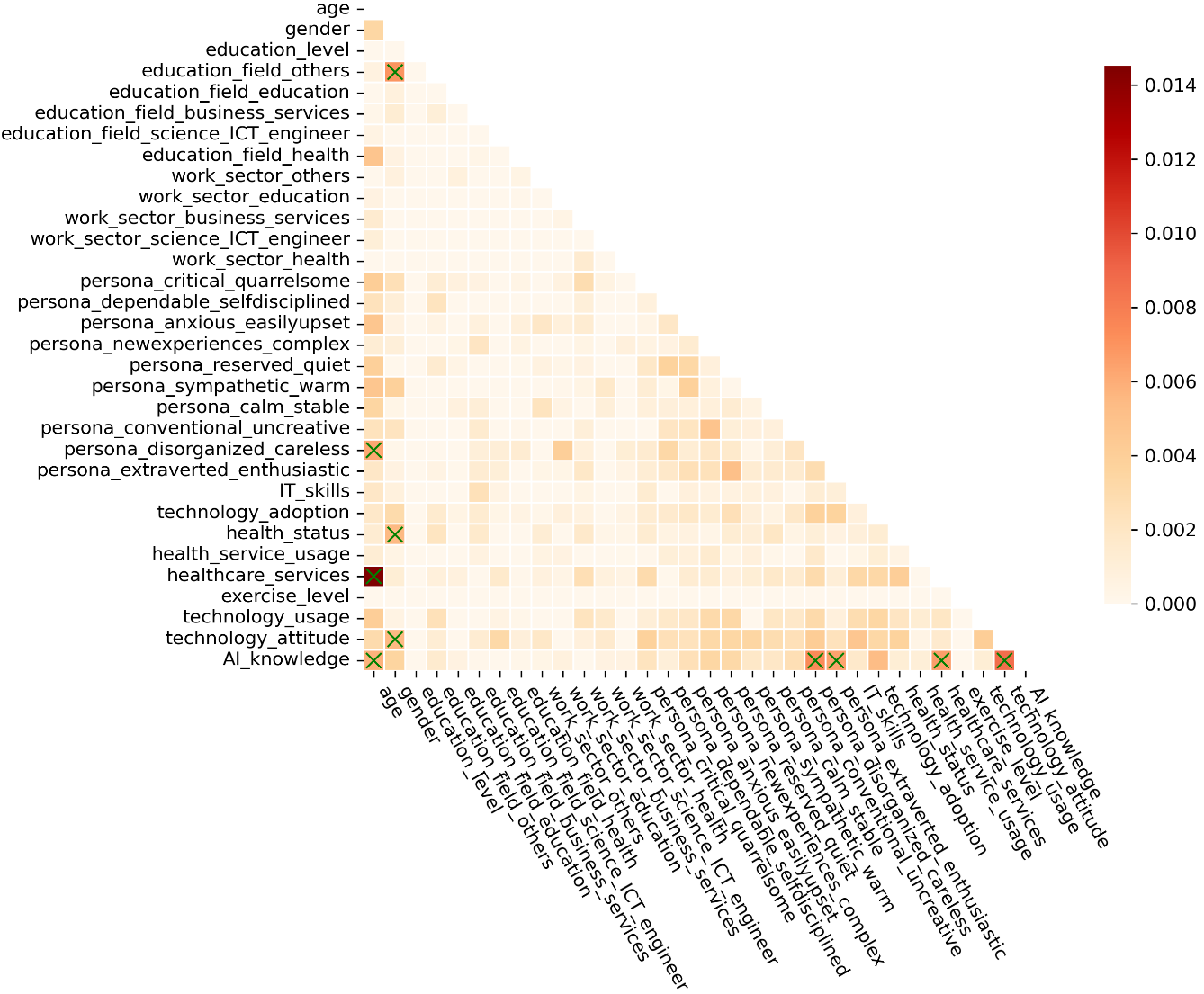 |

**Figure S1.** Interaction strengths for (a) model 1 and (b) model 2 computed via SHAP. Here, SHAP values are averaged over all response dimensions (7 and 2). Colors indicate mean absolute SHAP interaction values.

| (a)  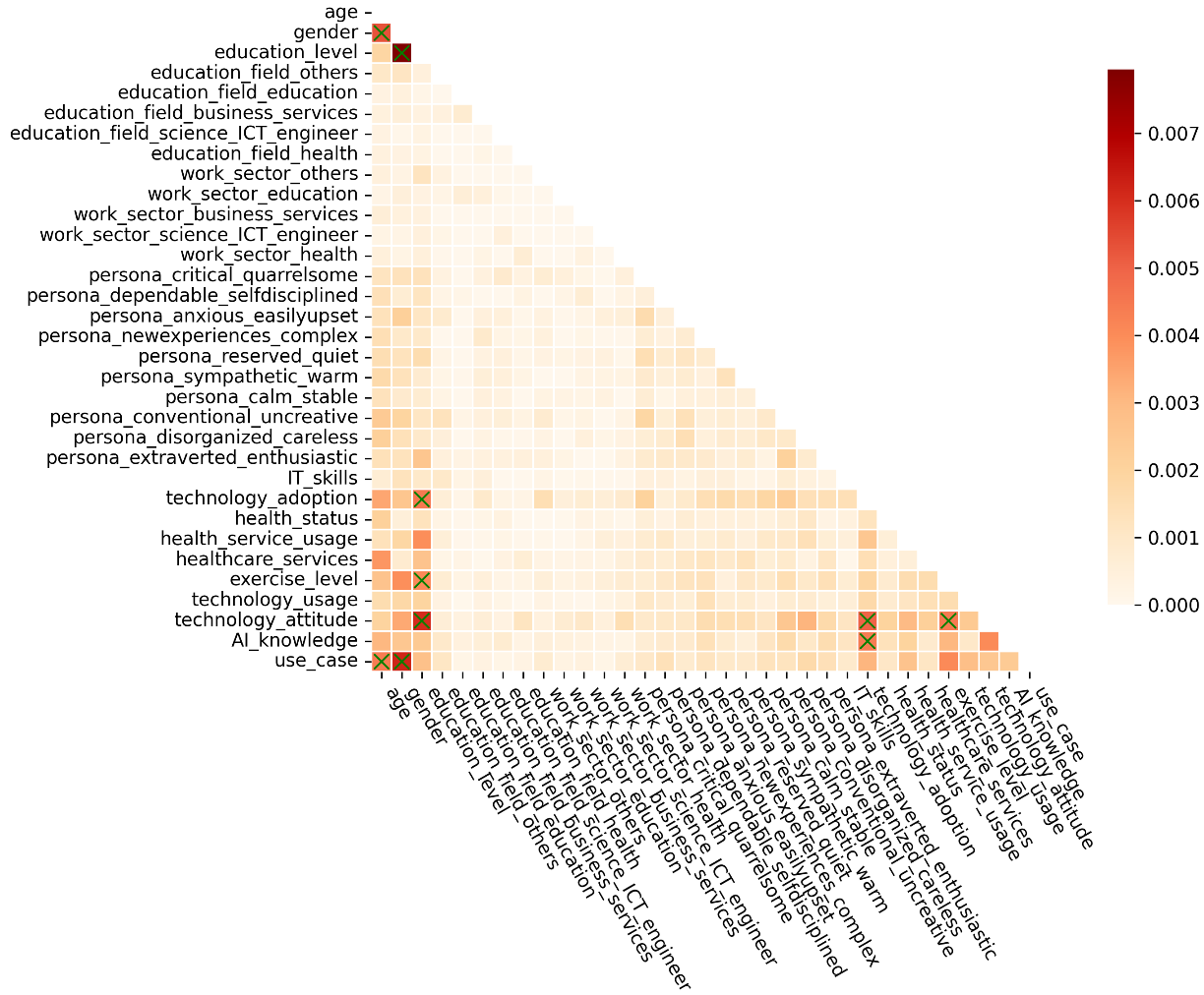 |
| --- |
| (b)  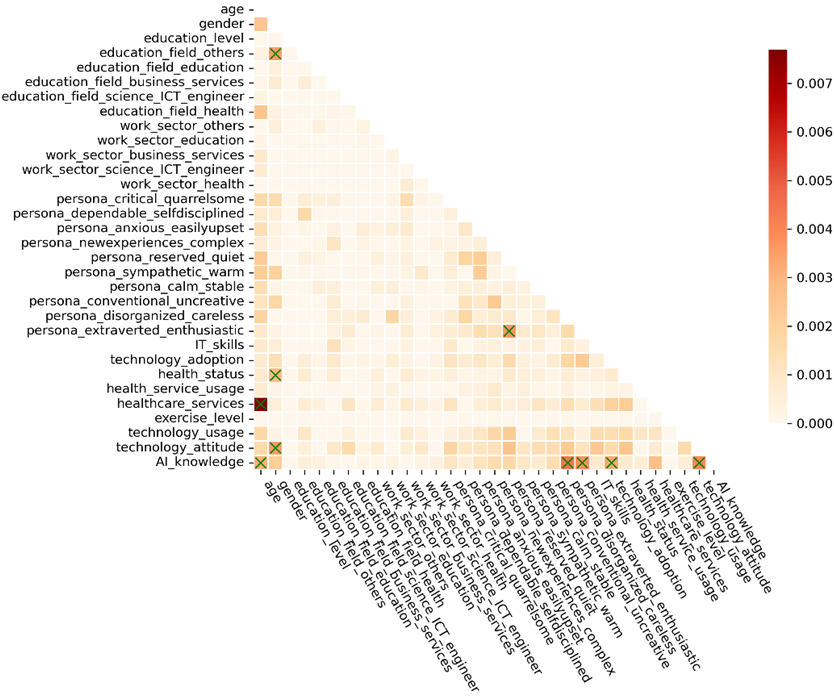 |

**Figure S2.** Interaction strengths for (a) model 1 and (b) model 2 were computed via SHAP for Trust only. Colors indicate mean absolute SHAP interaction values.

Using interaction strengths, we can identify those interactions where variables have notable interplay. Next, we plot some of the strongest interactions for models 1 and 2 for Trust and Intention, and Trade-off for model 1 only. Because of the high correlations between response variables (e.g., Intention and Trust; see Table 1 of the article), many interactions were similar between responses. Hence, we depict those that represent somewhat unique behavior. Interaction plots are useful in revealing some novel effects and outcomes that might be difficult to hypothesize beforehand and compute using only linear regression models. Indeed, we noticed notable nonlinearities and interplays between variables. However, it is important to note that individual interactions only make a small contribution towards total predictions. The typical scale of even the largest SHAP interaction values lies between -0.04 and 0.04, whereas the direct (main effect) SHAP values for variables, as shown in Figures 1-6 of the article, are typically an order of magnitude larger. Hence, one should not over-emphasize the importance of individual interactions; the final predictions result from many smaller contributions.

Figure S3 depicts the following interactions for model 1:

**Trust:** (a) education_level × technology_attitude, (b) gender × technology_attitude, (c) gender × education_level, and (d) gender × age
**Trade-off:** (e) gender × technology_adoption, (f) gender × age, and (g) gender × education_level
**Intention:** (h) gender × exercise_level, (i) gender × education_level, and (j) healthcare_services × age

| **TRUST (model 1)** | |
| --- | --- |
| (a)  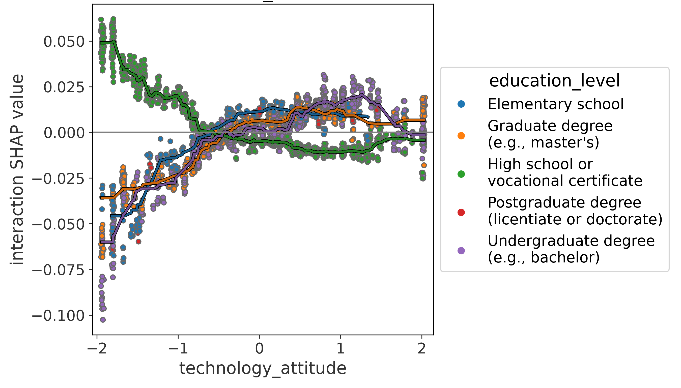 | (b)  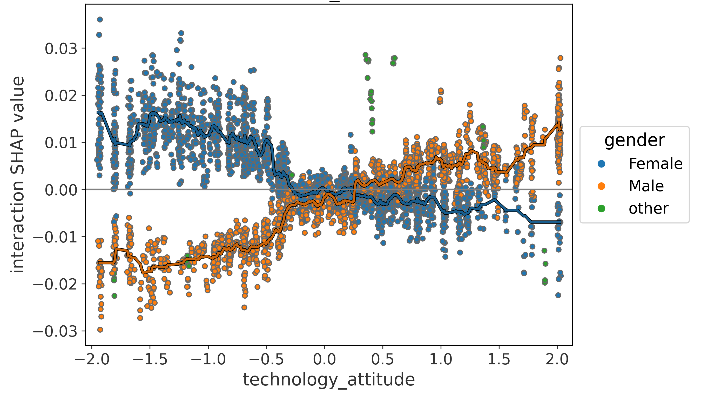 |
| (c)  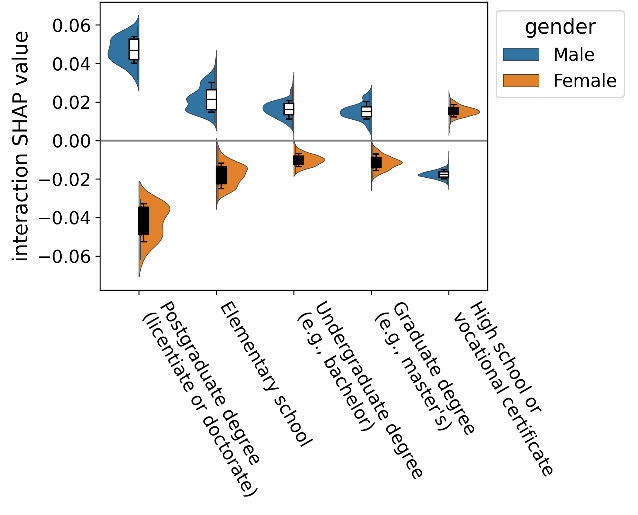 | (d)  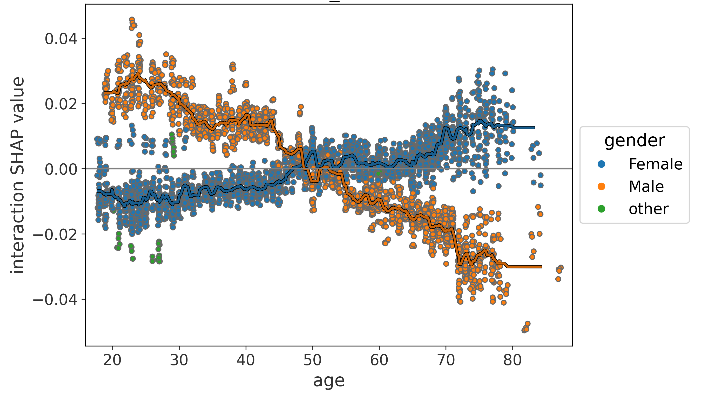 |
| **TRADE-OFF (model 1)** | |
| (e)  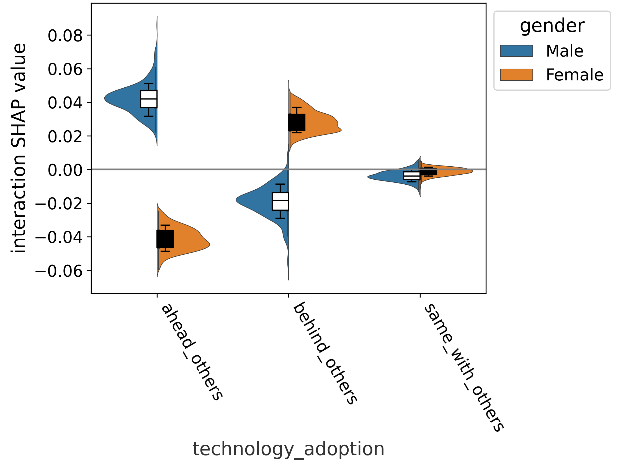 | (f)  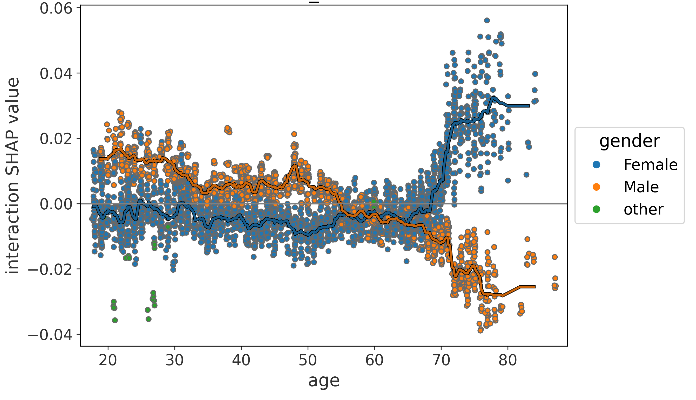 |
| (g)  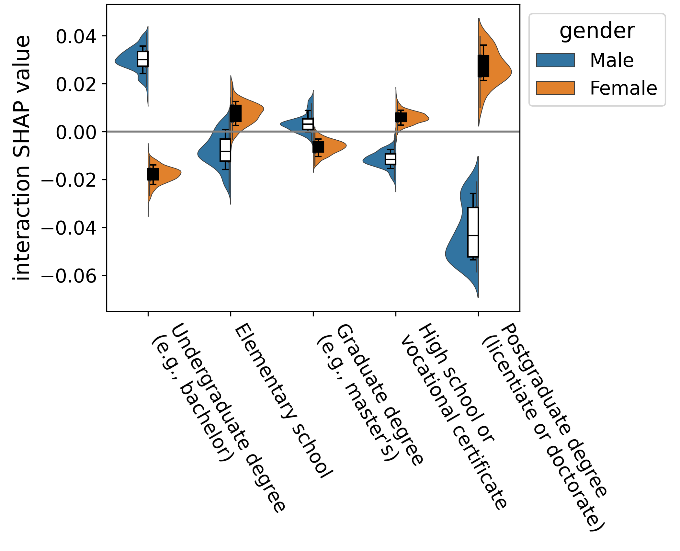 | |
| **INTENTION (model 1)** | |
| (h)  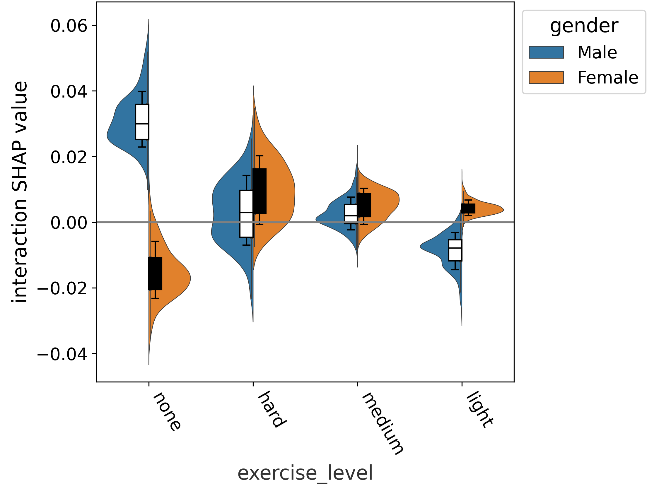 | (i)  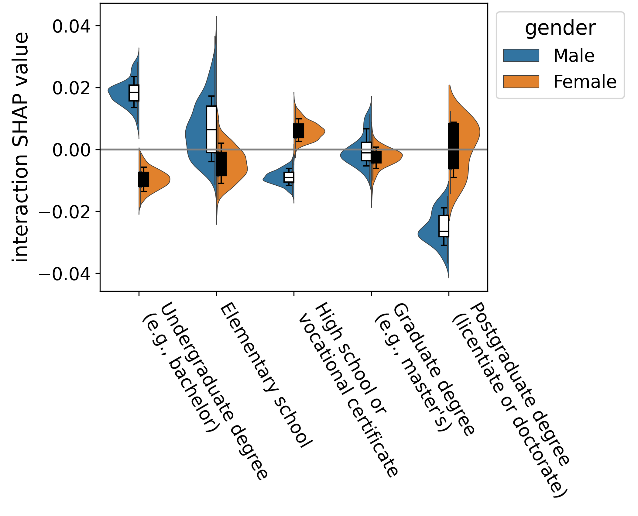 |
| (j)  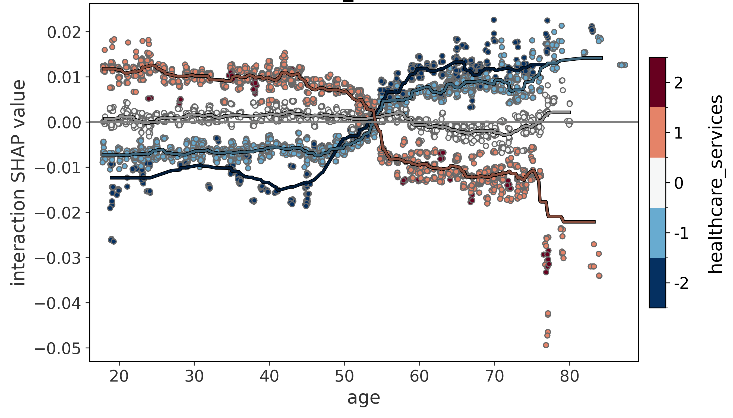 | |

**Figure S3.** SHAP dependency plot for selected pairs of the strongest interactions in predicting Trust, Trade-off and Intention using model 1 (n=5146 with 1100 subjects). For each response, we have plotted the strongest interactions. Lines represent the moving average shown as a guide to the eye. Categories and lines are only shown when enough samples (over 50) are present.

For the model 1, we found the following interaction phenomena:

- The highest education level has a notable impact combined with the lowest level of technoloy_attitude (below -1) so that individuals with high school or vocational certificates have more positive trust (also intention) versus others.
- For the trust, education has a notable effect on males and females. In particular, males with postgraduate degrees have notably higher trust than females. However, the effect is the opposite for trade-off and intention.
- Females with low technology_attitude (below -0.5) have higher trust (also intention) than men
- Females and males have opposite, near-linear trends for trust (also intention), so trust increases with age for females and decreases for males. For a trade-off, the separation between genders only sets in after the age of around 70, where willingness for trade-off increases for females but decreases for males.
- Males with the highest technology_adoption, being ahead of others, have a higher willingness for trade-off compared to female
- Exercise level has a minor effect on intention, trust, and trade-off. Those who do not exercise had gender differences such that males had higher trust, intention, and willingness for the trade-off.
- Age had a notable nonlinear effect depending on the personal opinion of healthcare services (variable healthcare_services) such that a clear transition point occurs between age 50-60; before age 50, the more positive view on healthcare services predicts higher trust, intention, and willingness for the trade-off. However, the situation is opposite after age 60.

Figure S4 depicts the following interactions (marked with “×”) for the model 2:

**Trust:** (a) persona_reserved_quiet × persona_extraverted_enthusiastic, (b) persona_disorganized_careless × AI_knowledge, (c) gender × technology_attitude, and (d) AI_knowledge × technology_attitude
**Intention:** (e) healthcare_services × age, (f) gender × health_status, (g) gender_× technology_attitude, and (h) persona_disorganized_careless × age

| **TRUST (model 2)** | |
| --- | --- |
| (a)  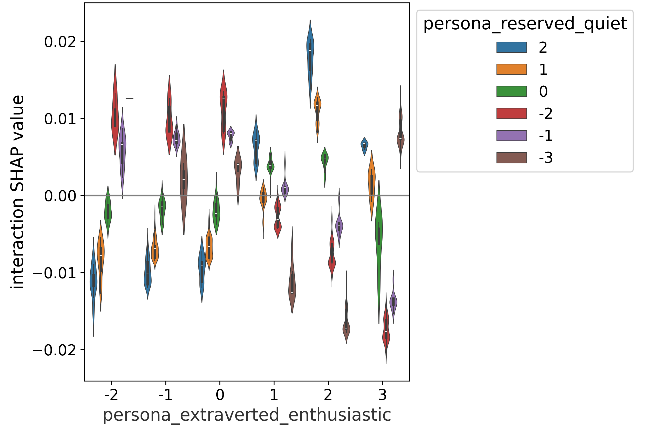 | (b)  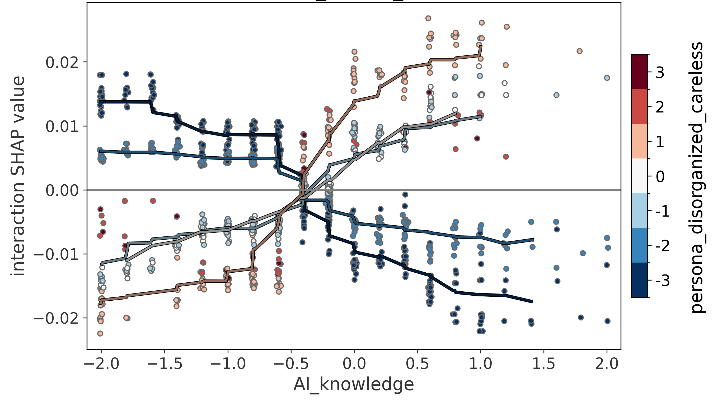 |
| (c)  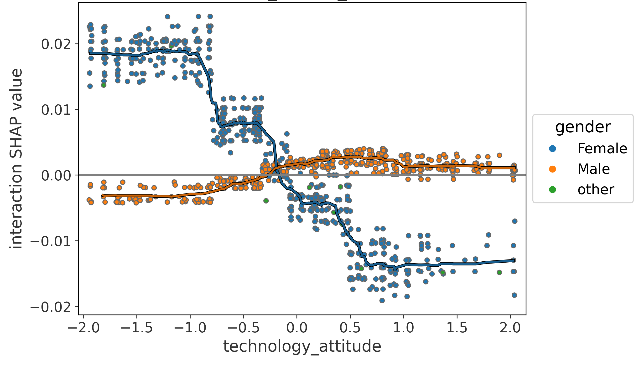 | (d)  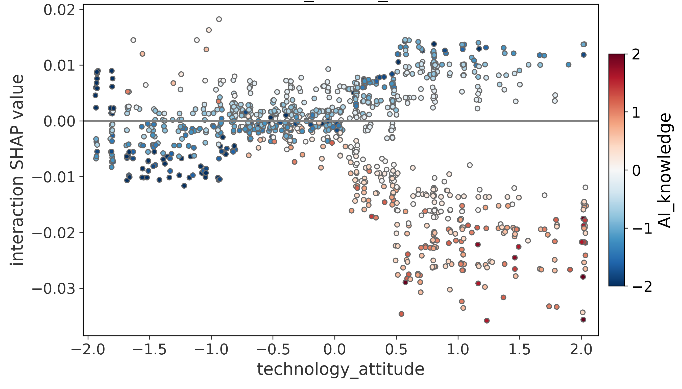 |
| **INTENTION (model 2)** | |
| (e)  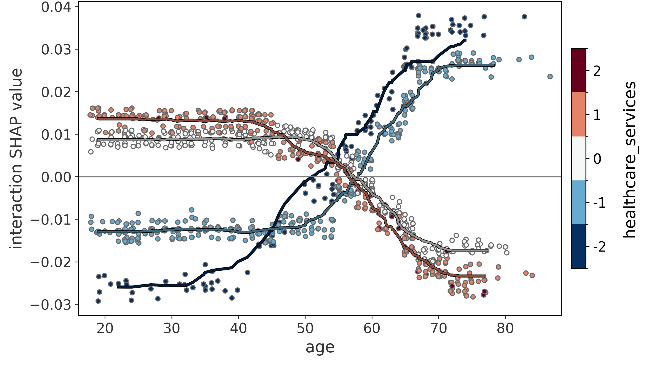 | (f)  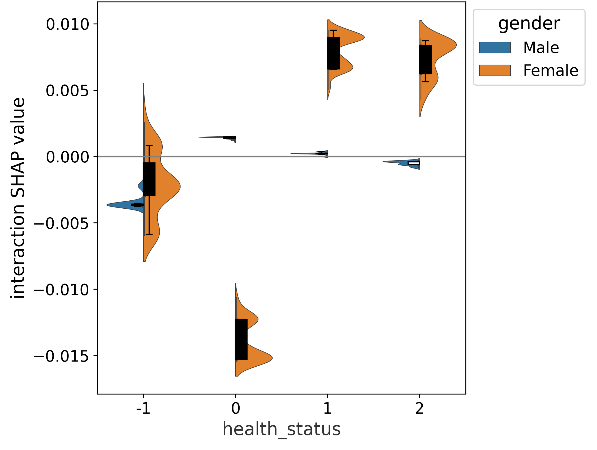 |
| (g)  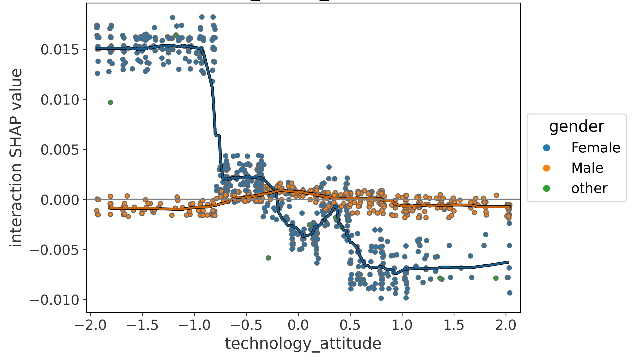 | (h)  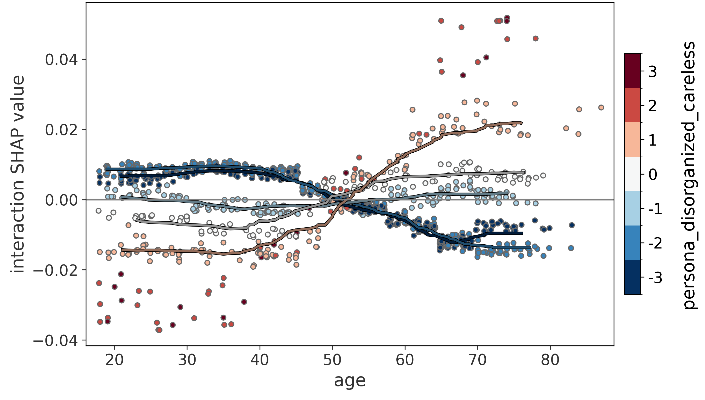 |

**Figure S4.** SHAP dependency plot for interactions in predicting trust and intention using model 2 (n=1086 subjects & data points). For each response, we have plotted the strongest interactions. Lines represent the moving average shown as a guide to the eye. Categories and lines are only shown when enough samples (over 50) are present.
