# Supplementary material for: Trust and Acceptance Challenges in the Adoption of AI Applications in Health Care: Quantitative Survey Analysis
Source: J Med Internet Res. 2025 Mar 21;27:e65567. doi: 10.2196/65567 (PMC11971584; doi:10.2196/65567)
Supplement: Multimedia Appendix 8 [file jmir_v27i1e65567_app8.docx]

Maximal and minimal trust and willingness for trade-off

We used the trained Catboost model 1 to predict how to obtain maximal or minimal Trust and willingness to Trade-off. We ran Optuna with 5000 iterations to find local minima and maxima. In Figs. S1 and S2 depict the contribution of top-20 variables as measured via SHAP for “mental health monitor” and “robot surgeon” use-cases. Variables are sorted in descending order according to total absolute values of SHAP. These examples represent 4 archetypal, hypothetical individuals among the least (-1.83 and -1.49) and most extreme predictions (1.54 and 1.89) found by Optuna.

| Mental health monitor:  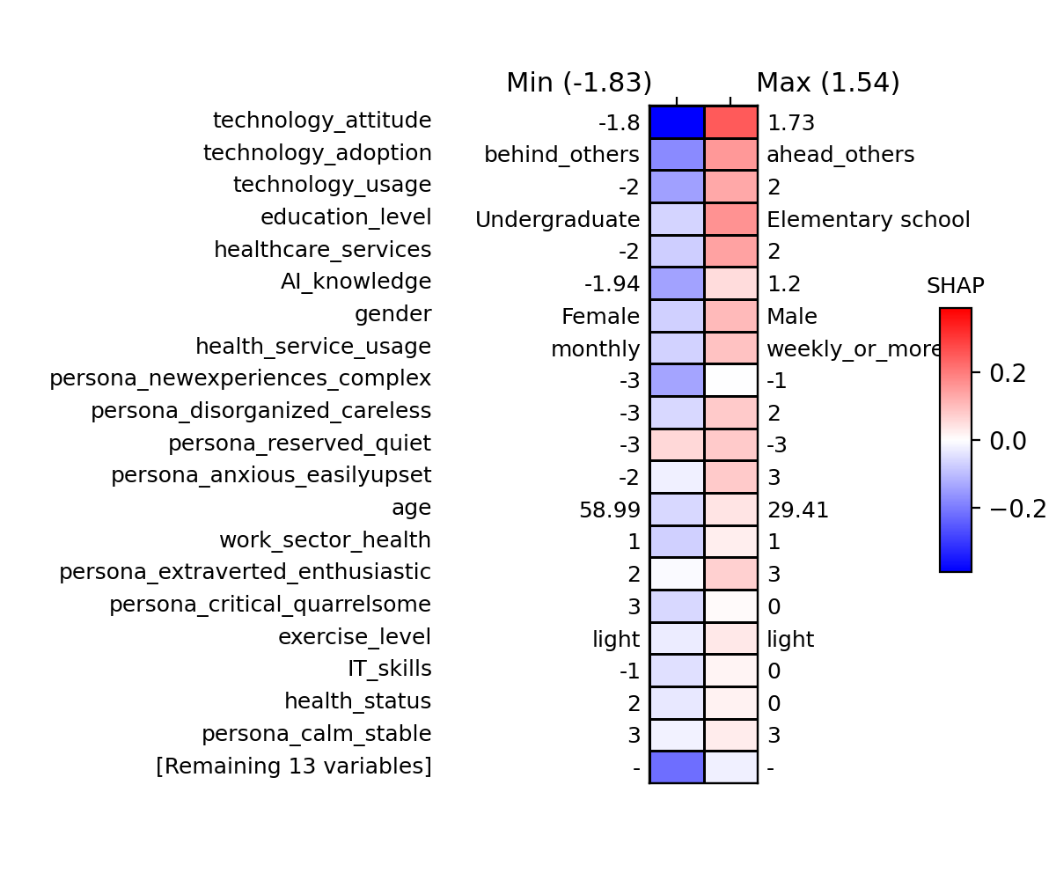 |
| --- |

| Robot surgeon:  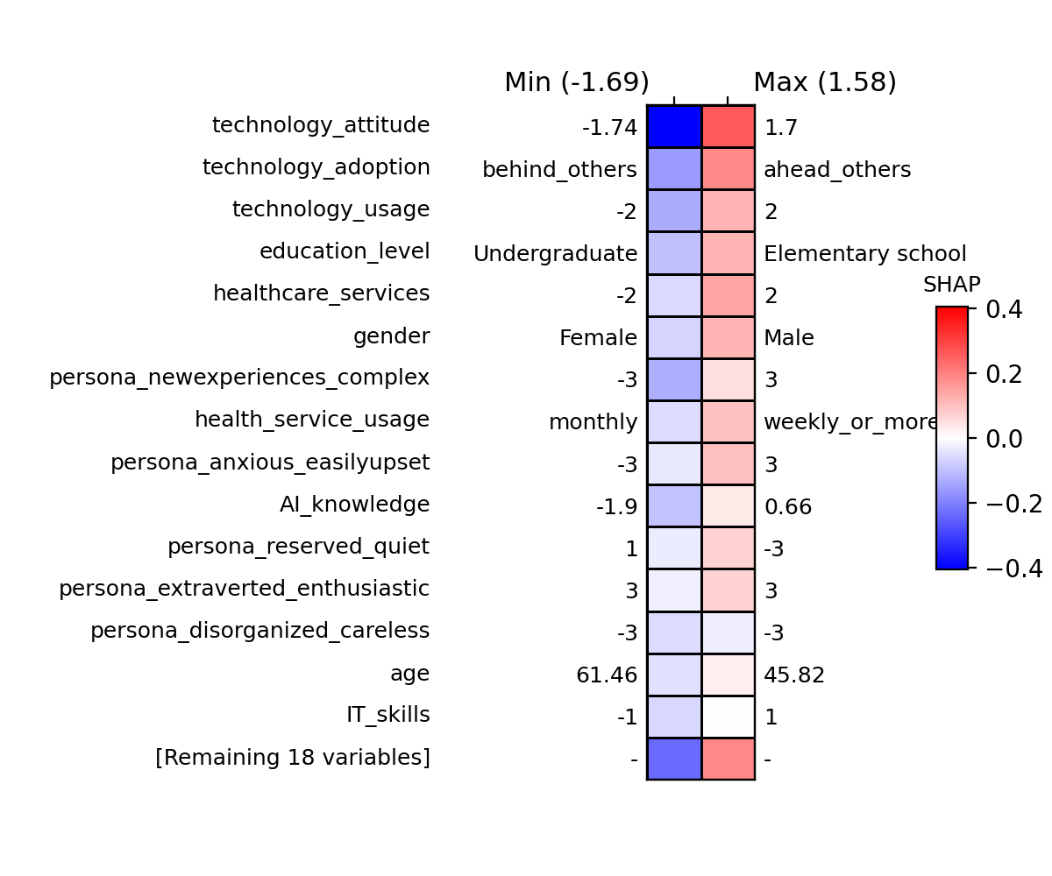 |
| --- |
| Activity monitor:  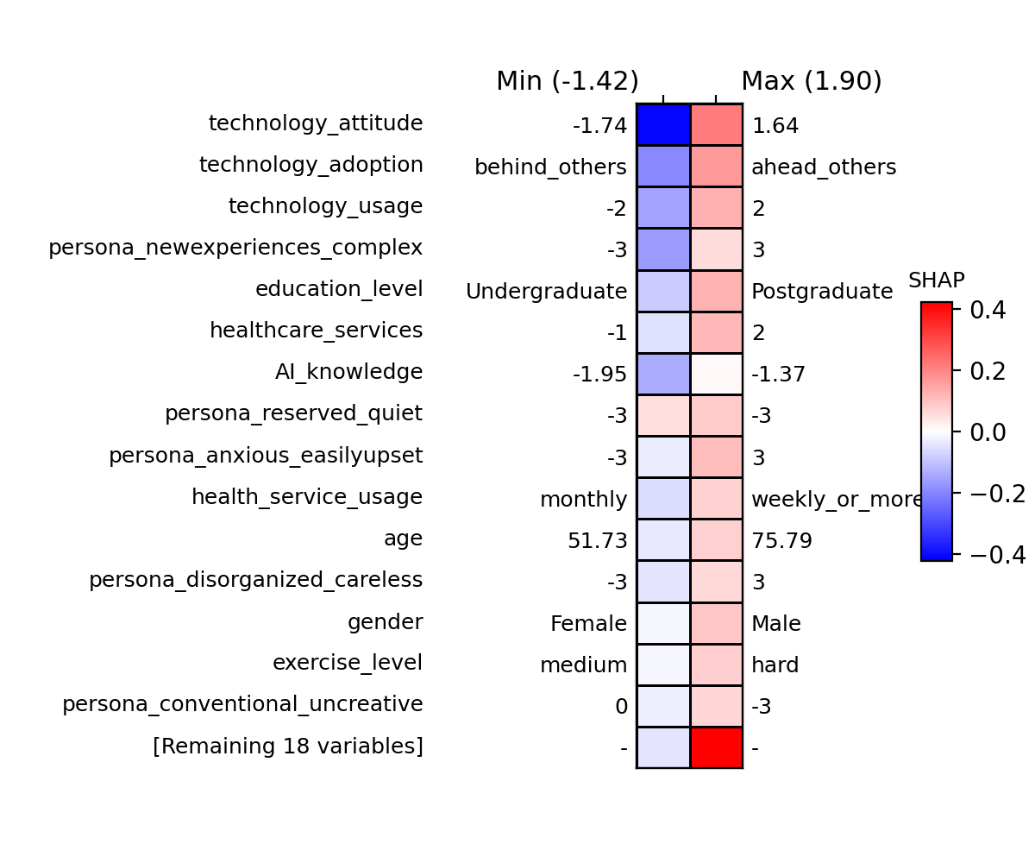 |

**Figure S1.** Examples of the optimized minimum and maximum predictions for Trust for three use cases. The most important 20 variables are shown individually with the remaining 13 pooled together.

| Mental health monitor:  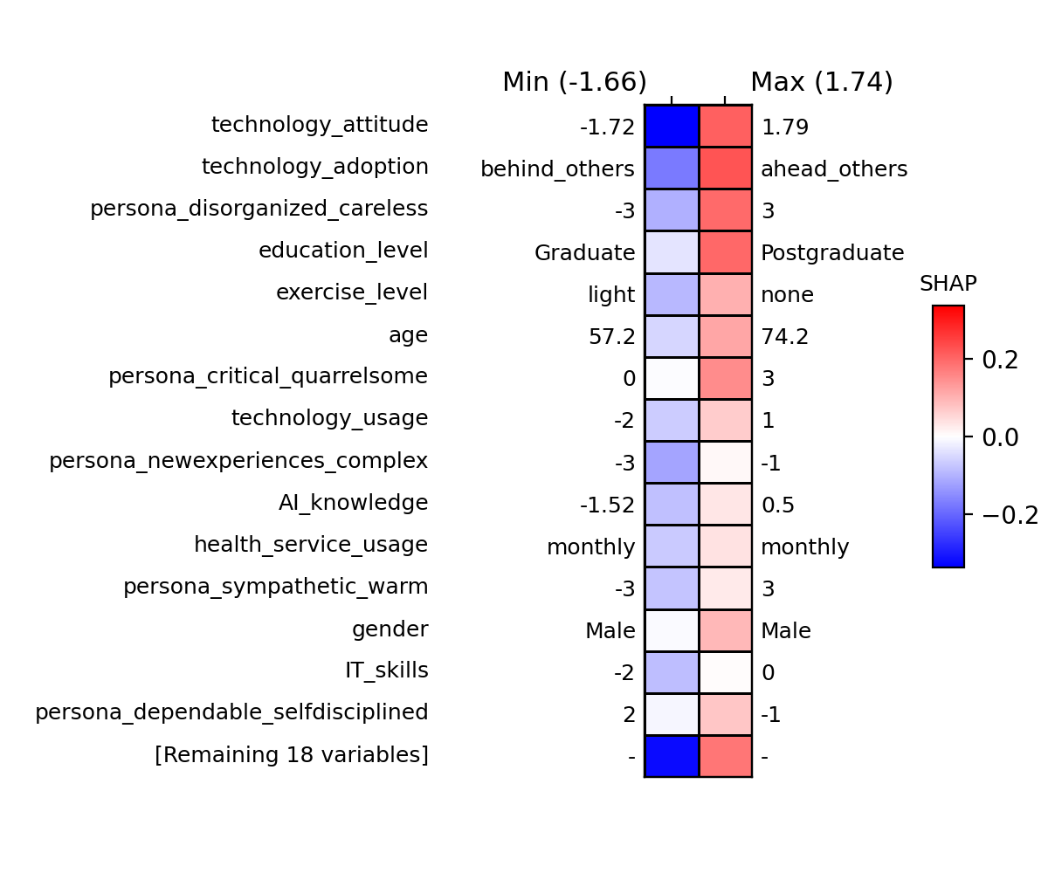 |
| --- |
| Robot surgeon:  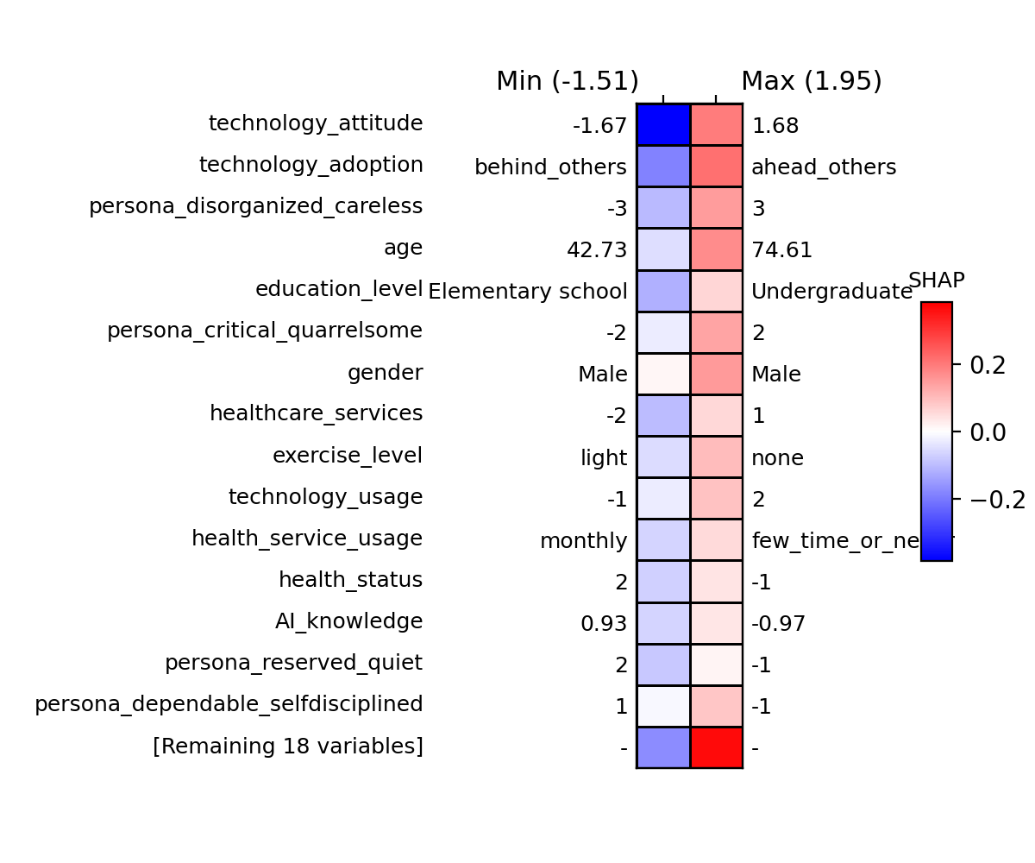 |
| Activity monitor:  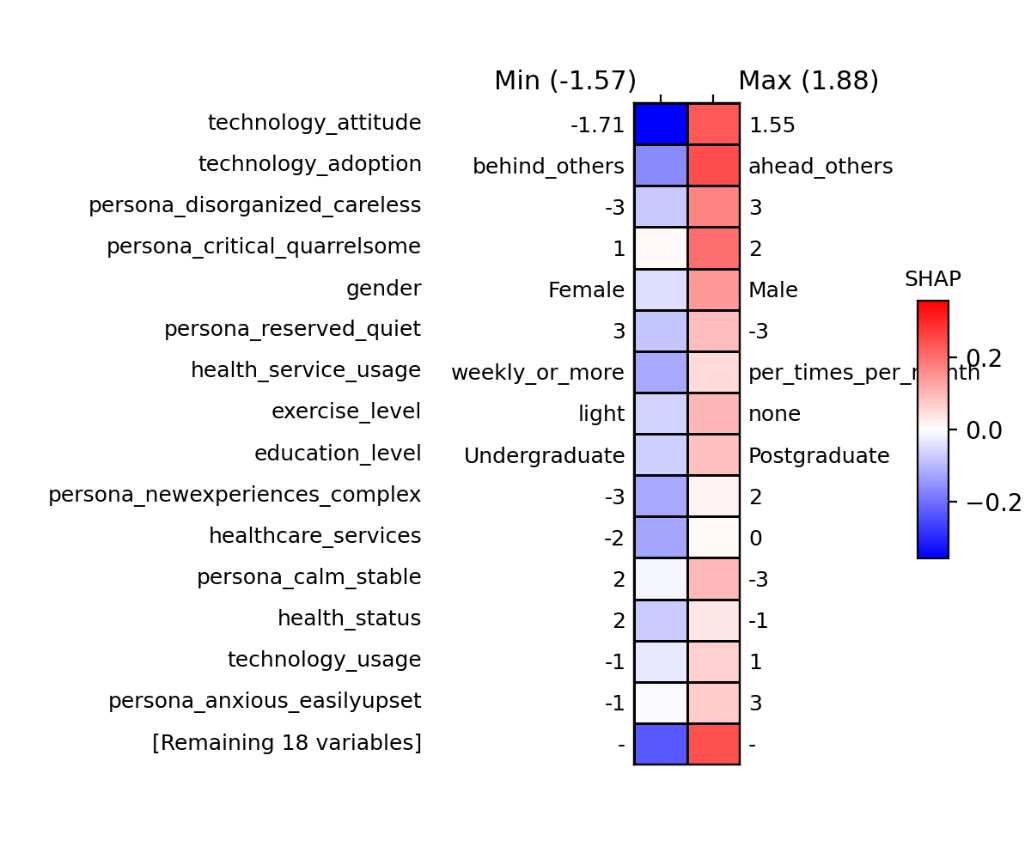 |

**Figure S2.** Examples of optimized minimum and maximum predictions for Trade-off for three use cases. The most important 20 variables are shown individually with the remaining 13 pooled together.

For all cases, the strongest predictors were technology attitude (rank 1) and adoption (rank 2). Yet, personality and gender also had notable influence. Non-linearity and interactions between variables resulted in some of the same values having both negative and positive contributions depending on the context. For example, such variables include work_sector_health (Fig. S1) and AI_knowledge (Fig. S2). These results illustrate the importance of context and interactions between variables. It’s also important to notice these are local maxima/minima and could still be far from global maxima/minima. Therefore, particularly values beyond the first few with less impact on results are likely not optimized and should be considered mostly random.
